# From drug repositioning to target repositioning: prediction of therapeutic targets using genetically perturbed transcriptomic signatures

**DOI:** 10.1093/bioinformatics/btac240

**Published:** 2022-06-27

**Authors:** Satoko Namba, Michio Iwata, Yoshihiro Yamanishi

**Affiliations:** Department of Bioscience and Bioinformatics, Faculty of Computer Science and Systems Engineering, Kyushu Institute of Technology, Iizuka, Fukuoka 820-8502, Japan; Department of Bioscience and Bioinformatics, Faculty of Computer Science and Systems Engineering, Kyushu Institute of Technology, Iizuka, Fukuoka 820-8502, Japan; Department of Bioscience and Bioinformatics, Faculty of Computer Science and Systems Engineering, Kyushu Institute of Technology, Iizuka, Fukuoka 820-8502, Japan

## Abstract

**Motivation:**

A critical element of drug development is the identification of therapeutic targets for diseases. However, the depletion of therapeutic targets is a serious problem.

**Results:**

In this study, we propose the novel concept of target repositioning, an extension of the concept of drug repositioning, to predict new therapeutic targets for various diseases. Predictions were performed by a trans-disease analysis which integrated genetically perturbed transcriptomic signatures (knockdown of 4345 genes and overexpression of 3114 genes) and disease-specific gene transcriptomic signatures of 79 diseases. The trans-disease method, which takes into account similarities among diseases, enabled us to distinguish the inhibitory from activatory targets and to predict the therapeutic targetability of not only proteins with known target–disease associations but also orphan proteins without known associations. Our proposed method is expected to be useful for understanding the commonality of mechanisms among diseases and for therapeutic target identification in drug discovery.

**Availability and implementation:**

Supplemental information and software are available at the following website [http://labo.bio.kyutech.ac.jp/~yamani/target_repositioning/].

**Supplementary information:**

Supplementary data are available at *Bioinformatics* online.

## 1 Introduction

In the process of drug development, the identification of therapeutic targets, biomolecules that lead to therapeutic effects is fundamental (Santos *et al.*, 2017). The identification of therapeutic targets for specific diseases should be both accurate and rapid in order to facilitate efficient drug development (Wang *et al.*, 2020). The initial selection of inappropriate therapeutic targets impacts the entire drug development pipeline, and poor choices greatly reduce the rate at which the efficacy of compounds can be confirmed in humans in Phase II clinical trials (Arrowsmith and Miller, 2013; He *et al.*, 2019; Plenge, 2016). The depletion of therapeutic targets is a major problem and has resulted in the recent stagnation of drug discovery research. Most therapeutic targets that can be easily identified using pathological knowledge have already been thoroughly investigated. The conventional methods used to investigate individual diseases are also limited in their ability to discover novel therapeutic targets.

Over two decades, a large body of medical data about various diseases has been accumulated, in the form of omics data about biomolecules, and chemical data about drugs and small compounds (Duan *et al.*, 2014; Lamb *et al.*, 2006). These big data resources provide a previously unparalleled opportunity to identify novel therapeutic targets with optimum efficiency. Popular approaches to date include the use of single nucleotide polymorphisms (SNPs) and transcriptomic data. SNPs occurring in the protein-coding regions of genes associated with a disease are assumed to alter the function of the gene product, and thus to be potential therapeutic targets (Sabik and Farber, 2017; Shastry, 2007). However, this is not necessarily the case. Since SNP information is static, SNP information alone cannot indicate whether a protein encoded by the SNP-associated gene is activated or repressed: the mechanism of the disease of interest thus remains unknown. Therefore, further analyses that determine if a candidate target should be activated are prerequisite to the treatment of the disease. In transcriptome-based approaches, patterns of gene expression in healthy subjects and patients are compared, with the aim of identifying proteins encoded by abnormally expressed genes. Disease-specific proteins are then assumed to be candidate therapeutic targets (De Vos *et al.*, 2002; Ruiz-Garcia *et al.*, 2010). This approach has led to the identification of a therapeutic target for prostate cancer (Dhanasekaran *et al.*, 2001). However, these approaches tend to predict too many candidates for therapeutic targets, which makes the search space too large to be explored exhaustively.

Drug repositioning—the identification of new indications for known drugs—is an efficient drug discovery approach. Many previous studies have used gene expression data in human cell lines following treatment with drugs. Such drug-induced transcriptomic data are used to identify potential targets and pathways of drugs and then to predict new therapeutic indications for existing drugs (Chen *et al.*, 2015; Iwata *et al.*, 2017). Because the best therapeutic agents for diseases are assumed to facilitate the recovery of impaired cell systems, the focus of research has been placed on the inverse correlation method, looking for drugs that invert the expression profile characteristic of a disease. For example, drugs that are effective for the treatment of inflammatory bowel disease, prostate cancer and colorectal cancer have been discovered using the inverse correlation method (Dudley *et al.*, 2011; Kosaka *et al.*, 2013; Van Noort *et al.*, 2014). In addition to drug-induced gene expression transcriptomic data, large amounts of genetically perturbed transcriptome data, arising from either gene knockdown or gene overexpression experiments, have become available from public databases (Sawada *et al.*, 2018; Subramanian *et al.*, 2017). The genetically perturbed gene expression signatures of therapeutic targets for diseases are assumed to reflect the functions of the drugs targeting those proteins. Similar to the inverse correlation method used in drug repositioning, it is worth investigating the correlation between disease-specific gene expression signatures and genetically perturbed gene expression signatures, to facilitate the identification of therapeutic targets.

**Fig. 1.**
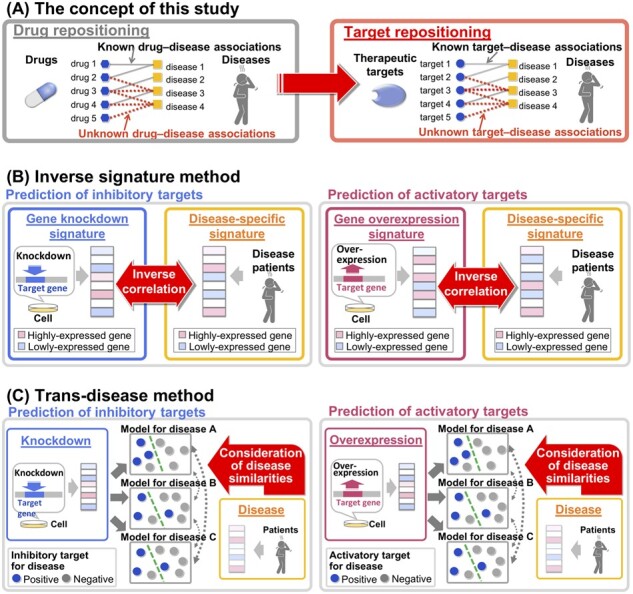
Data processing flow charts of the proposed method for predicting therapeutic targets from transcriptomic signatures. (**A**) The concept of this study: target repositioning, which is an extension of the concept of drug repositioning. Target repositioning predicts new therapeutic target indications for diseases. (**B**) The inverse signature method. Correlation coefficients for inhibitory or activatory target–disease pairs were calculated from gene knockdown and disease-specific signatures or gene overexpression and disease-specific signatures, respectively. (**C**) The trans-disease method. Gene knockdown and gene overexpression signatures were used as input to predictive models for individual diseases. Predictive models are simultaneously learned by sharing the disease similarities of disease-specific signatures

In this study, we propose the novel concept of target repositioning, an extension of the concept of drug repositioning, to predict new therapeutic target indications for a wide range of diseases. Figure 1 provides an overview of our proposed method, and Figure 1A shows the concept of our study. Predictions were performed by a trans-disease analysis based on the integration of genetically perturbed transcriptomic signatures and disease-specific gene transcriptomic signatures. This method allowed us to consider similarities among diseases, and to predict the therapeutic targetability, not only for proteins with known target–disease associations but also for orphan proteins without known associations.

## 2 Methods

### 2.1 Transcriptomic data following genetic perturbation

The L1000 database is a repository of gene expression profiles, maintained by the Library of Integrated Network-based Cellular Signature (LINCS) program (http://www.lincsproject.org) (Subramanian *et al.*, 2017). The gene expression profiles GSE70138 and GSE92742 were obtained from the Gene Expression Omnibus (GEO) database (Barrett *et al.*, 2007). This assay involved a range of cellular perturbations to 93 human cell lines. The L1000 database provided 978 landmark genes, which are referred to as ‘L1000 genes’. Here, we used ‘level 5’ data, which consists of profiles generated by collapsing several replicates.

The gene expression levels were measured at 6, 24, 48, 96, 120, 144 and 168 h after gene knockdown, and 24, 48, 72 and 96 h after gene overexpression, producing a total of 591 855 gene expression profiles. Each profile was represented by its ‘sig_id’. We used 36 720 gene knockdown profiles (denoted as ‘trt_sh.cgs’) and 22 205 gene overexpression profiles (denoted as ‘trt_oe’). We individualized the gene knockdown profiles by averaging the biological replicates, time points and doses. We constructed 4345 gene knockdown profiles on 17 cell lines. We used the same procedure to construct 3114 gene overexpression profiles on 10 cell lines. Supplementary Table S1 shows the list of cell lines.

We constructed gene expression profiles following gene knockdown and gene overexpression, which are referred to as ‘gene knockdown signatures’ and ‘gene overexpression signatures’, respectively. Together, these signatures are referred to as ‘genetically perturbed signatures’. Each gene knockdown and gene overexpression signature was represented as a feature vector, $x^{\mathrm{inh}}\;=\;{\left(x_{1},x_{2},\ldots ,x_{p}\right)}^{T}$ and $x^{\mathrm{act}}\;=\;{\left(x_{1},x_{2},\ldots ,x_{p}\right)}^{T}$, respectively, where *p* is the number of genes. Each element in the signature was defined as the difference between the gene expression value measured after gene perturbation and the value measured in the corresponding control, the background of the plate. Each genetically perturbed signature was affected by the human cell line used, the dose administered and the time point of the experiment.

### 2.2 Transcriptomic data from individuals with disease

The gene expression profiles of patients with various diseases were obtained from the CRowd Extracted Expression of Differential Signatures database (Wang *et al.*, 2016) based on the characteristic direction method (Clark *et al.*, 2014), which compares the gene expression measured in diseased tissue with that measured in control tissue.

In this study, 695 profiles annotated as ‘manual disease signatures’ were used, because these profiles were assigned disease ontology IDs (DOIDs) (Kibbe *et al.*, 2015). The DOIDs were converted to their corresponding KEGG DISEASE database (Kanehisa *et al.*, 2009) IDs using medical subject heading terms or the Online Mendelian Inheritance in Man database (Hamosh *et al.*, 2005). We extracted the profiles obtained from humans for 79 diseases and 14 804 genes. The gene expression profiles of the patients were referred to as ‘patient-specific gene expression signatures’. Supplementary Table S2 shows the diseases that had at least one therapeutic target protein. Thirty-two diseases had at least one inhibitory target protein, whereas 15 diseases had at least one activatory target protein. These diseases included Alzheimer’s disease, inflammatory bowel disease and type II diabetes mellitus.

We averaged multiple patient-specific signatures for the same disease and constructed a disease signature for each of the 79 diseases. The gene expression signature of each disease was represented by a feature vector, $z={\left(z_{1},z_{2},\ldots ,z_{q}\right)}^{T}$, where *q* is the number of genes. Disease signatures were constructed for all genes and the L1000 genes. The disease signature comprising all genes was used to calculate the cosine similarity among the diseases. We also constructed a disease similarity matrix.

### 2.3 Therapeutic target data

Therapeutic target information was constructed by manually curating data in medical monographs (Papadakis *et al.*, 2014) and the KEGG DISEASE database (Kanehisa *et al.*, 2009). In total, 525 target–disease associations involving 224 inhibitory target proteins and 32 diseases were used as gold standard inhibitory target data, and 37 target–disease associations involving 30 activatory target proteins and 15 diseases were used as gold standard activatory target data.

### 2.4 SNP profiling method for therapeutic target prediction

Information about disease-associated SNPs is typically utilized to identify therapeutic targets (Sabik and Farber, 2017; Shastry, 2007). The assumption underlying this approach is that diseases are caused by functional changes to the proteins encoded by genes containing SNPs within their coding regions; thus, the genes are regarded as potential therapeutic targets. We used this SNP-based approach as the baseline method and referred to it as the ‘SNP profiling method’.

SNP data for the various diseases were downloaded from the NHGRI-EBI genome-wide association studies (GWAS) catalog database (Buniello *et al.*, 2019). This database provides information about SNP–disease associations, in which SNPs identified in GWASs are registered with their identifiers, associated genes, and the corresponding *P* values. In this study, 142 gene–disease associations involving 77 genes, and 21 diseases were used in the performance evaluation.

We constructed disease-specific SNP profiles, which were referred to as ‘SNP profiles’. When a gene had multiple SNPs or was reported by multiple GWASs, we averaged the *P* values for the gene. We constructed SNP profiles corresponding to the gold-standard data by assigning the value 0 to genes with no SNP data.

We used -log⁡(P) values as predictive scores. Genes that had SNPs with significantly strong associations with disease were considered to be candidate therapeutic targets. Since this method depends on the presence or absence of SNPs in gene-coding regions, it cannot be used to predict whether a therapeutic target is inhibitory or activatory. The performance assessments of four alternative SNP prioritization strategies are included in Supplementary Methods and Results due to space limitations.

### 2.5 Inverse signature method for therapeutic target prediction

The inverse signature method is a popular transcriptome-based drug repositioning approach used to identify novel drugs for the treatment of diseases (Dudley *et al.*, 2011; Jahchan *et al.*, 2013; Kunkel *et al.*, 2011; Sirota *et al.*, 2011). Drug signatures are generally assumed to have an inverse correlation with disease signatures if the drugs have therapeutic effects on those diseases (Van Noort *et al.*, 2014).

In this study, we used the concept of inverse correlation for target repositioning to predict new applications of existing targets to different diseases. Genetically perturbed gene expression signatures were assumed to reflect the functions of the drugs targeting those genes. Therefore, potential inhibitory and activatory target–disease associations were predicted based on the inverse correlations between the gene knockdown or gene overexpression signatures and the disease signatures.

We calculated the correlation coefficient between the gene knockdown signatures, $x^{\mathrm{inh}}$, and disease signatures, z, for each of the inhibitory targets and diseases, and between the gene overexpression signatures, $x^{\mathrm{act}}$, and disease signatures, z, for each of the activatory targets and diseases. Pearson’s correlation coefficient, $r_{\mathrm{xz}}$, was calculated as

#(1) rxz=∑i=1d(xi-x¯)(zi-z¯)∑i=1d(xi-x¯)2∑i=1d(zi-z¯)2,

where $x^{\mathrm{inh}}$ and $x^{\mathrm{act}}$ were described as x, and *d* represents the number of genes common to the genetically perturbed and disease signatures, d=884. Target–disease pairs that had high inverse correlations were considered to be candidate therapeutic targets.

### 2.6 Trans-disease method for therapeutic target prediction

We developed a trans-disease method that can predict therapeutic targets for diseases from genetically perturbed gene expression signatures and disease similarity. Note that there are a number of candidates for diseases, and different diseases may have common characteristics in terms of molecular mechanisms. The same therapeutic targets are sometimes used for multiple diseases. Thus, we propose formulating the problem of therapeutic target prediction in the framework of supervised multiple label prediction (Bickel *et al.*, 2008).

Suppose that there are M diseases and we are given N targets. We consider predicting which diseases would be treated by a target, that is, the ith target (i=1, 2,…,N). Each target is represented by a d-dimensional feature vector as $x_{i}$ in this study, where $x_{i}$ is a genetically perturbed gene expression signature.

We constructed a learning set of target–disease pairs that are pairs given in target–disease associations. There are M candidates for diseases, and each target in the learning set is assigned a binary class label representing the mth disease (m=1, 2, …, M). Let $y_{m,i}\in \left\{0,\;1\right\}$ be the class label for the mth disease assigned to the ith target, where $y_{m,i}=1$ means that ith target is used for the mth disease, and $y_{m,i}=0$ means that the i-th target is not used for the mth disease.

We construct a predictive model to predict whether the ith target would be used for the mth disease. Linear models are a useful tool to analyze extremely high-dimensional data for both prediction and feature extraction tasks. Thus, we adopt a linear function defined as $f_{m}\;=\;w_{m}^{T}x$, where $w_{m}$ is a d-dimensional weight vector for the mth disease. We represent a set of M model weights by a d × M matrix defined as $W:=[w_{1},\;w_{2},\;\ldots ,\;w_{M}]$ and estimate the weight matrix W by minimizing an objective function using the gradient descent method.

To overcome the scarcity of existing knowledge on the relationships between targets and diseases, we performed learning of individual models, $f_{1},f_{2},\ldots ,f_{M}$, jointly, by sharing information across *M* diseases.

We attempt to estimate all of the weight vectors $w_{1},w_{2},\;\ldots ,\;w_{M}$ jointly in the models by minimizing the logistic loss as follows:

#(2) RW=∑m=1M∑i=1Nlog⁡1+exp⁡-ym,iwmTxi.

We introduce a regularization term Ω(W) to the loss function to enhance the generalization properties in the optimization problem as follows:

#(3) minW⁡RW+ΩW.

Here, we introduce two regularization terms. First, we use a standard ridge regularization term to avoid the overfitting problem as follows:

#(4) Ωr:=12TrWWT,

where Tr indicates trace operation.

Second, we design a regularization term reflecting the similarities among diseases. A multitask/multilabel regularizer based on task/label similarities is useful for analyzing related tasks (Evgeniou, 2005). We evaluate the similarity among diseases using the cosine similarity based on disease signatures and construct an M×M similarity matrix S for diseases in which each element $S_{i,j}$ is a similarity score between the ith and jth diseases. Then, we introduce the following regularization term:

#(5) ΩsW:=14∑l=1M∑m=1MSl,mwlKl,l-wmKm,m=12TrWLsWT,

where $\left\|\cdot \right\|$ is the Euclidean norm, K is a diagonal matrix defined as $K_{l,l}:=\sum_{m=1}^{M}S_{l,m}$, and $L_{s}$ is a symmetric normalized Laplacian defined as $K^{-1/2}(K-S)K^{-1/2}$. $\Omega_{s}\left(W\right)$ has the effect of bringing the weight vectors $w_{i}$ and $w_{j}$ close to each other if $S_{l,m}$ is high. Finally, we introduce the following regularization term in the optimization problem (3):

#(6) ΩW:=λsΩsW+λrΩrW,

where $\lambda_{s}\geq 0$ and $\lambda_{r}\geq 0$ are hyper-parameters to control the strength of the regularization terms $\Omega_{S}$ and $\Omega_{r}$, respectively. Further details can be found in the Supplementary Methods.

### 2.7 Data completion method

We imputed the missing entries of the genetically perturbed gene expression signatures using a tensor decomposition algorithm (Iwata *et al.*, 2019). Genetically perturbed gene expression data can be represented by a third-order tensor. Gene expression data collected following gene knockdown consisted of 224 knocked down genes, 978 genes and 17 cell lines, and can be represented as a 224 × 978 × 17 tensor. Similarly, gene expression data collected following gene overexpression consisted of 30 overexpressed genes, 978 genes and 10 cell lines, and was represented as a 30 × 978 × 10 tensor. Most parts of these tensors are missing or unobserved. The missing rates in each cell line are shown in the bottom panel of Supplementary Figures S4–S6.

## 3 Results

### 3.1 Gene expression profiles following gene perturbation are associated with many potential therapeutic targets

**Fig. 2.**
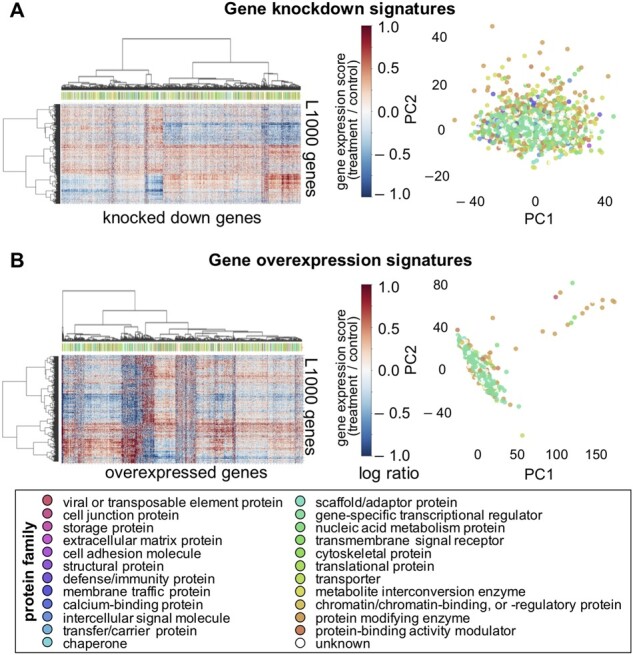
Heatmap and PCA plot of (**A**) gene knockdown and (**B**) gene overexpression signatures. The heatmaps represent the results of hierarchical clustering of both types of perturbed genes of proteins (4,345 knocked down and 3,114 overexpressed genes) and L1000 genes (978 landmark genes from the L1000 database). The color bars to the right of the heatmaps represent the levels of gene expression ratios. The colored labels under the dendrograms represent protein families corresponding to the genetically perturbed genes. High-resolution images of the dendrograms of genetically perturbed genes and the genes belonging to each cluster can be found in Supplementary Figure S2 and Tables S3 and S4, respectively. The PCA plots represent the distribution of genetically perturbed genes labeled based on their protein families, which are represented by different colors

We examined the features of the gene knockdown signatures of 4345 proteins and the gene overexpression signatures of 3114 proteins. We performed hierarchical clustering of the perturbed genes coding for the proteins using the Ward method. We also performed principal component analysis (PCA) on the genetically perturbed gene expression signatures. Figure 2 shows the heatmaps and PCA plots of gene knockdown and gene overexpression signatures, labeled based on protein families provided by the PANTHER resource (Mi *et al.*, 2013; Thomas *et al.*, 2003). Some proteins from different families clustered into the same group, whereas some proteins from the same family clustered separately. Thus, the effects of gene perturbation on proteins appear to be independent of the original protein families, to some extent, and proteins with similar perturbation patterns are likely to have similar associations with diseases, regardless of the sequence-based protein families.

**Fig. 3.**
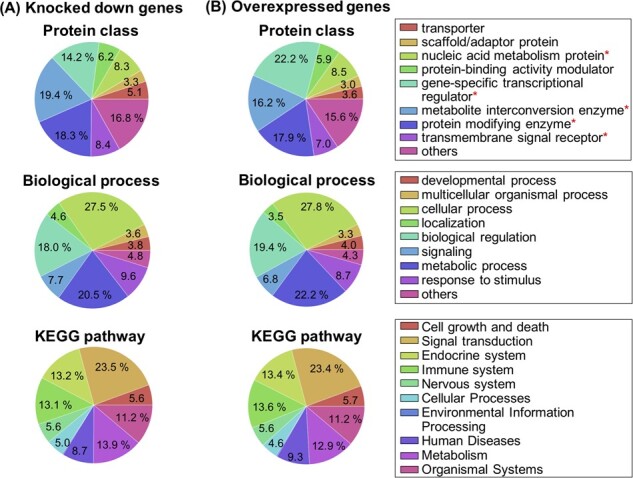
Classifications of perturbed genes according to protein class, biological process, and KEGG pathway. (**A**) Knockdown genes. (**B**) Overexpressed genes. The top panels show a classification based on protein family in PANTHER. Asterisks indicate the top five protein families; the subfamilies belonging to these protein families are shown in Supplementary Figure S3. The middle panels show a classification based on GO biological process. The bottom panels show a classification based on KEGG pathways; each label represents a pathway category

To evaluate whether the perturbed genes contained a sufficient number of candidate therapeutic targets, we examined the molecular functions and biological processes of the products of these genes. Figure 3 shows the classifications of the perturbed genes coding for proteins according to the protein class, biological process, and KEGG pathway. Figure 3A shows the classifications of the 4345 knocked down genes according to protein class, biological process, which were classified based on the Gene Ontology (GO) Biological Process terms, and the KEGG pathway (Kanehisa *et al.*, 2002). For the classifications based on protein family, the knocked down genes were classified using PANTHER. The most commonly found protein class was ‘metabolite interconversion enzyme’, followed by ‘protein modifying enzyme’, ‘gene-specific transcriptional regulator’, ‘transmembrane signal receptor’, ‘nucleic acid metabolism protein’, ‘protein-binding activity modulator’ and ‘transporter’, accounting for 80% of all proteins. The category ‘metabolite interconversion enzyme’ (19.4%) contained protein subfamilies such as kinases, dehydrogenases, reductases and cyclases (Supplementary Fig. S3A). ‘Protein modifying enzyme’ (18.3%) contained protein subfamilies such as proteases and kinases. ‘Gene-specific transcriptional regulator’ (14.2%) and ‘nucleic acid metabolism protein’ (8.3%) contained various transcription factors, nucleic acids and related proteins. ‘Transmembrane signal receptor’ (8.4%) contained subfamilies such as ‘G-protein coupled receptor’ and ‘serine/threonine protein kinase receptor’. These subfamilies in the top protein families are often targets for first-in-class drugs (Eder *et al.*, 2014). The most common term among GO biological processes was ‘Cellular process’, followed by ‘metabolic process’ and ‘biological regulation’, accounting for 66% of the total. These GO terms contained those associated with ‘metabolic reaction’, ‘signaling transduction’ and ‘cell-to-cell communication’. The most common term among the KEGG pathway was ‘Signal transduction’, followed by ‘metabolism’ and ‘endocrine system’. These GO terms and KEGG pathways play important roles in regulating many biochemical reactions that are effective target processes for many drugs. These results suggest that the knocked down genes in this analysis contained many potential therapeutic targets, in terms of both biological processes and molecular functions.

Figure 3B shows the classifications of 3114 overexpressed genes of proteins according to protein family, biological process and KEGG pathway. ‘Gene-specific transcriptional regulator’ was the most commonly found protein family, followed by ‘protein modifying enzyme’, ‘metabolite interconversion enzyme’, ‘nucleic acid metabolism protein’ and ‘transmembrane signal receptor’, accounting for 71.8% of all proteins. Similar to the knockdown gene analysis, there were many protein subfamilies that are commonly used as drug targets (Eder *et al.*, 2014) (Supplementary Fig. S3B). These results suggest that overexpressed genes contain many genes that are potential therapeutic targets, according to their biological processes and molecular functions.

### 3.2 Performance evaluation of therapeutic target predictions

We evaluated the performance of our proposed methods for therapeutic target prediction, the inverse signature and trans-disease methods, using gold standard data (see Section 2). We also tested the ability of a data completion process to overcome the problem of missing data. To evaluate the performance, we used receiver operating characteristic (ROC) curves for the performance of a classifier over all possible cutoffs, by plotting true positive rates (TPRs) against false-positive rates (FPRs). The area under the ROC curve (AUC) score ranges from 0 to 1.0, with 1.0 indicating perfect inference, (100% TPR and 0% FPR) and 0.5 representing random inference. The AUC scores for all target–disease pairs were calculated for each cell line. We evaluated the performance of therapeutic target predictions with the trans-disease method by 5-fold cross-validation experiments. We compared the performance of the proposed methods with that of a baseline method, the SNP profiling method.

**Fig. 4.**
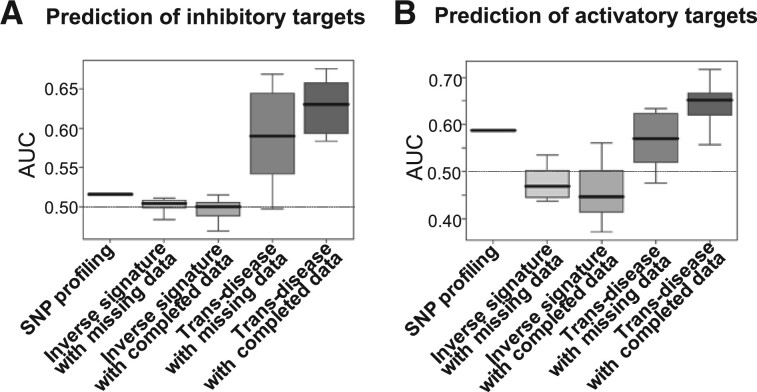
Comparisons of the performance of the baseline method (SNP profiling) and the proposed methods. (**A**) Inhibitory target predictions. (**B**) Activatory target predictions. The proposed methods are the inverse signature and trans-disease methods, with missing and completed data. Each box plot represents the AUC scores for all cell lines

Figure 4 shows the AUC scores for the baseline method, the inverse signature method and the trans-disease method, with missing or completed data. Figure 4A and B shows the results of inhibitory and activatory target predictions, respectively. The SNP profiling method could not predict therapeutic targets by dividing them into inhibitory and activatory targets. Although it is technically impossible to determine whether the predicted targets are inhibitory or activatory by SNP profiling, they were regarded as true inhibitory or activatory target–disease pairs in this performance evaluation of inhibitory or activatory target predictions. Therefore, the accuracy of the SNP profiling method may have been overestimated. Nevertheless, the trans-disease method with completed data performed better than the SNP profiling method. The AUC scores for the trans-disease method with completed data (AUC = 0.630 for inhibitory target prediction; AUC = 0.651 for activatory target prediction) were higher than those for the SNP profiling method (AUC = 0.516 for inhibitory target prediction; AUC = 0.587 for activatory target prediction) for both inhibitory and activatory target predictions. These results indicate that the trans-disease method with completed data was the most effective for predicting the therapeutic targets of diseases. Finally, we evaluated the performance of therapeutic target predictions on a cell-by-cell basis. For nearly all cell lines, the trans-disease method with completed data performed best. Because of space limitation, the detailed results are presented in Supplementary Results.

### 3.3 Target repositioning can predict novel uses for existing therapeutic targets in different disease classes

We comprehensively predicted the unknown inhibitory targets and activatory targets of 79 diseases using the trans-disease method with completed data. All known target–disease associations were used as the learning dataset, and novel inhibitory and activatory targets were predicted. To predict the inhibitory targets, we used the gene knockdown signatures of 4345 proteins; to predict the activatory targets, we used the gene overexpression signatures of 3114 proteins. The knocked down and overexpressed genes included not only genes with known therapeutic indications for the diseases but also genes with no known therapeutic indications for any of the diseases. Specifically, 224 of the knocked down genes had known target–disease associations, whereas the remaining 4121 genes had no known target–disease associations. Thirty of the overexpressed genes had known target–disease associations, whereas the remaining 3084 genes had no known target–disease associations. The genes that had known therapeutic indications for the diseases were predicted to be repositioned from one disease to another, whereas the genes with no known therapeutic indications for any disease were predicted to be potential new therapeutic targets.

**Fig. 5.**
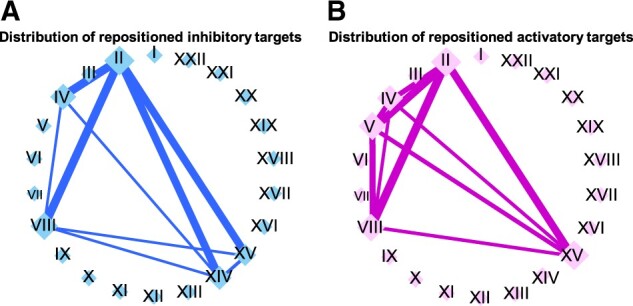
Distribution of therapeutic targets repositioned from the original disease classes to other disease classes. (**A**) Inhibitory targets. (**B**) Activatory targets. Nodes [indicated by blue and red diamonds in (A) and (B), respectively] represent ICD-11 disease chapters (shown with the chapter number). Edges [indicated by blue and pink lines for (A) and (B), respectively] represent potential correlations between diseases according to the new therapeutic effects of targets. The node size indicates the sum of the number of edges of each node. The edge width indicates the number of therapeutic targets repositioned between two disease chapters. I: certain infectious or parasitic diseases; II: neoplasms; III: diseases of the blood or blood-forming organs; IV: diseases of the immune system; V: endocrine, nutritional, or metabolic diseases; VI: mental, behavioral, or neurodevelopmental disorders; VII: sleep–wake disorders; VIII: diseases of the nervous system; IX: diseases of the visual system; X: diseases of the ear or mastoid process; XI: diseases of the circulatory system; XII: diseases of the respiratory system; XIII: diseases of the digestive system; XIV: diseases of the skin; XV: diseases of the musculoskeletal system or connective tissue; XVI: diseases of the genitourinary system; XVII: conditions related to sexual health; XVIII: pregnancy, childbirth, or the puerperium; XIX: certain conditions originating in the perinatal period; XX: developmental anomalies; XXI: symptoms, signs, or clinical findings, not elsewhere classified; and XXII: injury, poisoning or certain other consequences of external causes

To evaluate the predicted therapeutic targets in the framework of target repositioning, we examined their distribution based on the predicted indications. Figure 5A shows the distribution of inhibitory targets repositioned from the original disease classes to other disease classes, based on the predicted therapeutic indications of these targets. Sixty-two inhibitory targets were repositioned to other diseases in classes that differed from those of the original diseases. Diseases were classified according to the disease chapters in the International Statistical Classification of Diseases and Related Health Problems 11th version (ICD-11) (Reed *et al.*, 2019). The predictions indicated that a large number of inhibitory targets could possibly be repositioned from Chapter II (neoplasms) to Chapter IV (diseases of the immune system) and *vice versa*; furthermore, a possible repositioning from Chapter II (neoplasms) to Chapter VIII (diseases of the nervous system) and *vice versa* was indicated. These results suggest that the proposed large-scale prediction method can provide new therapeutic indications for a wide range of diseases. Supplementary Table S5 shows matrices that indicate the directions of repositioning between diseases.

Figure 5B shows the distribution of activatory targets repositioned from the original disease classes to other disease classes based on the predicted therapeutic indications of these targets. Forty-six activatory targets were repositioned to other diseases in classes that differed from those of the original diseases. As with the inhibitory target predictions, diseases were classified according to the ICD-11 disease chapters (Supplementary Table S6). A large number of activatory targets could possibly be repositioned from Chapter V (endocrine, nutritional or metabolic diseases) to Chapter II (neoplasms) and *vice versa*; additionally, possible activatory target repositioning from Chapter V (endocrine, nutritional or metabolic diseases) to Chapter VIII (diseases of the nervous system) and *vice versa* was indicated. Target repositioning can be performed using pathological knowledge, whereas repositioning to other diseases in classes that differ from those of the original disease cannot easily be predicted. Thus, the proposed approach has the potential to predict new therapeutic indications that are not easily identified from pathological knowledge alone.

### 3.4 Predicted network of inhibitory target–disease associations

We elaborated the validity of the newly predicted inhibitory target–disease associations. Fifty-two inhibitory targets were repositioned from the original disease to other diseases; these targets are listed in Supplementary Table S7.

**Fig. 6.**
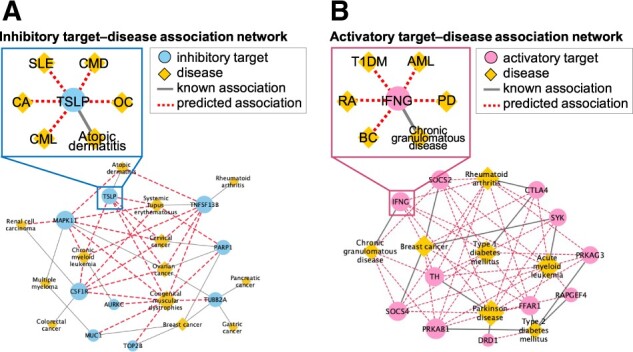
Small portions of newly predicted therapeutic target–disease association network. (**A**) Inhibitory target–disease association network and (**B**) activatory target–disease association network were predicted using the trans-disease method with completed data. Circles and diamonds denote therapeutic targets and diseases, respectively. Solid and dotted lines denote known and predicted associations, respectively. The upper squares in (A) and (B) denote the first nodes of TSLP and IFNG, respectively

Figure 6A shows a small portion of the network of inhibitory target–disease associations that was predicted using the trans-disease method. The associations are shown by focusing on targets that were repositioned from the original disease to other diseases. For example, the inhibition of TSLP, an inhibitory target of atopic dermatitis, was predicted to lead to therapeutic effects in cervical cancer (CC), ovarian cancer (OC), systemic lupus erythematosus (SLE), chronic myeloid leukemia (CML) and congenital muscular dystrophies. TSLP has previously been reported in the contexts of OC, in which the inhibition of TSLP was found to block metastasis (Ragonnaud et al., 2019; Xu et al., 2019; Chan et al., 2021); the TSLP signaling pathway is associated with SLE (Gorji et al., 2019); and TSLP was found to promote the development of CC (Zhang et al., 2017; Xie et al., 2015, 2013; Zhang and Jin, 2017). Thus, the inhibition of TSLP could produce therapeutic effects in these diseases. It appears that is possible to identify the therapeutic indications of existing inhibitory targets for various diseases using our proposed target repositioning method.

**Fig. 7.**
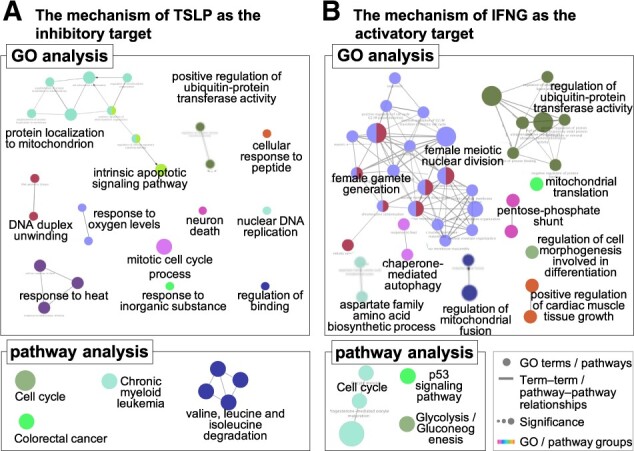
GO and KEGG pathway networks of (**A**) TSLP and (**B**) IFNG. (A) TSLP was predicted as the inhibitory target, and (B) IFNG was predicted as the activatory target. These analyses were performed based on the genes differentially expressed following TSLP knockdown and IFNG overexpression, respectively. The top panels show the GO analysis results for biological process terms. The bottom panels show the results of KEGG pathway analysis. The circles in the GO and KEGG pathway analysis represent GO terms and KEGG pathways, respectively. The edges denote term–term interactions and functional groups (GO groups) based on genes shared between the terms. The node colors represent GO groups. The node size represents the term significance; the biggest term of a group is the most significant, and it is highlighted on the network. These GO terms and pathways in the networks are shown in Supplementary Figure S7 and S8

To evaluate the value of the therapeutic indications of TSLP for predicted diseases, we then analyzed the GO biological process terms and KEGG pathways of the differentially expressed genes. Figure 7A shows the GO and pathway network for which TSLP is an inhibitory target. The GO gene annotation and KEGG pathway analysis of TSLP included 24 GO terms and 7 KEGG pathways. Significantly enriched GO groups included protein localization to mitochondrion (GO: 0070585; *P* = $6.05\;\times \;10^{-8}$), intrinsic apoptotic signaling pathway (GO: 0097193; *P* = $9.94\;\times \;10^{-7}$), response to heat (GO: 0009408; *P* = $1.40\;\times \;10^{-5}$) and response to oxygen levels (GO: 0070482; *P* = $3.38\;\times \;10^{-5}$); thus, strong associations were detected between TSLP and the proliferation and apoptosis of cancer cells. The CML pathway (KEGG: 05220) was also significantly enriched (*P* $=2.00\;\times \;10^{-2}$), suggesting an association with the therapeutic effects on CML. Overall, these results suggest that the inhibition of TSLP could be an effective treatment against OC, CC and CML. The results also provide further evidence that our proposed method of target repositioning can predict therapeutic indications of inhibitory targets.

### 3.5 Predicted network of activatory target–disease associations

We evaluated the validity of the newly predicted activatory target–disease associations; 46 activatory targets were repositioned from the original disease to other diseases (Supplementary Table S8).

Figure 6B shows a small portion of the network of activatory target–disease associations that was predicted by the trans-disease method. As in Figure 6A, the associations are shown by focusing on targets that were repositioned from the original disease to other diseases. As an example, the activation of IFNG, an activatory target of chronic granulomatous disease, was predicted to lead to therapeutic effects on rheumatoid arthritis (RA), Parkinson’s disease (PD), type I diabetes mellitus (T1DM), acute myeloid leukemia (AML) and breast cancer (BC). Previous research has found that the loss of IFNG reduces the therapeutic effects against AML (Matte-Martone *et al.*, 2017), that IFNG mitigates resistance to anticancer drugs in BC (Showalter *et al.*, 2019), that it is a key factor in the treatment of RA (He *et al.*, 2020) and that it is poorly expressed in T1DM (Sasaki *et al.*, 2004; Vaseghi *et al.*, 2016); thus, it is possible that the activation of IFNG would have therapeutic effects in these diseases. AML depresses the immune system, and both RA and T1DM are autoimmune diseases. IFNG is a cytokine that regulates the immune system. The predicted therapeutic indications of IFNG therefore appear to be promising.

We then performed GO analysis of biological processes and KEGG pathway analysis for IFNG based on differentially expressed genes. Figure 7B shows the GO and pathway network of IFNG as an activatory target; 38 GO terms and 5 KEGG pathways were associated with IFNG. Genes annotated to the biological process ‘regulation of ubiquitin–protein transferase activity’ (GO: 0051438; *P* $=8.23\;\times \;10^{-7}$) and its related GO group were significantly enriched (*P* $=2.57\;\times \;10^{-3}$). Ubiquitin plays an essential role in the removal of abnormal proteins from cells, and the loss of ubiquitin leads to neurodegenerative disorders. Thus, the activation of IFNG may lead to therapeutic effects in neurodegenerative disorders such as PD. Significant enrichment of GO groups related to female gamete generation (GO: 0007292; *P* $=2.82\;\times \;10^{-6}$) and female meiotic nuclear division (GO: 0007143; *P* $=3.99\;\times \;10^{-6}$) was also detected, as well as significant enrichment of the KEGG pathways ‘progesterone-mediated oocyte maturation’ (KEGG: 04914; *P* $=5.94\;\times \;10^{-3}$) and ‘oocyte meiosis’ (KEGG: 04114; *P* $=2.39\;\times \;10^{-3}$). Because progesterone is a hormone involved in the secretion of milk and the proliferation of BC cells, IFNG is expected to be an activatory therapeutic target for BC. The P53 signaling pathway (KEGG: 04115) was also significantly enriched (*P* $=1.60\;\times \;10^{-2}$). This pathway could potentially regulate the proliferation and apoptosis of cancer cells. Overall, these findings indicate that the activation of IFNG may lead to therapeutic effects in cancers such as BC and AML. As with the therapeutic indications of inhibitory targets, our proposed target repositioning method can potentially predict promising therapeutic indications of existing activatory targets.

### 3.6 Newly predicted therapeutic targets for orphan proteins without known target–disease associations

Finally, we evaluated the validity of the newly predicted therapeutic targets for orphan proteins without known target–disease associations. We evaluated the top 20 therapeutic target–disease pairs, which included completely new targets that have no known target–disease associations.

Table 1A lists the top 20 inhibitory targets for the applicable diseases from 4345 gene knockdown signatures and 32 diseases. Some examples, including ADA for adult T-cell leukemia (ATL), CD3D for T1DM and EPCAM for hepatitis C (HC), are known target–disease associations, indicating that our proposed method can accurately predict known therapeutic indications. Some proteins were predicted to be common inhibitory target candidates among different diseases. For example, TAF1B was predicted to be a common inhibitory therapeutic target for ATL, endometrial cancer, T1DM, HC, testicular cancer, tuberculosis and chronic lymphocytic leukemia. This wide distribution is thought to be due to the similarity of some of the causes and mechanisms of progression of these diseases. The same therapeutic target can be shared among different diseases if the mechanisms of disease pathogenesis are similar. These results both validate our inhibitory target prediction approach and identify new potential inhibitory targets that could potentially be used therapeutically for the treatment of their respective diseases.

**Table 1.** Inhibitory targets (A) and activatory targets (B) predicted using the trans-disease method with completed data

(A) Newly predicted inhibitory targets				(B) Newly predicted activatory targets			
Rank	Diseases	Inhibitory targets	Prediction scores	Rank	Diseases	Activatory targets	Prediction scores
1	Adult T-cell leukemia	TAF1B	1.000	1	Parkinson disease	OLIG3	1.000
2	Endometrial cancer	TAF1B	0.896	**2**	**Chronic myeloid leukemia**	**IFNAR2**	**0.999**
3	Adult T-cell leukemia	USP9X	0.871	**3**	**Multiple myeloma**	**IFNAR2**	**0.998**
4	Type I diabetes mellitus	TAF1B	0.868	4	Parkinson disease	NDUFC2	0.998
5	Hepatitis C	TAF1B	0.867	**5**	**Ovarian cancer**	**FBXW7**	**0.995**
6	Endometrial cancer	USP9X	0.832	**6**	**Rett syndrome**	**MECP2**	**0.992**
7	Testicular cancer	TAF1B	0.831	7	Type II diabetes mellitus	RPS6KA2	0.992
8	Type I diabetes mellitus	USP9X	0.826	**8**	**Type II diabetes mellitus**	**SIRT1**	**0.990**
9	Adult T-cell leukemia	TLK1	0.820	9	Parkinson disease	FOXO4	0.990
10	Hepatitis C	USP9X	0.819	10	Type II diabetes mellitus	PDIK1L	0.989
11	Tuberculosis	TAF1B	0.816	11	Type II diabetes mellitus	MEF2C	0.989
12	Adult T-cell leukemia	AATF	0.816	12	Type II diabetes mellitus	SETMAR	0.988
**13**	**Adult T-cell leukemia**	**ADA**	**0.814**	13	Parkinson disease	MYO3B	0.987
14	Hypercholesterolemia	TAF1B	0.813	**14**	**Colorectal cancer**	**TP53**	**0.987**
**15**	**Type I diabetes mellitus**	**CD3D**	**0.813**	**15**	**Type II diabetes mellitus**	**PPARG**	**0.986**
16	Chronic lymphocytic leukemia	TAF1B	0.810	**16**	**Crohn’s disease**	**IL10**	**0.984**
17	Type I diabetes mellitus	TLK1	0.809	**17**	**Inflammatory bowel disease**	**IL10**	**0.984**
18	Type I diabetes mellitus	ABCC1	0.807	18	Parkinson disease	BCR-ABL	0.983
**19**	**Hepatitis C**	**EPCAM**	**0.807**	19	Parkinson disease	ZNF384	0.983
20	Endometrial cancer	TLK1	0.807	20	Parkinson disease	FLJ25006	0.983

*Note*: These predicted targets included both existing therapeutic targets and entirely new therapeutic targets without any known target–disease associations. Inhibitory and activatory targets are listed. Prediction scores represent the therapeutic targetability of the indicated diseases. The top 100 target–disease associations can be found in Supplementary Tables S9 and S10. The bold values indicates known target–disease associations.

We also closely examined the top prediction for an inhibitory target–disease pair: TAF1B, an orphan protein without known target–disease associations, and ATL. We performed GO, KEGG pathway, and network topology analyses using the differentially expressed genes of the gene knockdown signature of TAF1B (Supplementary Fig. S9). From the GO analysis of TAF1B, 132 GO terms were detected. The significantly enriched GO groups included ‘myeloid leukocyte differentiation’ (GO: 0002573; *P* = $1.27\;\times \;10^{-7}$), ‘CD4-positive, alpha-beta T-cell activation’ (GO: 0035710; *P* =$\;1.58\;\times \;10^{-10}$), and ‘regulation of CD4-positive, alpha-beta T-cell activation’ (GO: 2000514; *P* =$\;4.31\;\times \;10^{-5}$). These annotations suggest that TAF1B is associated with the differentiation and regulation of CD4-positive T cells and other leukocytes. Because the oncogenic transformation of CD4-positive T cells is the cause of ATL, TAF1B inhibition is likely to be associated with ATL. In the KEGG pathway analysis, of 23 detected KEGG pathways, ‘human T-cell leukemia virus 1 (HTLV-1) infection’ (KEGG: 05166) was significantly enriched (*P* $=8.98\;\times \;10^{-4}$), suggesting that TAF1B could be associated with therapeutic effects on ATL, since HTLV-1 infection is the cause of ATL. Protein–protein interaction network analysis using the STRING database (Snel *et al.*, 2000) identified the SRC node as having the highest degree. SRC is a proto-oncogene that interacts with STAT3, one of the causal genes of ATL (Garcia *et al.*, 2001; Morichika *et al.*, 2019). SRC is also the target of the drug dasatinib, and its ingredients have been reported to have therapeutic effects on ATL (Kodama *et al.*, 2019). Thus, several lines of evidence suggest that TAF1B is a potential therapeutic target for ATL. Overall, these results imply that our proposed method can successfully predict therapeutic indications not only for proteins with known target–disease associations but also for orphan proteins without known target–disease associations.

Table 1B shows the top 20 activatory targets for the applicable diseases from 3114 gene overexpression signatures and 15 diseases. Some previously known target–disease associations detected were IFNAR2 for CML, IFNAR2 for multiple myeloma, SIRT1 for type II diabetes mellitus (T2DM), TP53 for colorectal cancer, PPARG for T2DM and IL10 for both Crohn’s disease and inflammatory bowel disease. We confirmed the validity of several prediction results using independent resources that were absent from the learning data. For example, the transcription factor MEF2C is not an existing activatory target with known target–disease associations, but it was predicted in this study to be an activatory target for T2DM. MEF2C has previously been reported to be poorly expressed in T2DM (Razeghi *et al.*, 2002; Yuasa *et al.*, 2015); thus, MEF2C is expected to produce therapeutic effects. These results demonstrate that our proposed method was valid for predicting both existing therapeutic targets and new therapeutic targets without known target–disease associations. These newly predicted activatory targets could potentially be developed for therapeutic purposes.

## 4 Discussion

In this study, we proposed target repositioning and developed two novel methods for predicting therapeutic targets of diseases, integrating disease-specific gene expression signatures and genetically perturbed gene expression signatures of target candidate proteins. The proposed trans-disease method, which takes into account similarities among diseases, enabled us to distinguish between inhibitory and activatory targets, and to predict the therapeutic targetability of proteins with known target–disease associations, and also those of orphan proteins without known associations. Our proposed method is expected to be useful for understanding the commonality of mechanisms among diseases, and for therapeutic target identification in drug discovery.

We attempted to predict therapeutic targets based on the assumption that there were inverse correlations between the diseases and their therapeutic targets; however, our results showed that prediction via the inverse signature method was challenging. The low prediction accuracy of the inverse signature method may be due to the lack of true therapeutic relationships between diseases and proteins because the inverse signature method is an unsupervised approach. The presence of multiple unknown therapeutic relationships between diseases and proteins would reduce the prediction accuracy. Therefore, the provision of additional relevant data will be important for further development.

In this study, the use of SNPs was employed as the baseline method. Since SNPs are naturally occurring phenomena that occur with high frequency, and are not patient specific, their use rarely leads to the identification of effective therapeutic targets. Thus, the SNP profiling method performs poorly at selecting effective therapeutic targets, because too many candidate genes for these targets are identified. The targets predicted by the SNP profiling method cannot be annotated as inhibitory or activatory, because the genetic data are static. Our proposed methods, however, can divide the predicted therapeutic targets into inhibitory or activatory, because they use gene expression data, which are dynamic, and reflect the transcriptomic responses of human cells. The determination of therapeutic targets as inhibitory or activatory is an advantage of our methods.

Although we analyzed 79 diseases in this study, our methods could be applied to many other diseases. The trans-disease method considers the similarities among diseases to overcome the scarcity of existing knowledge about the relationships among targets and diseases. The predictive performance of this method can be expected to improve as more diseases are targeted. The proposed method shares disease similarities among individual predictive models and therefore facilitates the repositioning of therapeutic targets. Consequently, our method has the potential to discover therapeutic targets that are effective for treating intractable diseases for which no effective therapeutic targets are currently available.

## Funding

This work was supported by the Ministry of Health, Labour and Welfare [21AC5001], Cabinet Office, Government of Japan, Public/Private R&D Investment Strategic Expansion Program (PRISM), JST AIP-PRISM [JPMJCR18Y5] and JSPS KAKENHI [20H05797 and 21H04915].

*Conflict of Interest*: none declared.

## Supplementary Material

btac240_Supplementary_Data
