# Supplementary material for: From drug repositioning to target repositioning: prediction of therapeutic targets using genetically perturbed transcriptomic signatures
Source: Bioinformatics. 2022 Jun 27;38(Suppl 1):i68–76. doi: 10.1093/bioinformatics/btac240 (PMC9235496; doi:10.1093/bioinformatics/btac240)
Supplement: btac240_Supplementary_Data [file btac240_supplementary_data.pdf]

Supplementary information for

From drug repositioning to target repositioning: prediction of therapeutic targets using genetically perturbed transcriptomic signatures

Satoko Namba, Michio Iwata, and Yoshihiro Yamanishi*

Department of Bioscience and Bioinformatics, Faculty of Computer Science and Systems Engineering, Kyushu Institute of Technology, Iizuka, Fukuoka 820-8502, Japan

*corresponding author: Yoshihiro Yamanishi (yamani@bio.kyutech.jp)

Table of contents

Supplementary Methods ………………………………………………………………………… 1

1. Alternative SNP profiling methods ………………………………………………… 1
2. A formulation of trans-disease method ……………………………………………… 3
3. Hierarchical clustering of perturbed genes ………………………………………… 5
4. Assessing the distribution of genetically perturbed genes ……………………… 5
5. GO and KEGG pathway analysis ………………………………………………… 5
6. Performance evaluation of data completion method ……………………………… 5

Supplementary Results ………………………………………………………………………… 7

1. Performance comparison of alternative SNP profiling methods ………………… 7
2. Performance evaluation of therapeutic target predictions on a cell-by-cell basis ……….. 8
3. Performance evaluation of data completion method ……………………………… 10

Supplementary Discussion ……………………………………………………………………… 11

1. Toward the appropriate selection of disease-related cell lines …………………… 11

Supplementary References …………………………………………………………………… 12

Supplementary Figures ………………………………………………………………………… 13

1. Performance comparison of alternative SNP profiling methods ………………… 13
2. Hierarchical clustering of genetically perturbed genes ………………………… 14
3. Classifications of genetically perturbed genes according to protein subfamily ……… 15
4. Performance evaluation for therapeutic target prediction using the inverse signature method ……………………………………………………………………………… 16
5. Performance evaluation for therapeutic target prediction using the trans-disease method ……………………………………………………………………………… 17
6. Performance evaluation among the inverse signature and trans-disease methods with missing and completed data …………………………………………………………… 18
7. Distribution of enriched GO terms and pathways in the GO and pathway analyses of TSLP ……………………………………………………………………………… 19
8. Distribution of enriched GO terms and pathways in the GO and pathway analyses of IFNG ……………………………………………………………………………… 20

9. GO, pathway, and PPI network results for TAF1B as an inhibitory target …………….. 22
10. Distribution of enriched GO terms in GO analysis of TAF1B predicted as the inhibitory target ……………………………………………………………………………...…. 23
11. Distribution of enriched pathways in the pathway analysis of TAF1B predicted as the inhibitory target …………………….……..………...…………………..…… 24

Supplementary Tables **……………………………………………………………………..…. 25**

1. Cell line list of genetically perturbed gene expression profiles ………………………… 25
2. Diseases that had at least one therapeutic target protein ………………………………... 26
3. Leaf nodes belonging to the clusters formed by the hierarchical clustering for knock-down genes ………………………………………………………………………………… 27
4. Leaf nodes belonging to the clusters formed by the hierarchical clustering for over-expressed genes ……………………………………………………………………..... 33
5. The distribution of inhibitory targets repositioned from the original disease class to other disease classes ……………………………………………………………………..… 38
6. The distribution of activatory targets repositioned from the original disease class to other disease classes …………………………………………………………………….. 49
7. Newly predicted 52 inhibitory targets repositioned from the original disease to other diseases ………………………………………………………………………………… 40
8. Newly predicted 46 activatory targets repositioned from the original disease to other diseases ………………………………………………………………………………… 42
9. Top 100 novel inhibitory target–disease pairs predicted using the trans-disease method with completed data …………………………………………………………………..… 43
10. Top 100 novel activatory target–disease pairs predicted using the trans-disease method with completed data …………………………………………………………………..… 46
11. Performance evaluation of data completion for the transcriptome data (knocked down genes, genes, and cell lines) ………………………………………….…………... 49
12. Performance evaluation of data completion for the transcriptome data (overexpressed genes, genes, and cell lines) ………………………………………….………... 50

Supplementary Methods

Alternative SNP profiling methods

We compared five different approaches using SNP information to select a baseline method for comparison with the proposed methods. We referred to these approaches as "Mean p-value method," "Gene based p-value method," "Mean NES method," "Min-max NES method," and "CADD method," respectively. The "Mean p-value method" used only SNPs within coding regions, while the other methods used SNPs within intergenic regions as well as coding regions. We obtained SNP data for the various diseases from the NHGRI-EBI Genome-wide Association Studies Catalog database (Buniello *et al.*, 2019), and constructed disease-specific SNP profiles. Using the SNP profiles, we predicted therapeutic targets.

In the "Mean p-value method," we predicted therapeutic targets focusing on the p-values of SNPs within coding regions to predict therapeutic targets that can be directly controlled by drugs. When a gene had multiple SNPs or was reported by multiple GWASs, we averaged the p-values for the gene. We constructed SNP profiles corresponding to the gold standard data by assigning the value 0 to genes with no SNP data. We used $-\log(P)$ values as predictive scores. Genes that had SNPs with significantly strong associations with disease were considered to be candidate therapeutic targets.

In the "Gene based p-value method," we predicted therapeutic targets using weighted Fisher's statistic (Hou, 2005) as a meta-analysis approach. MAGMA (de Leeuw *et al.*, 2015) was used as the GWAS meta-analysis tool. As input data, we used GWAS summary statistics for each disease obtained from the GWAS Catalog; MAGMA converted SNP-based p-values to gene-based p-values. For the gene-based p-value, we calculated -log(p-value) and used it as a prediction score. We predicted genes with high scores as candidates for therapeutic targets of each disease.

In the "Mean NES method" and "Min-max NES method," we predicted therapeutic targets using effect size to express strength of biological effect. We obtained normalized effect size (NES) for each SNP from "GTEx_Analysis_v8_eQTL" data obtained from Genotype-Tissue Expression (GTEx; Ardlie *et al.*, 2015). NESs of tissues associated with each disease were extracted. We examined two possible strategies for the NES-based prediction score: i) taking the average and ii) taking the min-max. In the former strategy, we averaged the NES values when multiple SNPs were present for each gene, and referred to it as "Mean NES method." In the latter strategy, we used the maximum and

minimum values of NESs for inhibitory target prediction and activatory target prediction, respectively, and referred to it as “Min-max NES method.” Genes with positive NES values should be inhibited for inhibitory target prediction, so we predicted genes with high NES values as candidate inhibitory targets. Genes with negative NES values were predicted to be activatory target candidates because genes with negative NES values should be activated.

In the “CADD method,” we predicted therapeutic targets based on functional annotation. We used FUMA (Watanabe *et al.*, 2017) to perform functional annotation on SNPs. GWAS summary statistics were used as input data for FUMA. SNPs were mapped onto genes based on their genomic location by positional mapping, which is the default function of FUMA. Since the therapeutic targets must be controlled by drugs, the mapped genes were protein-coding genes, which is the default setting of FUMA. The Combined Annotation Dependent Depletion (CADD) score (Kircher *et al.*, 2014; Rentzsch *et al.*, 2019), which indicates the effect of the mutation on functions, was used as a prediction score. We predicted genes with high CADD scores as candidate therapeutic targets of each disease.

A formulation of trans-disease method

We address the problem of therapeutic indications prediction by focusing on therapeutic targets. Note that there are a number of candidates for diseases, and different diseases may have common characteristics in terms of molecular mechanisms. The same therapeutic targets are sometimes used for multiple diseases. Thus, we propose formulating the problem in the framework of supervised multiple label prediction.

Suppose that there are M diseases and we are given N targets. We consider predicting which diseases would be treated by a target, that is, the i-th target. Each target is represented by a d-dimensional feature vector as $\boldsymbol{x}_i$ in this study, where $\boldsymbol{x}_i$ was obtained by averaging the multiple signatures from different cell lines.

We constructed a learning set of target–disease pairs that are pairs given in target–disease associations (see the Methods section for more details). There are M candidates for diseases, and each target in the learning set is assigned a binary class label representing the m-th disease ($m = 1, 2, \cdots, M$). Let $y_{m,i} \in \{0, 1\}$ be the class label for the m-th disease assigned to the i-th target, where $y_{m,i} = 1$ means that i-th target is used for the m-th disease, and $y_{m,i} = 0$ means that the i-th target is not used for the m-th disease.

We construct a predictive model to predict whether the i-th target would be used for the m-th disease ($m = 1, 2, \cdots, M$). Linear models are a useful tool to analyze extremely high-dimensional data for both prediction and feature extraction tasks. Thus, we adopt a linear function defined as $f_m = \boldsymbol{w}_m^{\mathrm{T}}\boldsymbol{x}$, where $\boldsymbol{w}_m$ is a d-dimensional weight vector for the m-th disease. We represent a set of M model weights by a $d \times M$ matrix defined as $\mathbf{W} := [\boldsymbol{w}_1, \boldsymbol{w}_2, \cdots, \boldsymbol{w}_M]$ and estimate the weight matrix $\mathbf{W}$ by minimizing an objective function based on the learning set.

To overcome the scarcity of existing knowledge concerning relationships between targets and diseases, we propose learning individual predictive models $f_1, f_2, \cdots, f_M$ jointly, sharing information across M diseases.

We attempt to estimate all of the weight vectors $\boldsymbol{w}_1, \boldsymbol{w}_2, \cdots, \boldsymbol{w}_M$ jointly in the models by minimizing the logistic loss as follows:

$$\boldsymbol{R}(\mathbf{W}) = \sum_{\boldsymbol{m}=\mathbf{1}}^{\boldsymbol{M}} \sum_{\boldsymbol{i}=\mathbf{1}}^{\boldsymbol{N}} \mathbf{log}\left(\mathbf{1} + \mathbf{exp}\left(-\boldsymbol{y}_{\boldsymbol{m},\boldsymbol{i}} \boldsymbol{w}_{\boldsymbol{m}}^{\mathbf{T}} \boldsymbol{x}_{\boldsymbol{i}}\right)\right). \quad (1)$$

We introduce a regularization term $\Omega(\mathbf{W})$ to the loss function in order to enhance the generalization properties. Thus, the optimization problem is written as follows:

$$\min_{\mathbf{W}} \boldsymbol{R}(\mathbf{W}) + \boldsymbol{\Omega}(\mathbf{W}). \tag{2}$$

Here we introduce two regularization terms. First, we use a standard ridge regularization term to avoid the over-fitting problem, which is defined as

$$\boldsymbol{\Omega}_r := \frac{1}{2} \boldsymbol{Tr}(\mathbf{W}\mathbf{W}^{\mathrm{T}}). \tag{3}$$

Second, we design another regularization term reflecting the similarities among diseases. In this study we evaluate the similarity among diseases using the cosine similarity and construct an $M \times M$ similarity matrix $\mathbf{S}$ for diseases in which each element $S_{i,j}$ is a similarity score between the i-th and j-th diseases. Then, we introduce the following regularization term:

$$\boldsymbol{\Omega}_s(\mathbf{W}) := \frac{1}{4} \sum_{l=1}^{M} \sum_{m=1}^{M} S_{l,m} \left\| \frac{\boldsymbol{w}_l}{\sqrt{\mathbf{K}_{l,l}}} - \frac{\boldsymbol{w}_m}{\sqrt{\mathbf{K}_{m,m}}} \right\| = \frac{1}{2} \boldsymbol{Tr}(\mathbf{W}\mathbf{L}_s\mathbf{W}^{\mathrm{T}}), \tag{4}$$

where $\|\cdot\|$ is the Euclidean norm, $\mathbf{K}$ is a diagonal matrix defined as $\mathrm{K}_{l,l} := \sum_{m=1}^{M} S_{l,m}$, and $\mathbf{L}_s$ is a symmetric normalized Laplacian defined as $\mathbf{K}^{-1/2}(\mathbf{K} - \mathbf{S})\mathbf{K}^{-1/2}$. The regularization term $\Omega_s(\mathbf{W})$ has the effect of bringing the weight vectors $\boldsymbol{w}_i$ and $\boldsymbol{w}_j$ close to each other if $S_{l,m}$ is high.

Finally, we introduce the following regularization term in the optimization problem (2):

$$\boldsymbol{\Omega}(\mathbf{W}) := \boldsymbol{\lambda}_s \boldsymbol{\Omega}_s(\mathbf{W}) + \boldsymbol{\lambda}_r \boldsymbol{\Omega}_r(\mathbf{W}), \tag{5}$$

where $\lambda_s \geq 0$ and $\lambda_r \geq 0$ are hyper-parameters to control the strength of the regularization terms Ω_S and Ω_r, respectively.

A grid search was performed to determine the optimal set of regularization parameters λ_s and λ_r. We prepared a set of candidate values {0.001, 0.01, 0.1, 1, 10, 100, 1000} for λ_s and λ_r in advance. We calculated the AUC scores by performing a five-fold cross-validation experiment. We selected parameter values that achieved the highest AUC score. We used the parameters to predict applicable diseases for the therapeutic targets.

Hierarchical clustering of perturbed genes

To examine the features of genetically perturbed gene expression signatures, we performed hierarchical clustering of both the genetically perturbed genes and the L1000 genes. Hierarchical clustering was performed using the Python library SciPy (v 1.4.1, https://www.scipy.org/) with the following parameters: method = "ward" and metric = "euclidean."

Assessing the distribution of genetically perturbed genes

To evaluate whether the set of genetically perturbed genes contained an adequate number of candidate therapeutic targets, we examined the distribution of these genes according to their protein family, biological processes, and biological pathways. Specifically, we classified these genes based on the protein families and subfamilies provided by PANTHER (Mi *et al.*, 2013; Thomas *et al.*, 2003), their associated GO biological process terms, and their linked KEGG pathways in the KEGG database (Kanehisa *et al.*, 2002). "Global pathways" were excluded.

GO and KEGG pathway analysis

GO analysis is usually used to annotate genes and gene products according to their characteristic biological functions (Ashburner *et al.*, 2000), whereas KEGG pathway analysis assigns genes to functional pathways (Kanehisa *et al.*, 2002).

In this study, we performed GO and KEGG pathway analysis to analyze the mechanisms of the therapeutic indications of predicted inhibitory and activated targets. We extracted differentially expressed genes from the gene expression signatures after perturbation of these targets, and these genes were used for further analyses. A Cytoscape plug-in, ClueGO, was used to identify over-represented GO terms and KEGG pathways(Bindea *et al.*, 2009).

Performance evaluation of data completion method

We evaluated the performance of the tensor decomposition algorithm (Yuan *et al.*, 2017) used in this study in terms of the imputation accuracy. For comparison, we compared the performance of the algorithm with the CANDECOMP/PARAFAC (CP) decomposition (Acar *et al.*, 2010). To evaluate

the performance of the imputation, we randomly added artificial missing values to the observed data and tested whether the tensor imputation algorithms could correctly recover these values.

Supplementary Results

Performance comparison of alternative SNP profiling methods

We compared five different approaches using SNP information to select a baseline method for comparison with the proposed methods; “Mean p-value method,” “Gene based p-value method,” “Mean NES method,” “Min-max NES method,” and “CADD method” (see Supplementary Methods section).

Supplementary Fig. S1 compares the predictive performances of these different SNP profiling methods. There was little difference between these methods when predicting inhibitory targets, as shown in Supplementary Fig. S1 (A). However, in the case of activatory target prediction, the prediction accuracy of the Mean p-value method was slightly higher than those of other methods, as shown in Supplementary Fig. S1 (B). The Mean p-value method uses only SNPs in the coding region, while the other methods also use SNPs in the intergenic region, indicating that the focus on SNPs within coding regions is slightly more accurate in terms of therapeutic target prediction. Since the mean p-value method uses the averaged p-values of SNPs, while the Gene based p-value method uses gene-based p-values converted by meta-analysis with MAGM, suggesting that the focus on using the averaged p-values is slightly more accurate in terms of therapeutic target prediction. The mean p-value method uses the significance of association, the Mean NES method and Min-max NES method use the NES score, which represents the strength of the biological effect, and the CADD method, which reflect the effect of the mutation on functions, suggesting that the using the significance of association is slightly more accurate in terms of therapeutic target prediction. These results suggest that the using the significance of association (averaged p-values) is slightly more accurate in terms of therapeutic target prediction.

Based on these results, we decided to use the slightly more accurate Mean p-value method for comparison with the proposed methods. In the main text, this method is referred to as the SNP profiling method.

Performance evaluation of therapeutic target predictions on a cell-by-cell basis

We compared predictive performance when using completed data vs. missing data. As shown in Fig. 4, the completion of genetically perturbed gene expression signature data improved the predictive performance of the methods, particularly when using the trans-disease method ($P = 5.01 \times 10^{-4}$ for inhibitory target–disease pairs; $P = 1.09 \times 10^{-2}$ for activatory target–disease pairs; Wilcoxon signed-rank tests). In all cases, the trans-disease method with completed data outperformed the inverse signature method with missing data ($P = 1.47 \times 10^{-4}$ for inhibitory target–disease pairs; $P = 2.53 \times 10^{-3}$ for activatory target–disease pairs). These results indicate that the trans-disease method with completed data is the most effective method for predicting therapeutic targets for diseases.

We evaluated the ability of the inverse signature method to predict therapeutic targets on a cell-by-cell basis. Supplementary Fig. S4 shows a comparison of the AUC scores for each cell line and the associated missing rate in the gene expression signatures for each cell line. Supplementary Fig. S4 (A) and (B) show evaluations of the inhibitory target prediction and activatory target prediction performances, respectively. For nearly all cell lines, the AUC scores for both the inhibitory and activatory target predictions were about 0.5, which shows that the predictions obtained using the inverse signature method were random inferences. This issue may have arisen because the inverse correlation method involves unsupervised learning. Because the number of known therapeutic target–disease associations is very limited, the accuracy of this method may be underestimated.

When using the trans-disease method, we evaluated the performance of therapeutic target predictions by performing five-fold cross-validation experiments. We calculated the AUC scores using the method described int the Results section. Supplementary Fig. S5 shows a comparison of the AUC scores for each cell line and the associated missing rate in the gene expression signatures for the same cell lines. Supplementary Fig. S5 (A) shows a performance evaluation for the inhibitory target prediction. For nearly all cell lines, inhibitory target predictions by the trans-disease method were superior to the predictions produced using the inverse signature method. In cell lines with high missing rates, the performance of predictions was poor. Supplementary Fig. S5 (B) shows a performance evaluation for the activatory target prediction. Performances differed depending on the cell line, which suggests that activatory target prediction by the trans-disease method performed well

in certain cell lines. Overall, these results indicate that the trans-disease method outperformed the inverse signature method when predicting therapeutic targets. However, in cell lines with high rates of missing data, the trans-disease method was not effective for inhibitory target predictions in particular.

Finally, we compared the predictive performances of the methods for each cell line. Supplementary Fig. S6 (A) shows a comparison of the AUC scores of inhibitory target prediction for each cell line as well as the associated missing rate in the gene expression signatures for each cell line. For nearly all cell lines, the use of gene expression signatures with completed data was more effective than the use of gene expression signatures with missing data; this finding was especially valid when using the trans-disease method. In cell lines with high rates of missing data, the usefulness of gene expression signatures with completed data was clearly shown. Notably, genetically perturbed gene expression signatures were largely unobserved (96.4% unobserved) in several cell lines, including SHSY5Y, SKL, HEK293T, and NCIH716, indicating that such cell lines included few perturbed genes. Supplementary Fig. S6 (B) shows a comparison of the AUC scores of activatory target prediction for each cell line as well as the associated missing rate in the gene expression signatures for each cell line. As with inhibitory target predictions, for nearly all cell lines, the use of gene expression signatures with completed data produced better results than those produced using gene expression signatures with missing data, particularly when using the trans-disease method. Thus, predictive performance can apparently be improved by imputing many missing values of genetically perturbed gene expression signatures.

Performance evaluation of data completion method

Supplementary Table S11 shows the result for the performance evaluation of the data completion for a third-ordered gene expression dataset comprising 4,345 knocked down genes, 978 genes and 17 cell lines, where the dataset contains artificial missing values. Similarity, Supplementary Table S12 shows the result for the performance evaluation of the data completion for a third-ordered gene expression dataset comprising 3,114 overexpressed genes, 978 genes and 10 cell lines, where the dataset contains artificial missing values. In these evaluations, the tensor decomposition algorithm used in this study worked better than the algorithm with the CP decomposition. These results suggest that the tensor decomposition algorithm can work well for data completion.

Supplementary Discussion

Toward the appropriate selection of disease-related cell lines

We evaluated the performance of our proposed methods on a cell-by-cell basis (Supplementary Results and Figs. S4–S6). It would be ideal to use only cellular data associated with disease organs; unfortunately, such data associated disease organs is not always available. In fact, the cellular data in this study was limited to cell lines because the cellular data for all diseases studied here was not available. However, we predicted new therapeutic target–disease associations using the proposed method based on the averaged cell line data. Note that our proposed method is applicable to new predictions on a cell-by-cell basis when genetically perturbed gene expression profiles are available for all cell lines that correspond to diseased organs. Accumulation of more data should solve this problem in the future.

Supplementary References

Acar,E. *et al.* (2010) Scalable tensor factorizations for incomplete data ☆.

Ardlie,K.G. *et al.* (2015) Human genomics. The Genotype-Tissue Expression (GTEx) pilot analysis: multitissue gene regulation in humans. *Science*, **348**, 648–660.

Ashburner,M. *et al.* (2000) Gene ontology: Tool for the unification of biology. *Nat. Genet.*, **25**, 25–29.

Bindea,G. *et al.* (2009) ClueGO: A Cytoscape plug-in to decipher functionally grouped gene ontology and pathway annotation networks. *Bioinformatics*, **25**, 1091–1093.

Buniello,A. *et al.* (2019) The NHGRI-EBI GWAS Catalog of published genome-wide association studies, targeted arrays and summary statistics 2019. *Nucleic Acids Res.*, **47**, D1005–D1012.

Hou,C.D. (2005) A simple approximation for the distribution of the weighted combination of non-independent or independent probabilities. *Stat. Probab. Lett.*, **73**, 179–187.

Kanehisa,M. *et al.* (2002) Thed KEGG databases at GenomeNet. *Nucleic Acids Res.*, **30**, 42–46.

Kircher,M. *et al.* (2014) A general framework for estimating the relative pathogenicity of human genetic variants. *Nat. Genet.*, **46**, 310–315.

de Leeuw,C.A. *et al.* (2015) MAGMA: generalized gene-set analysis of GWAS data. *PLoS Comput. Biol.*, **11**.

Mi,H. *et al.* (2013) PANTHER in 2013: Modeling the evolution of gene function, and other gene attributes, in the context of phylogenetic trees. *Nucleic Acids Res.*, **41**.

Rentzsch,P. *et al.* (2019) CADD: predicting the deleteriousness of variants throughout the human genome. *Nucleic Acids Res.*, **47**, D886–D894.

Thomas,P.D. *et al.* (2003) PANTHER: A library of protein families and subfamilies indexed by function. *Genome Res.*, **13**, 2129–2141.

Watanabe,K. *et al.* (2017) Functional mapping and annotation of genetic associations with FUMA. *Nat. Commun.*, **8**.

Yuan,L. *et al.* (2017) Completion of high order tensor data with missing entries via tensor-train decomposition. In, *Lecture Notes in Computer Science (including subseries Lecture Notes in Artificial Intelligence and Lecture Notes in Bioinformatics)*. Springer Verlag, pp. 222–229.

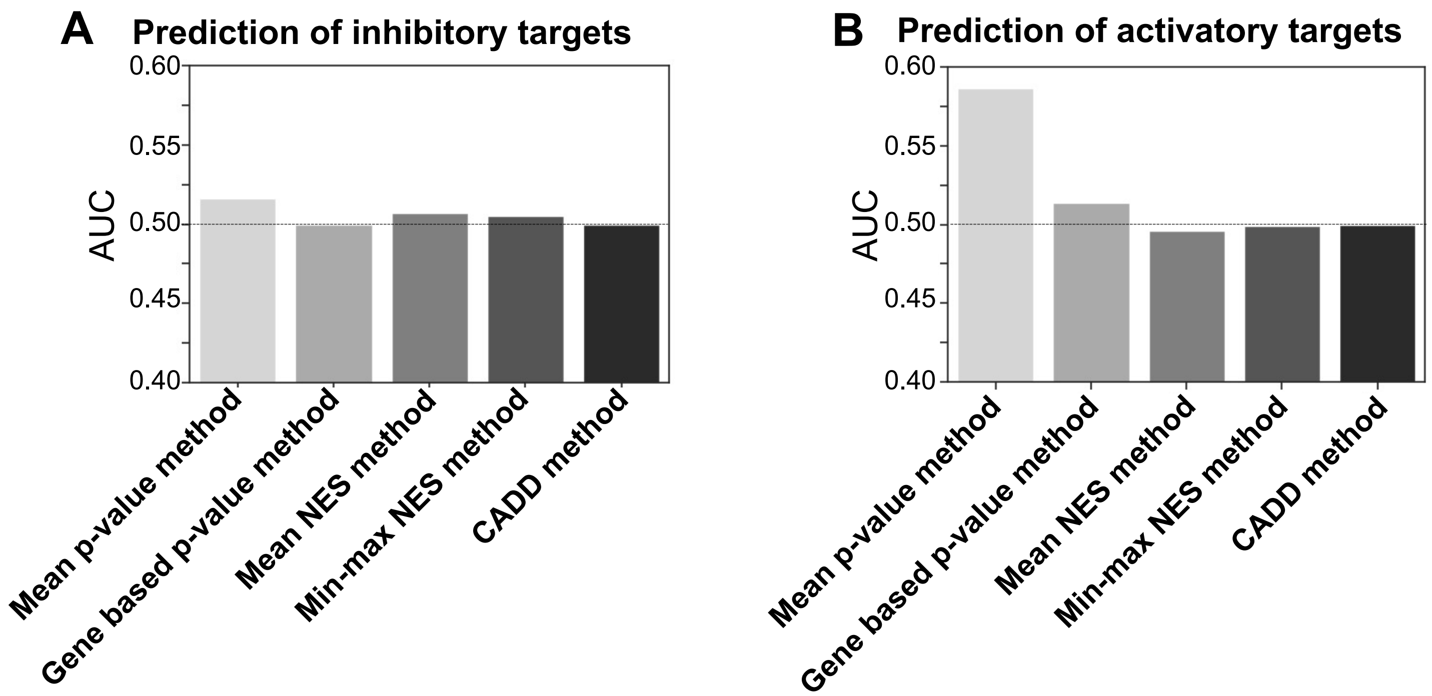

Supplementary Figure S1. Performance comparison of alternative SNP profiling methods. (A) Performance comparison on inhibitory target prediction. (B) Performance comparison on activatory target prediction. Mean p-value method used only SNPs within coding regions, while the other methods used SNPs within intergenic regions as well as coding regions. Mean p-value method is based on the significance of association (*p*-values), and corresponds to Fig. 4; Gene based p-value method is based on meta-analysis approach with MAGMA; Mean NES method is based on the normalized effect size (NES) with the average operation; Min-max NES method is based on NES with the min/max operation; CADD method is based on functional annotation with FUMA.

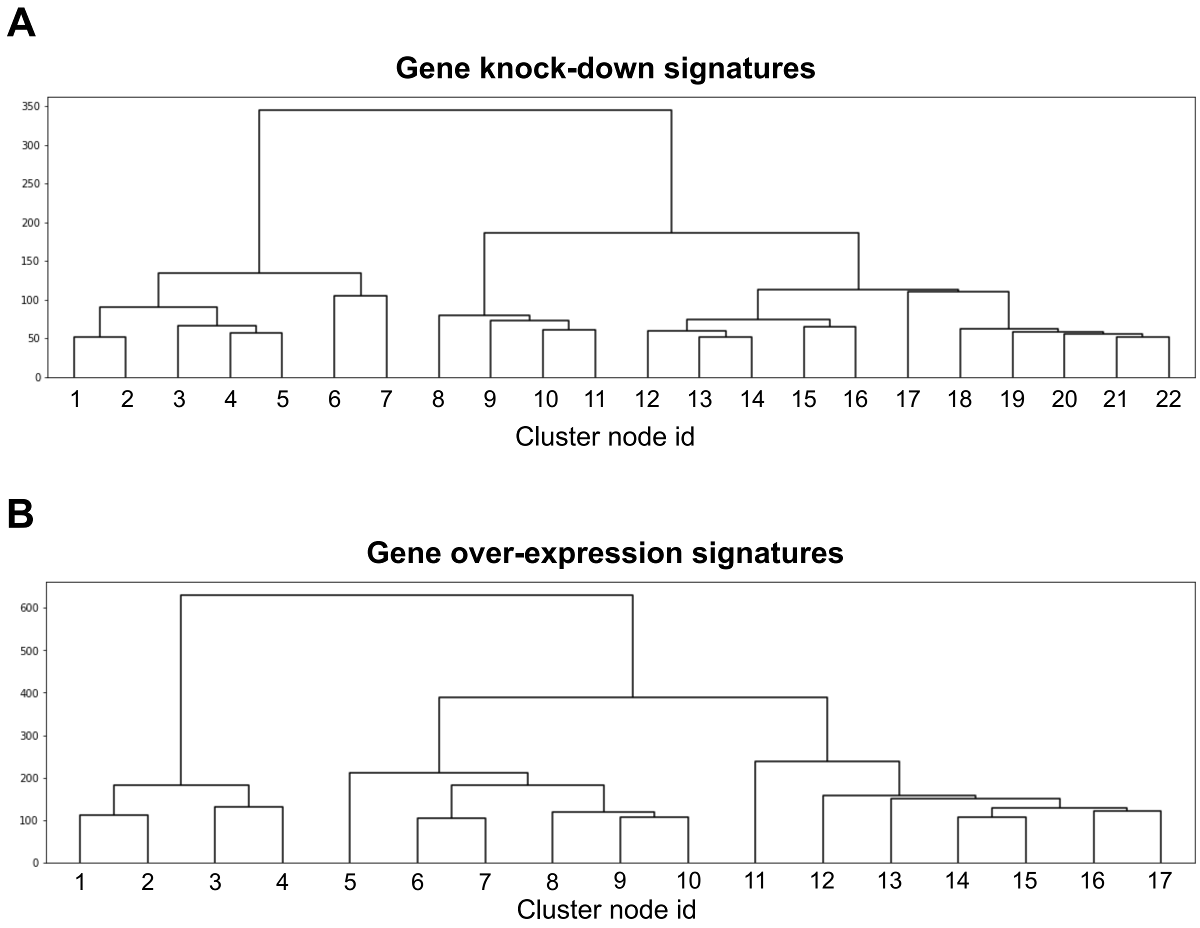

Supplementary Figure S2. Hierarchical clustering of (A) gene knock-down and (B) gene over-expression signatures. These dendrograms represent the results of hierarchical clustering for 4,345 knocked down and 3,114 overexpressed genes, respectively. Cluster node ids represent identifiers of clusters. Leaf nodes belonging to each cluster node id are shown in Supplementary Tables S3 and S4.

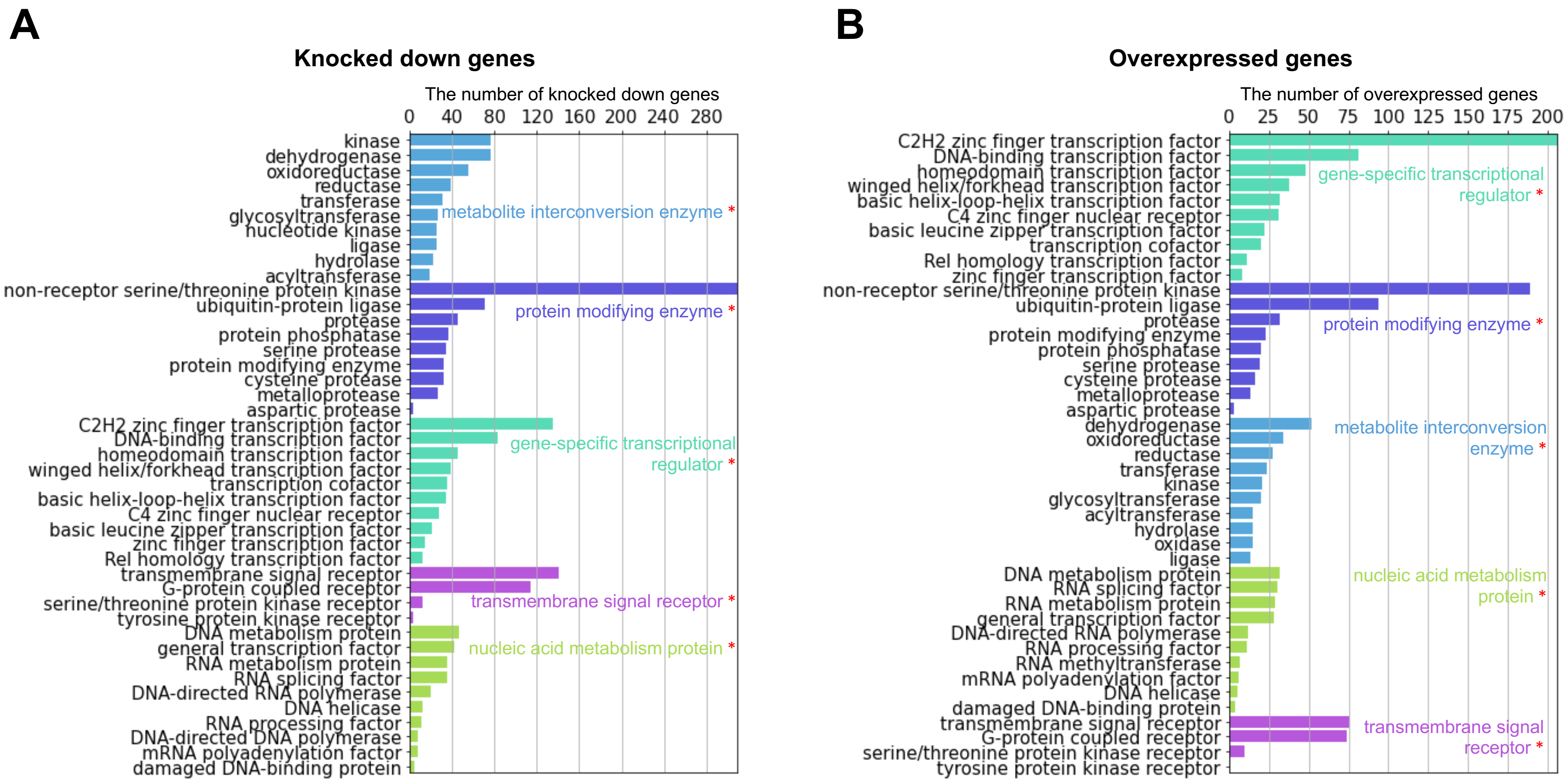

Supplementary Figure S3. Classifications of (A) knocked down genes and (B) overexpressed genes according to protein subfamily. These protein subfamilies belonged to the top 5 protein families in the top panel of the Fig. 3, and these protein subfamilies were obtained from the Protein Analysis Through Evolutionary Relationships (PANTHER; Mi *et al.*, 2013; Thomas *et al.*, 2003). Since the number of protein subfamilies was too large, the top 10 subfamilies in each protein family were displayed.

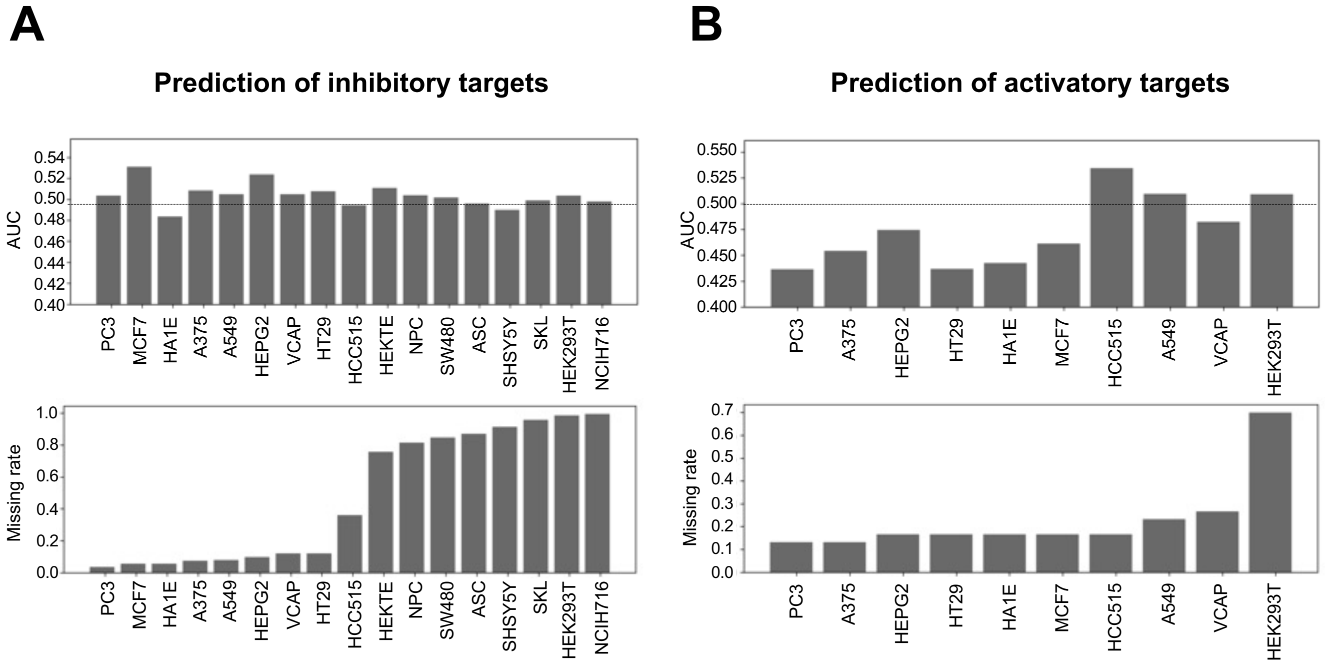

Supplementary Figure S4. Performance evaluation for therapeutic target prediction, using the inverse signature method. Performance evaluation for (A) inhibitory target prediction and (B) activatory target prediction. The top panel shows the area under the ROC curve score calculated using all prediction scores for all target–disease pairs. The bottom panel shows the rate of missing data in each cell line. Cell lines are listed in increasing order of the missing rate.

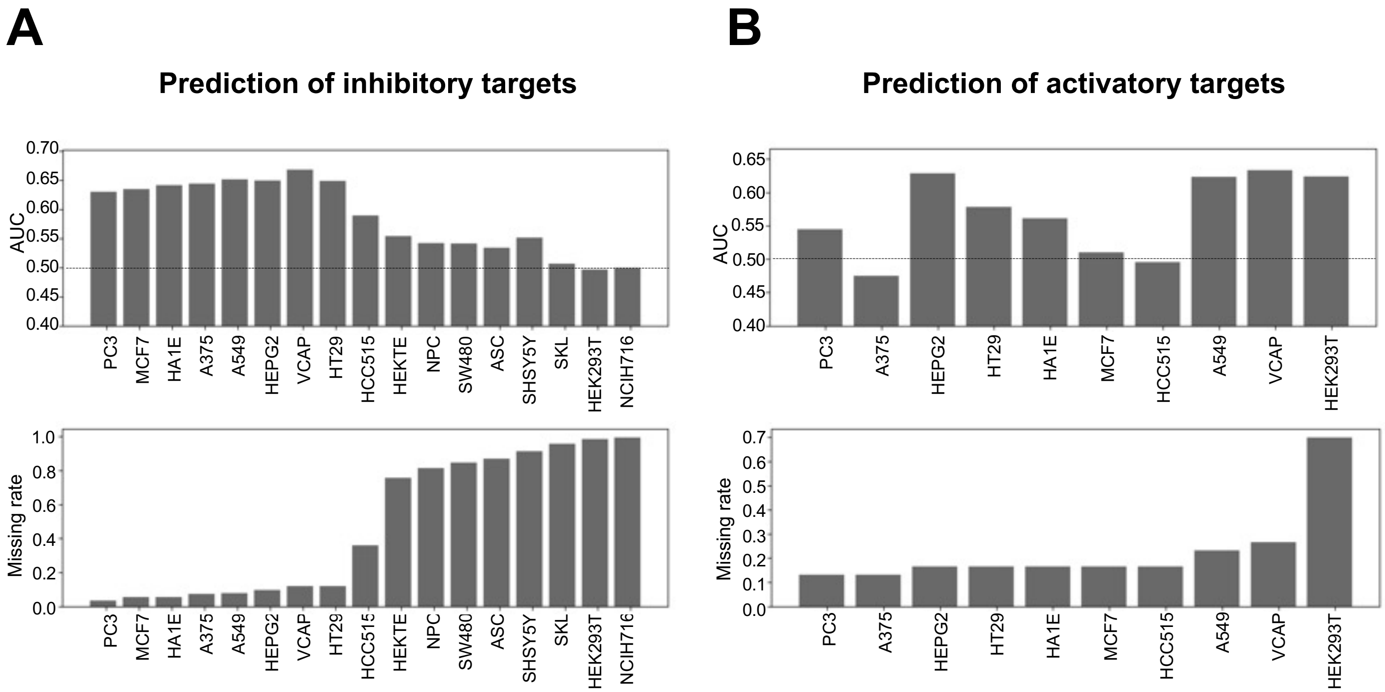

Supplementary Figure S5. Performance evaluation for therapeutic target prediction using the trans-disease method. Performance evaluation for (A) inhibitory target prediction and (B) activatory target prediction. The top panel shows the AUC score calculated using all prediction scores for all target–disease pairs. The bottom panel shows the rate of missing data in each cell line. Cell lines are listed in increasing order of missing rate.

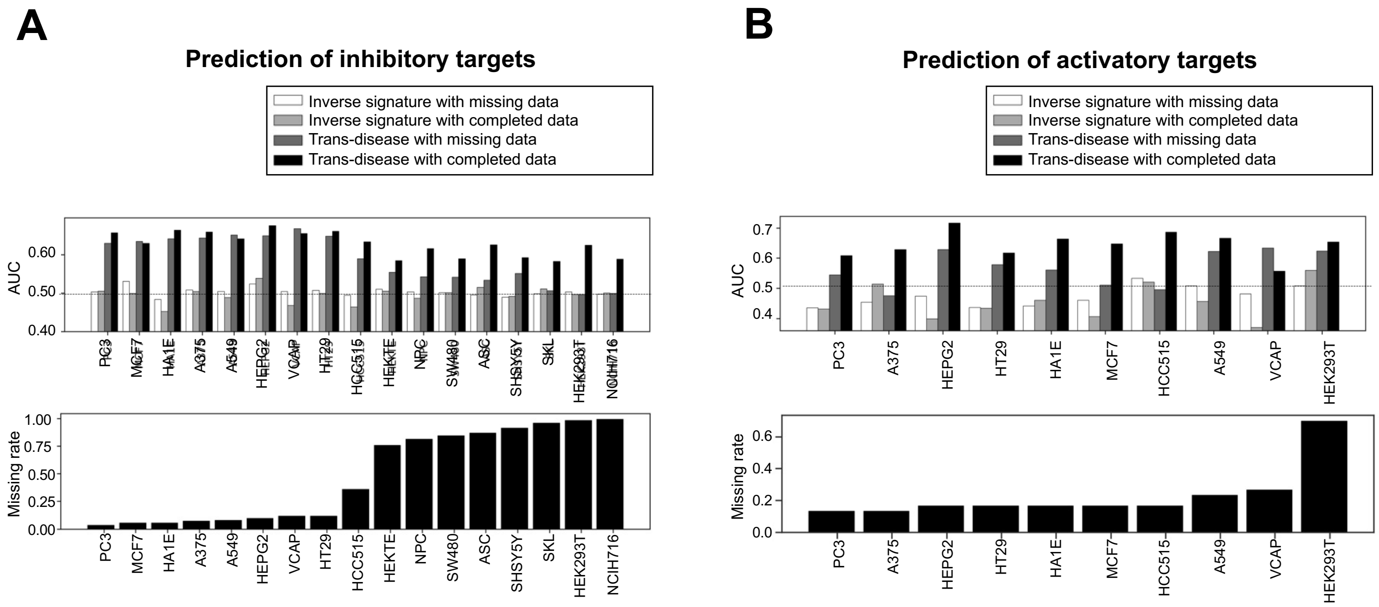

Supplementary Figure S6. Performance comparison among the inverse signature and trans-disease methods with missing and completed data. (A) Performance comparison on inhibitory target prediction. (B) Performance comparison on activatory target prediction. The top panel shows the AUC score calculated using all prediction scores for all target–disease pairs. The bottom panel shows the rate of missing data in each cell line. Cell lines are listed in increasing order of missing rate.

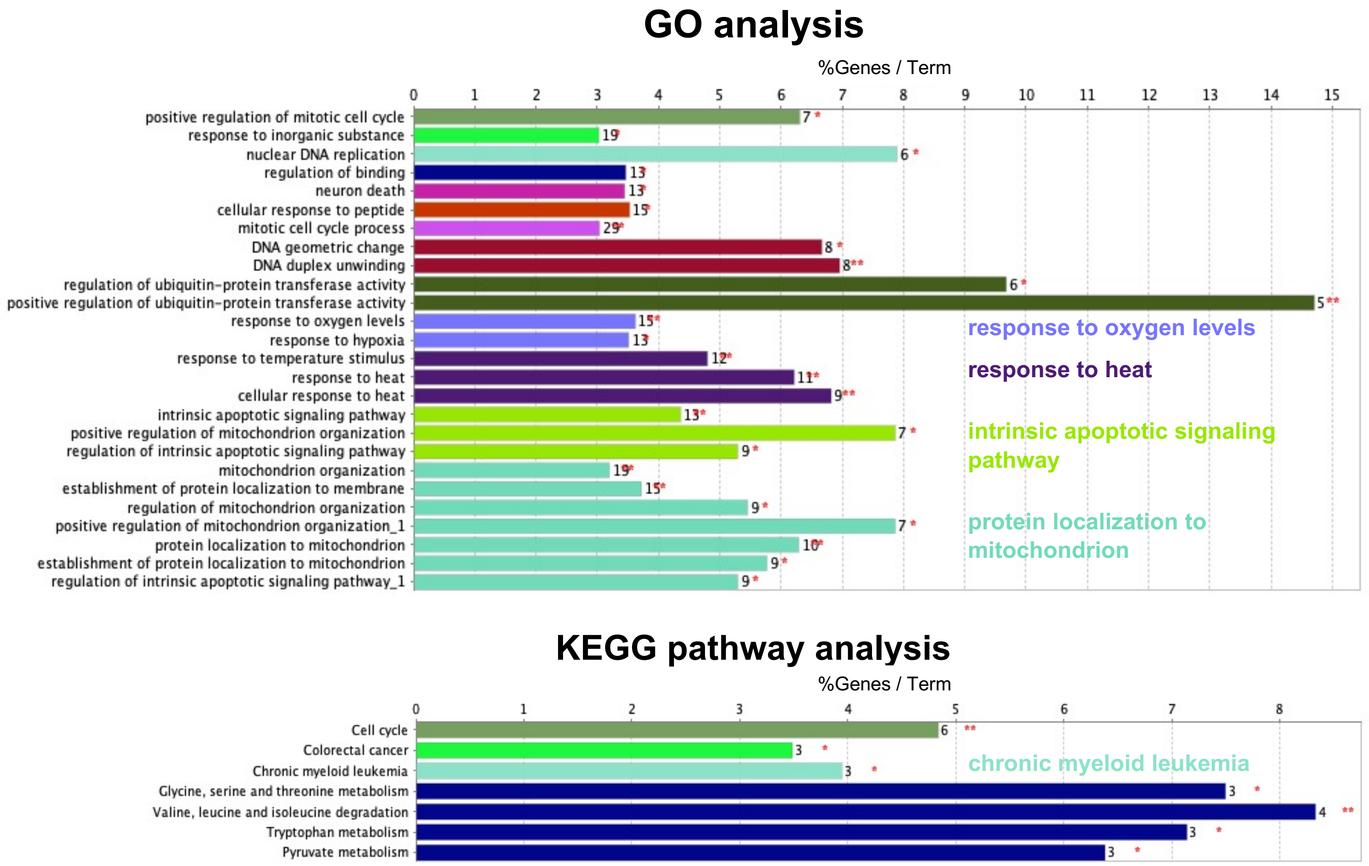

Supplementary Figure S7. Distribution of enriched GO terms and pathways in the GO and pathway analyses of TSLP. The top panel shows the result of GO analysis of the biological process. The bottom panel shows the result of KEGG pathway analysis. These analyses were performed based on differentially expressed genes of gene expression signatures following TSLP knock-down. The x axis represents the ratio of associated genes to all genes in the term or pathway. The numbers next to the bars represent the number of genes associated with the term or pathway. The color represents the GO or pathway groups. Asterisks indicate the significance level as follows: $* \, p < 0.05$, $** \, P < 0.01$. The functionally grouped network of these GO terms or pathways are shown in Fig. 7 (A).

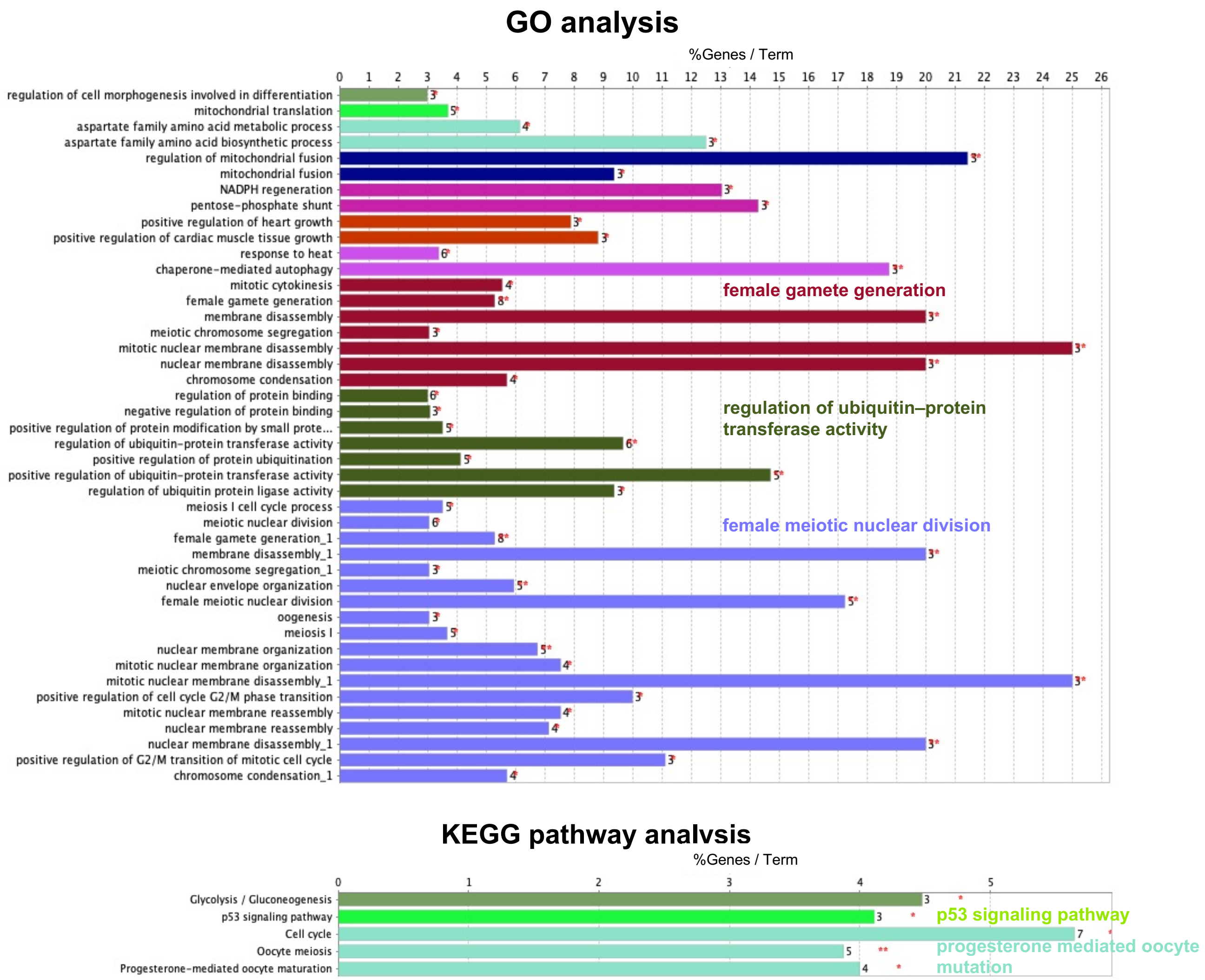

Supplementary Figure S8. Distribution of enriched GO terms and pathways in the GO and pathway analyses of IFNG. The top panel shows the result of GO analysis of the biological process. The bottom panel shows the result of KEGG pathway analysis. These analyses

were performed based on differentially expressed genes of gene expression signatures following IFNG over-expression. The x axis represents the ratio of associated genes to all genes in the term or pathway. The numbers next to the bars represent the number of genes associated with the term or pathway. The color represents the GO or pathway groups. Asterisks indicate the significance level as follows: $* p < 0.05$, $** P < 0.01$. The functionally grouped network of these GO terms or pathways are shown in Fig. 7 (B).

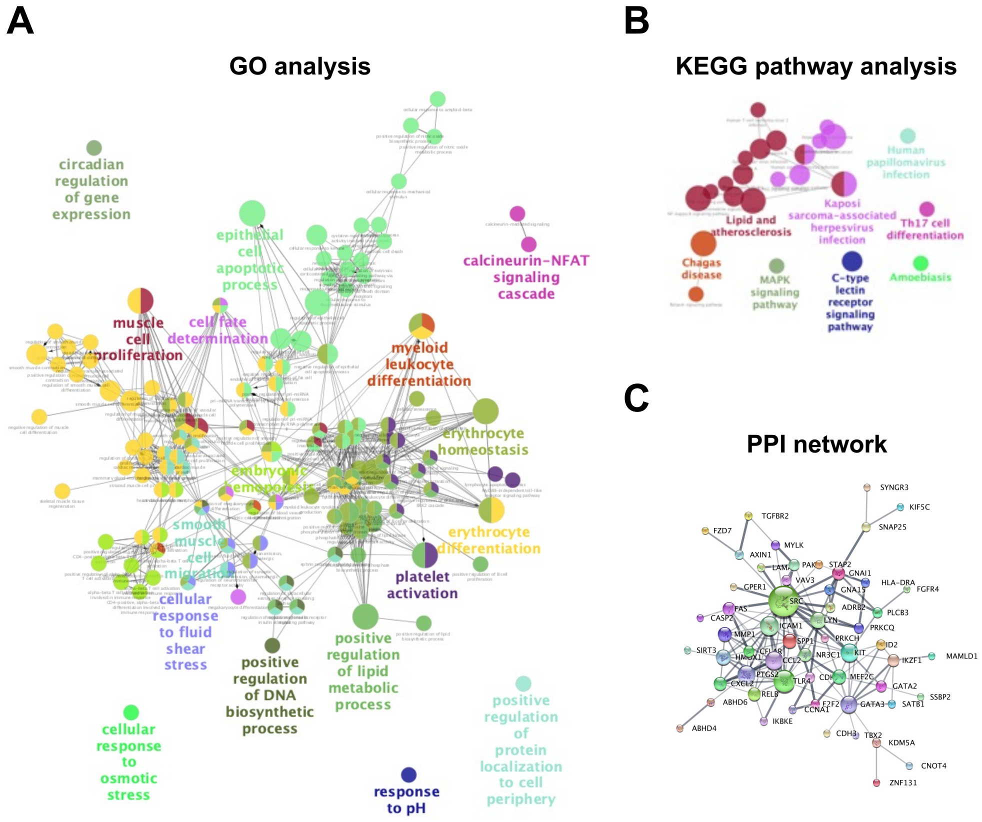

Supplementary Figure S9. (A) GO, (B) pathway, and (C) PPI network results for TAF1B as an inhibitory target. These analyses were performed using the genes differentially expressed following TAF1B knockdown. (A) The analysis of GO biological process terms and (B) KEGG pathway analysis, with circles representing GO terms and KEGG pathways, respectively. The edges denote term–term interactions and functional groups (GO groups) based on the genes shared between the terms. The node color represents the GO group. The node size represents the term significance; the biggest term from a group is the most significant and is highlighted. The GO terms and pathways in these networks are shown in Supplementary Figs. S10 and S11. (C) PPI network showing the protein (gene)–protein (gene) interaction network altered in response to TAF1B knockdown, as well as the results of network topology analysis. The circles in the network represent proteins. The edges denote protein–protein interactions. The node size represents degree. The edge size represents interaction confidence defined according to STRING.

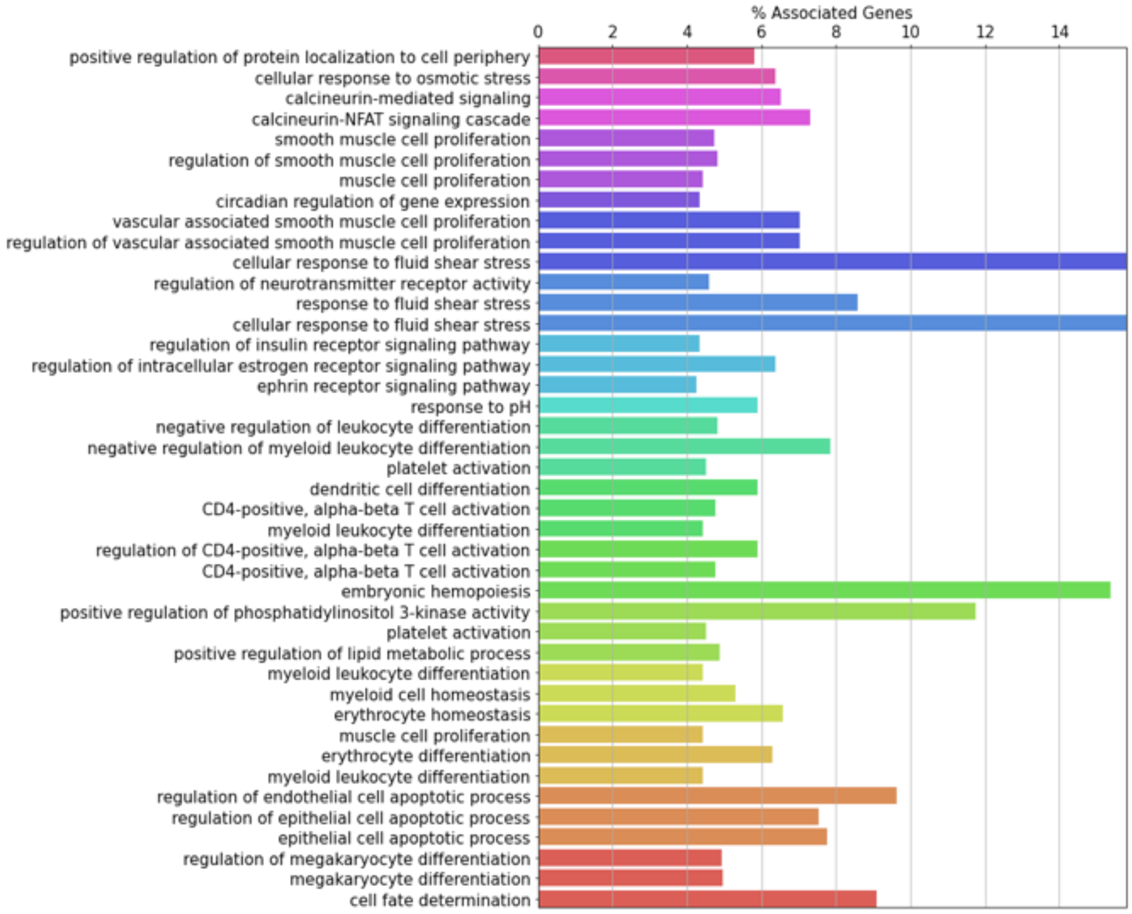

Supplementary Figure S10. Distribution of enriched GO terms in GO analysis of TAF1B predicted as the inhibitory target. The bar chart shows the result of GO analysis of the biological process. GO analysis was performed based on differentially expressed genes of gene expression signatures following TAF1B knock-down. The x axis represents the ratio of associated genes to all genes in the term. The color represents the GO groups. Since the number of enriched GO terms was too large, the top 3 GO terms with high significance in each GO group were displayed. The functionally grouped network of these GO terms is shown in Supplementary Fig. 9 (A).

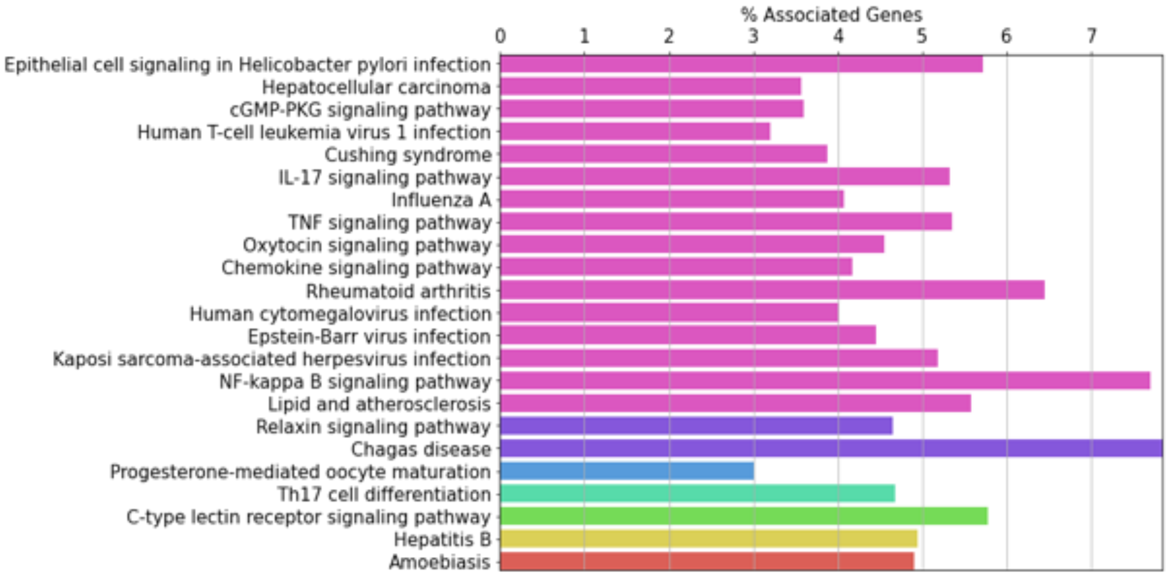

Supplementary Figure S11. Distribution of enriched pathways in the pathway analysis of TAF1B predicted as the inhibitory target. The bar chart shows the result of KEGG pathway analysis of the biological process. This analysis was performed based on differentially expressed genes of gene expression signatures following TAF1B knock-down. The x axis represents the ratio of associated genes to all genes in the pathway. The color represents the KEGG pathway groups. The functionally grouped network of these KEGG pathway terms is shown in Supplementary Fig. 9 (B).

Supplementary Table S1. Cell line list of genetically perturbed gene expression profiles. The gene knockdown profiles consist of data from 17 cell lines and the gene overexpression profiles from 10 cell lines. These cell lines are listed in ascending order of missing data, and the missing rate of each cell line is shown in the bottom panel of Supplementary Figs. S4-S6.

Type of gene perturbation	Cell lines
Gene knock-down	PC3, MCF7, HA1E, A375, A549, HEPG2, VCAP, HT29, HCC515, HEKTE, NPC, SW480, ASC, SHSY5Y, SKL, HEK293T, NCIH716
Gene over-expression	PC3, A375, HEPG2, HT29, HA1E, MCF7, HCC515, A549, VCAP, HEK293T

Supplementary Table S2. Diseases that had at least one therapeutic target protein. In total, 32 and 15 diseases had at least one inhibitory and activatory target protein, respectively. These diseases are listed in alphabetical order.

Type of therapeutic target protein	Diseases
Inhibitory target protein	acute myeloid leukemia, adult T-cell leukemia, Alzheimer's disease, aplastic anemia, asthma, atopic dermatitis, breast cancer, cervical cancer, chronic lymphocytic leukemia, chronic myeloid leukemia, colorectal cancer, congenital muscular dystrophies, Crohn's disease, endometrial cancer, gastric cancer, hepatitis C, hypercholesterolemia, idiopathic pulmonary fibrosis, inflammatory bowel disease, malignant melanoma, multiple myeloma, ovarian cancer, pancreatic cancer, Parkinson disease, renal cell carcinoma, rheumatoid arthritis, small cell lung cancer, systemic lupus erythematosus, testicular cancer, tuberculosis, type I diabetes mellitus, type II diabetes mellitus
Activatory target protein	acute myeloid leukemia, breast cancer, chronic granulomatous disease, chronic myeloid leukemia, colorectal cancer, Crohn's disease, inflammatory bowel disease, multiple myeloma, ovarian cancer, Parkinson disease, renal cell carcinoma, Rett syndrome, rheumatoid arthritis, type I diabetes mellitus, type II diabetes mellitus

Supplementary Table S3. Leaf nodes belonging to the clusters formed by the hierarchical clustering for knocked down genes. 'Cluster node id' corresponds to the cluster node ids in the Supplementary Fig. S2 (A). The nodes of the clusters are listed in the 'Knocked down genes' column. The number of knocked down genes belonging to the clusters are shown in the 'Count' column represents.

Cluster node id	Knocked down genes	Count
1	['AARS', 'ABCC1', 'ACACB', 'ACADSB', 'ADH6', 'ADNP2', 'AGPAT2', 'AGTR1', 'AKAP17A', 'AKR1C2', 'AKT1S1', 'ALDH4A1', 'ANAPC5', 'ANKRD37', 'ANP32E', 'AP2A1', 'AP4S1', 'APAF1', 'APBB2', 'APOE', 'ARHGEF2', 'ARX', 'ASAH1', 'ASAP2', 'ASMTL', 'ASPH', 'ATF1', 'ATF4', 'ATF5', 'ATG4A', 'ATG4B', 'ATIC', 'ATOX1', 'ATP5G1', 'B2M', 'BAD', 'BAMBI', 'BARX1', 'BATF3', 'BCKDK', 'BCL2A1', 'BCL9L', 'BHMT', 'BNIPL', 'BOLA1', 'BRCA1', 'BUD31', 'C12ORF51', 'C5', 'CACNA1C', 'CAMK1', 'CAPSL', 'CASP10', 'CASP2', 'CBLC', 'CBX3', 'CCL2', 'CCNB1', 'CCRN4L', 'CD55', 'CD81', 'CDK10', 'CDK2', 'CDK6', 'CDK9', 'CDKAL1', 'CDKL4', 'CELSR2', 'CEP57', 'CFD', 'CHAF1B', 'CHMP6', 'CIITA', 'CISD1', 'CLK1', 'CNNM2', 'COBL', 'COX6C', 'CPT2', 'CREB3', 'CREM', 'CSF3', 'CSK', 'CTLA4', 'CXCR4', 'DFNB31', 'DGKA', 'DHTKD1', 'DIS3', 'DLL1', 'DNAJC15', 'DNAJC17', 'DR1', 'DUSP1', 'DUSP9', 'EBF1', 'ECI1', 'EED', 'EGF', 'ELF1', 'ELK3', 'EPHX2', 'EPO', 'EPOR', 'ERBB4', 'ERF', 'EXT1', 'EXT2', 'FADD', 'FAT4', 'FBRS', 'FERD3L', 'FLT1', 'FLT3', 'FOXA1', 'FTH1', 'GAK', 'GK', 'GLIS3', 'GLOD4', 'GLTSCR1', 'GLUD2', 'GNAS', 'GPR4', 'GPR82', 'GRPR', 'HCAR1', 'HCFC1', 'HDAC7', 'HMGB2', 'HNF4A', 'HNMT', 'HOXB2', 'HOXB4', 'HOXB7', 'HSBP1', 'HSP90AB1', 'ICAM3', 'ID1', 'IFI30', 'IGF2BP2', 'IGF2BP3', 'IL10', 'IL5', 'IL6', 'INHBE', 'IP6K2', 'IRF1', 'IRF6', 'IRF7', 'IRGC', 'ITGA3', 'JAK2', 'JMJD7-PLA2G4B', 'KCNN4', 'KCTD5', 'KDM5B', 'KLF14', 'KRT26', 'LAMB1', 'LCMT1', 'LDHA', 'LEPRE1', 'LGALS1', 'LGMN', 'LGR5', 'LRP6', 'LRPAP1', 'LRRC16A', 'MAN1A1', 'MAP3K1', 'MAPK3', 'MAPK7', 'MAPK9', 'MAPKAPK2', 'MAT2B', 'MCCC2', 'MCM7', 'MDC1', 'MEIS2', 'MELK', 'MIF', 'MIXL1', 'MLLT10', 'MMP7', 'MSX1', 'MXD4', 'MYL9', 'NAA25', 'NAGPA', 'NCF2', 'NDFIP1', 'NDUFA3', 'NDUFS7', 'NDUFS8', 'NEDD4', 'NFE2L1', 'NFIC', 'NFKB1', 'NKIRAS2', 'NPSR1', 'NR1H3', 'NRBF2', 'NRP1', 'OGDH', 'OIT3', 'P2RY2', 'PAH', 'PAQR7', 'PAQR8', 'PARK7', 'PBX1', 'PCCB', 'PCGF1', 'PCGF2', 'PCGF3', 'PCGF6', 'PCK1', 'PCK2', 'PEBP1', 'PEMT', 'PGF', 'PHF16', 'PIK3C2A', 'PIK3C2B', 'PIK3CA', 'PKN1', 'PLA2G12B', 'PLD2', 'PLXNA1', 'POLQ', 'POLR1A', 'POT1', 'POU2F1', 'PPARA', 'PPM1L', 'PPP1R13B', 'PPP2R2C', 'PRDM1', 'PRKAA2', 'PRKAB1', 'PRKAB2', 'PRKAG3', 'PRKAR1B', 'PRKAR2A', 'PROKR2', 'PROS1', 'PRSS23', 'PTDSS1', 'PTEN', 'PTP4A3', 'PTPN4', 'QDPR', 'RASGRP4', 'RBBP4', 'RBL1', 'RBM6', 'RELA', 'RFXANK', 'RNF5', 'RNF7', 'ROCK1', 'RPF1', 'RPS6KA3', 'RSPH1', 'S1PR3', 'SAFB', 'SCN9A', 'SERPINB2', 'SERPINB5', 'SETD1B', 'SH2B3', 'SIRPG', 'SIRT7', 'SLC16A2', 'SLC1A5', 'SLC29A2', 'SLC38A2', 'SLC3A1', 'SLC7A11', 'SLC7A5', 'SMARCA4', 'SNAI3', 'SNAPC4', 'SP100', 'SQLE', 'SRD5A1', 'SRSF2', 'SRSF8', 'SSX3', 'STAT4', 'STK10', 'STK19', 'SULT2A1', 'SYK', 'TAF12', 'TAF13', 'TCEB3', 'TEAD2', 'TEX10', 'TGM2', 'TLK2', 'TM7SF2', 'TNPO3', 'TRAF2', 'TRAF6', 'TSC1', 'TSKU', 'TWIST2', 'TXLNA', 'UCK2', 'UGT2B28', 'ULK2', 'UQCRB', 'USP9X', 'WNT9B', 'XK', 'YBX1', 'YWHAZ', 'ZBTB24', 'ZBTB26', 'ZFP1', 'ZKSCAN2', 'ZNF114', 'ZNF207', 'ZNF212', 'ZNF213', 'ZNF238', 'ZNF268', 'ZNF273', 'ZNF277', 'ZNF333', 'ZNF395', 'ZNF444', 'ZNF502', 'ZNF643', 'ZNF653', 'ZNF764', 'ZNF77', 'ZNF92']	340
2	['ABCB6', 'ABCC4', 'ABCF1', 'ABCF2', 'ACTR3', 'ACVR1', 'ADI1', 'ADK', 'ADRB2', 'AHRR', 'AIFM1', 'AIM2', 'AKAP1', 'ALDH1B1', 'ALDH3B2', 'ALG9', 'ANKZF1', 'APEX1', 'APOA2', 'APOC3', 'ARHGDIB', 'ARHGEF11', 'ARL4C', 'ARNTL2', 'ASCC3', 'ASH2L', 'ASS1', 'ATP5O', 'ATP5S', 'ATP6V0A1', 'AXIN1', 'BCL2L2', 'BDKRB2', 'BLNK', 'BLZF1', 'BMI1', 'BNIP2', 'BRD2', 'BRD3', 'BRD8', 'C1QBP', 'CACNA1D', 'CALU', 'CAND2', 'CARD11', 'CASK', 'CASP4', 'CASP9', 'CBX1', 'CCBL1', 'CCNG2', 'CD44', 'CD46', 'CD99', 'CDKN2B', 'CDX1', 'CEPT1', 'CERK', 'CHAC1', 'CHRM3', 'CHUK', 'CIAPIN1', 'CLASRP', 'CLN3', 'CLPTM1', 'CLTC', 'CNOT4', 'COASY', 'COQ3', 'COX5A', 'CP', 'CPS1', 'CRCP', 'CREB1', 'CREBBP', 'CRELD2', 'CSDA', 'CSNK1A1L', 'CSNK2A1', 'CSNK2A2', 'CSNK2B', 'CTTN', 'CXCL2', 'CXCR2', 'CYB561', 'CYP1B1', 'CYP24A1', 'DBI', 'DDAH1', 'DDC', 'DFFA', 'DGKH', 'DHX16', 'DHX8', 'DLAT', 'DLG5', 'DMTF1', 'DNAJB1', 'DNAJB6', 'DNMT3A', 'DNMT3L', 'DRAP1', 'DUSP14', 'DVL1', 'E2F6', 'ECH1', 'ECHS1', 'EDNRB', 'EFNB2', 'EGLN2', 'EGR2', 'EHMT2', 'EIF2C3', 'EIF4E', 'EIF6', 'EMD', 'ENO1', 'EP300', 'EPHA2', 'ERCC4', 'ERN1', 'ERO1L', 'ERRFI1', 'ETV6', 'EWSR1', 'EXOC6', 'F5', 'FAM20B', 'FAM3C', 'FBXL19', 'FEN1', 'FER', 'FGB', 'FGFR4', 'FKBP4', 'FKBP8', 'FOXO1', 'FTCD', 'FUS', 'GABARAPL1', 'GABRB3', 'GABRP', 'GALE', 'GALT', 'GAS6', 'GATAD2B', 'GBA', 'GCDH', 'GFPT2', 'GGA2', 'GIT2', 'GJA1', 'GLI1', 'GMEB2', 'GNGT2', 'GPATCH8', 'GPR123', 'GPR150', 'GPR160', 'GPR172A', 'GPR179', 'GPR34', 'GPR35', 'GPR61', 'GPRC5A', 'GPRC5B', 'GPX3', 'GRB10', 'GRIN3A', 'GRK6', 'GSTP1', 'GTF2A1', 'GTF2A2', 'GTF2F2', 'HADHB', 'HAGH', 'HBE1', 'HDAC2', 'HEBP1', 'HERC6', 'HIST1H2AL', 'HLF', 'HMGA2', 'HMGCS2', 'HMOX1', 'HOMEZ', 'HOXA9', 'HOXB13', 'HOXC4', 'HS3ST3A1', 'HSD17B7', 'HSPA2', 'HSPA4', 'HTATIP2', 'HTATSF1', 'IARS', 'ICMT', 'IDH1', 'IDH2', 'IER3', 'IFNAR2', 'IFNB1', 'IFNGR1', 'IFNGR2', 'IGFBP4', 'IGHMBP2', 'IL13RA1', 'IL15RA', 'IL18', 'IL7R', 'IL8', 'IMPDH1', 'IMPDH2', 'INPP4B', 'INS', 'INTS12', 'IRAK4', 'IRF3', 'ITGA1', 'ITGA4', 'ITGAE', 'ITGB1', 'ITGB2', 'ITGB5', 'ITPK1', 'KCNMA1', 'KHDRBS1', 'KIAA0922', 'KIN', 'KLHL21', 'KRT8', 'KYNU', 'LAGE3', 'LAMP2', 'LBR', 'LDLR', 'LGALS8', 'LHX4', 'LIF', 'LMAN1', 'LPAR1', 'LPGAT1', 'LPL', 'LRP5', 'LRRC40', 'LSR', 'LYAR', 'LYPLA1', 'MAF', 'MALT1', 'MAN2B1', 'MAOB', 'MAP2K4', 'MARK4', 'MAS1L', 'MAST2', 'MBNL2', 'MBOAT7', 'MDM4', 'MED28', 'MEF2C', 'MET', 'MICALL1', 'MKNK1', 'MLH1', 'MNAT1', 'MOK', 'MPHOSPH9', 'MRGPRX2', 'MTRR', 'MVP', 'MYCL2', 'MYLIP', 'MYLK2', 'NAA50', 'NARS', 'NCAPD2', 'NCL', 'NDUFA13', 'NDUFA8', 'NDUFA9', 'NDUFB6', 'NDUFB8', 'NDUFS2', 'NFATC3', 'NFKBIE', 'NID1', 'NIPSNAP1', 'NKX2-1', 'NLK', 'NNT', 'NONO', 'NPDC1', 'NPEPPS', 'NR4A2', 'NSD1', 'NSFL1C', 'NTSR1', 'NUP62', 'NUP85', 'OPN3', 'OPN5', 'OSGEP', 'OSR1', 'OSR2', 'OTX1', 'OTX2', 'OXSR1', 'P2RY8', 'PAICS', 'PANK2', 'PAPSS1', 'PARP10', 'PDGFRA', 'PECR', 'PER1', 'PEX19', 'PGM1', 'PHTF1', 'PIM3', 'PIN1', 'PITPNA', 'PKIA', 'PLA2G2C', 'PLAU', 'PLCB1', 'PLCG1', 'PLOD2', 'PLXNA2', 'PNLDC1', 'PNRC1', 'POLD4', 'POLR3B', 'PON2', 'POR', 'POU5F1', 'POU5F2', 'PPP2R5A', 'PPP2R5E', 'PPP3CC', 'PPP3R1', 'PRDX2', 'PREP', 'PRKCZ', 'PRLR', 'PRPF4', 'PRUNE', 'PSMB8', 'PSMD10', 'PSME2', 'PTK2B', 'PTPN12', 'PTPRF', 'PTPRG', 'PTS', 'PURA', 'PWP1', 'PXMP2', 'PYGL', 'QARS', 'RAB11FIP2', 'RAB4A', 'RAB5B', 'RAD23B',	505

	'RARB', 'RASSF5', 'RB1', 'RDH11', 'RFX2', 'RGS4', 'RHEB', 'RIMS3', 'RIPK2', 'RIPK4', 'RNASE4', 'RND3', 'RNF145', 'RPP38', 'RPS6KA1', 'RPS6KB1', 'RRAGB', 'RSU1', 'RXRB', 'S100A1', 'S100P', 'S1PR2', 'SACM1L', 'SAR1B', 'SBNO1', 'SCUBE1', 'SEPX1', 'SERPINB6', 'SERPINE1', 'SERTAD3', 'SETD7', 'SF1', 'SGPL1', 'SHANK3', 'SIAH2', 'SIK1', 'SIK3', 'SIRT1', 'SIRT2', 'SIX2', 'SLC12A7', 'SLC16A6', 'SLC22A18', 'SLC25A22', 'SLC25A46', 'SLC2A1', 'SLC30A8', 'SLC36A4', 'SLC7A1', 'SMAD6', 'SMARCB1', 'SMARCC2', 'SMO', 'SOCS2', 'SOCS4', 'SOCS5', 'SORD', 'SORT1', 'SOX11', 'SOX4', 'SP110', 'SPA17', 'SPRY1', 'SRF', 'SRPK1', 'ST3GAL5', 'ST6GALNAC2', 'STAT2', 'STAT5A', 'STK17A', 'STK24', 'STUB1', 'STXBP1', 'STXBP2', 'SULT1A1', 'SULT1A2', 'TAAR6', 'TAAR9', 'TAF15', 'TAF5L', 'TAL2', 'TAP1', 'TARBP2', 'TASP1', 'TCEA1', 'TCF4', 'TCTN1', 'TFF2', 'THBS1', 'TIMM22', 'TMEM50A', 'TNFRSF13B', 'TNFRSF19', 'TNFRSF6B', 'TNFSF13', 'TP53BP1', 'TP53TG1', 'TPMT', 'TPRKB', 'TRAF3', 'TRAF4', 'TRERF1', 'TRIB1', 'TRIM32', 'TSC2', 'TSLP', 'UAP1', 'UBE2E3', 'UBE2J1', 'UBL5', 'UBP1', 'UBR7', 'UFM1', 'UGT1A6', 'UGT1A9', 'VAV3', 'VCAN', 'VN1R2', 'WASF3', 'WNT9A', 'ZAP70', 'ZNF175', 'ZNF195', 'ZNF200', 'ZNF24', 'ZNF454', 'ZNF496', 'ZNF521', 'ZNF546', 'ZNF551', 'ZNF559', 'ZNF583', 'ZNF586', 'ZNF619', 'ZNF677', 'ZNF707', 'ZNF787', 'ZNF805', 'ZSCAN29', 'ZSCAN4', 'ZW10']	
3	['61E3.4', 'ACVR1C', 'ADCK1', 'AK5', 'AK7', 'BRSK2', 'CAMKK1', 'CD2', 'CDC42BPA', 'CDK14', 'CDKL3', 'CERKL', 'CKM', 'CSNK1G1', 'DCLK1', 'DGKE', 'DLG1', 'DLG2', 'DSTYK', 'DYRK1B', 'DYRK4', 'EPHB6', 'FASTKD1', 'FLJ40852', 'GALK2', 'GK5', 'GUCY2C', 'GUCY2F', 'HK2', 'HKDC1', 'IPPK', 'IRAK3', 'LOC391533', 'LOC441708', 'LOC442558', 'LRGUK', 'MAP3K3', 'MAP4K1', 'MAPK4', 'MAST1', 'MORN2', 'MPP1', 'MPP2', 'MPP5', 'MPP6', 'MPP7', 'MST4', 'MVK', 'N4BP2', 'NEK1', 'NEK3', 'NPR2', 'NRK', 'PAK6', 'PANK1', 'PASK', 'PDGFRL', 'PDIK1L', 'PFKFB2', 'PHKA2', 'PIK3C2G', 'PIK3R5', 'PIP5K1A', 'PKLR', 'PRKY', 'RNASEL', 'SEPHS2', 'SGK2', 'SGK223', 'SGK494', 'SH3BP5L', 'SIK2', 'TAF1L', 'TGFBR3', 'TNK2', 'TNNI3K', 'TRIB2', 'TRPM6', 'WNK3']	79
4	['C19ORF35', 'CAMK1D', 'CKMT1B', 'DDR2', 'DGKG', 'DYRK2', 'FRK', 'HUNK', 'INSRR', 'ITPKC', 'LMTK2', 'LOC389906', 'LOC441047', 'LOC442313', 'MAK', 'MAP3K12', 'MAP3K6', 'MPP3', 'NEK5', 'NME2', 'PI4KB', 'PION', 'PIP5K1C', 'PRPS1L1', 'ROR1', 'TXNDC3', 'ULK4']	27
5	['ABCA1', 'ABCA5', 'ABCB4', 'ABCB5', 'ABCB7', 'ABCC10', 'ABCC2', 'ABCG2', 'ABHD4', 'ACD', 'ACSL1', 'ADAM10', 'ADAT1', 'ADCY5', 'ADORA2A', 'ADORA2B', 'ADSL', 'ADSS', 'AFF1', 'AIRE', 'ALAS1', 'ALDH2', 'ALDH9A1', 'ALG5', 'AMBP', 'AP1B1', 'AP1S2', 'APOBEC3H', 'ARF1', 'ARF6', 'ARHGAP1', 'ARHGEF7', 'ARID4B', 'ARID5B', 'ARPC1B', 'ATF2', 'ATF3', 'ATG12', 'ATG4D', 'AZGP1', 'BACE2', 'BAG1', 'BAZ2B', 'BCAT2', 'BCKDHA', 'BCL6', 'BCLAF1', 'BCR', 'BDNF', 'BHLHE40', 'BLCAP', 'BMPR2', 'BNIP3', 'BNIP3L', 'BRCA2', 'BRD1', 'BRDT', 'BRP44', 'BTG3', 'BUD13', 'C15ORF42', 'C16ORF5', 'C1ORF106', 'CAB39', 'CABIN1', 'CALM2', 'CALM3', 'CAMK1G', 'CAMK4', 'CANT1', 'CANX', 'CARD10', 'CASP8', 'CBLB', 'CCDC90A', 'CCDC92', 'CCND2', 'CCNE1', 'CD14', 'CDC25B', 'CDCA4', 'CDK3', 'CDKN1C', 'CDKN2A', 'CDKN2C', 'CDX2', 'CEACAM1', 'CFLAR', 'CHRM1', 'CIR1', 'CKS2', 'CLPX', 'CLSTN1', 'CMAS', 'CNDP2', 'COPS5', 'COX7B', 'CPD', 'CPNE3', 'CPSF4', 'CPT1A', 'CRABP1', 'CRY1', 'CSNK1D', 'CTBP1', 'CTCFL', 'CTNNA1', 'CTSD', 'CUL2', 'DAG1', 'DAP', 'DCTD', 'DCXR', 'DDAH2', 'DENND1B', 'DFFB', 'DGKZ', 'DLD', 'DNM1', 'DNM1L', 'DOK4', 'DPH2', 'DPYD', 'DRP2', 'DTYMK', 'DUSP11', 'DUSP2', 'DUSP3', 'E2F1', 'EBNA1BP2', 'EEF1D', 'EEF2K', 'EGR1', 'EGR3', 'EHF', 'EIF2AK1', 'EIF2AK4', 'EIF4G1', 'EIF5', 'ELAVL1', 'ELOVL6', 'EML3', 'ENOPH1', 'EPCAM', 'EPHB2', 'EPRS', 'ERCC2', 'ERCC3', 'ESD', 'ESRRA', 'ETNK2', 'EVL', 'EXOC2', 'F2RL1', 'FAF1', 'FAIM', 'FAM120A', 'FAM63A', 'FANCG', 'FASTKD5', 'FAU', 'FBXL20', 'FBXW11', 'FEZ1', 'FGFRL1', 'FGGY', 'FHL2', 'FIBP', 'FIS1', 'FKBP1A', 'FN1', 'FOS', 'FOXD4L3', 'FOXP3', 'FUT1', 'FUT2', 'G3BP2', 'GABPB1', 'GABRA5', 'GALNT1', 'GAPDH', 'GARS', 'GFPT1', 'GGCX', 'GHR', 'GIPC1', 'GLUL', 'GNA15', 'GNB1L', 'GNG4', 'GNPDA1', 'GOT1', 'GPR114', 'GPR133', 'GSK3A', 'GSS', 'GSTT1', 'GTF2B', 'GYS1', 'H2AFX', 'HADHA', 'HAX1', 'HCN3', 'HDAC4', 'HDAC5', 'HDAC9', 'HEXIM1', 'HEY1', 'HIBADH', 'HIF1A', 'HINT1', 'HIPK4', 'HIST1H1C', 'HIST1H3B', 'HK1', 'HLA-DMA', 'HMBOX1', 'HMG20B', 'HMGCL', 'HMGN3', 'HNF1A', 'HOXA10', 'HOXA2', 'HOXC9', 'HPN', 'HRH1', 'HSD17B10', 'HSPA8', 'HTR2C', 'HUS1', 'ID4', 'IFI16', 'IFIH1', 'IFIT5', 'IFRD2', 'IGFN1', 'IGSF8', 'IKBKB', 'IKBKG', 'IL11', 'IL12B', 'IL13RA2', 'IL1RAP', 'IL6R', 'ING2', 'INPP1', 'INTS3', 'IPMK', 'IPO13', 'IRAK1', 'IRF5', 'IRGM', 'ITGB1BP3', 'ITPR3', 'KBTBD2', 'KDELR2', 'KIAA0317', 'KIAA0494', 'KIAA1267', 'KIF21B', 'KLF5', 'KLF6', 'KLRC1', 'L3MBTL4', 'LAMA3', 'LAMTOR3', 'LANCL1', 'LCN2', 'LGR4', 'LHPP', 'LHX3', 'LHX8', 'LIPA', 'LMNB2', 'LOC390877', 'LOC392265', 'LOC400301', 'LOC441971', 'LPAR6', 'LRRC18', 'LYRM1', 'M6PR', 'MAGI3', 'MAML1', 'MAMLD1', 'MAP2K3', 'MAP3K11', 'MAP3K2', 'MAPK1', 'MAPK13', 'MAPK15', 'MAPK6', 'MAX', 'MCM4', 'MECP2', 'MED12L', 'MED7', 'METTL1', 'MFHAS1', 'MGMT', 'MIB1', 'MINK1', 'MMEL1', 'MORN1', 'MPDZ', 'MPL', 'MPZL1', 'MRGPRD', 'MST1', 'MSX2', 'MTAP', 'MTERFD1', 'MTHFD1', 'MUC1', 'MYCBP', 'MYCBP2', 'MYCL1', 'MYLK', 'MYO3B', 'NAMPT', 'NAT1', 'NCOA2', 'NDUFB2', 'NDUFS1', 'NEK2', 'NEK8', 'NET1', 'NEUROD1', 'NF2', 'NFIL3', 'NIPBL', 'NME3', 'NME5', 'NMT1', 'NR1D2', 'NR1H2', 'NR2C1', 'NR2F1', 'NRGN', 'NRXN3', 'NSA2', 'NT5DC2', 'NTRK3', 'NUDT14', 'OAS1', 'ODC1', 'OR10Z1', 'OVCA2', 'OXGR1', 'P2RY6', 'PAFAH1B1', 'PAK3', 'PAK7', 'PANK3', 'PARP1', 'PARP2', 'PASD1', 'PBK', 'PCDHA10', 'PCLO', 'PDIA5', 'PDLIM1', 'PDXK', 'PEAK1', 'PEX12', 'PGRMC1', 'PHGDH', 'PHKG2', 'PIKFYVE', 'PIM2', 'PLA2G15', 'PLEKHJ1', 'PLK3', 'PLS1', 'PLXNA3', 'PNCK', 'POLA1', 'POLA2', 'POLG2', 'POLR2K', 'POLR3D', 'PPARD', 'PPIG', 'PPM1B', 'PPP2CA', 'PPP2R3C', 'PRDX5', 'PRKCE', 'PRKCH', 'PRKCI', 'PRKRA', 'PRKRIR', 'PRKX', 'PRMT3', 'PSIP1', 'PSMD8', 'PSMG1', 'PTPN11', 'PTPN2', 'PTPN21', 'PTPN22', 'PTPRK', 'PYCR1', 'RAB21', 'RABGGTA', 'RAD1', 'RAD51C', 'RAP1GAP', 'RARA', 'RBBP6', 'RBBP8', 'RBL2', 'RBP4', 'RCAN1', 'REEP5', 'RELB', 'RFC1', 'RFC5', 'RFX6', 'RG9MTD1', 'RNF133', 'RPIA', 'RPL39L', 'RPS6KB2', 'RXFP1', 'RYBP', 'SCAP', 'SCP2', 'SCYL1', 'SDHC', 'SEC14L1', 'SERPIND1', 'SFMBT1', 'SH3BP5', 'SHH', 'SHMT2', 'SLC11A2', 'SLC12A2', 'SLC16A4', 'SLC1A1', 'SLC1A4', 'SLC25A13', 'SLC25A15', 'SLC25A20', 'SLC2A4RG', 'SLC2A6', 'SLC37A4', 'SLC3A2', 'SLC5A6', 'SLC6A8', 'SMAD3', 'SMARCE1', 'SMC3', 'SMU1', 'SNX11', 'SNX2', 'SNX7', 'SOCS3', 'SOS1', 'SOX5', 'SP140', 'SP140L', 'SPDEF', 'SPECC1L', 'SPEN', 'SPHK2', 'SQSTM1', 'ST13', 'STAP2', 'STK11', 'STK38', 'STK38L', 'SUB1', 'SUCNR1', 'SYNGR3', 'TAL1', 'TAOK2', 'TARBP1', 'TBX3', 'TBXA2R', 'TCEAL4', 'TCF7L2', 'TCTA', 'TEAD4', 'TERF2IP', 'TES', 'TEX14', 'TFAP2A', 'TGFBR1', 'TIMP1', 'TJP1', 'TK2', 'TMCO1', 'TMED7', 'TMEM127', 'TNFRSF10A', 'TNFRSF1A', 'TNFSF13B', 'TNKS2', 'TOMM40', 'TOP2A', 'TOPBP1', 'TP53', 'TP53BP2', 'TPD52L3', 'TPM1', 'TPR', 'TRADD', 'TRAF7', 'TRIB3', 'TRIM24', 'TRIM27', 'TSEN2', 'TSN', 'TTBK1', 'TTF1', 'TXNDC9', 'TXNIP', 'TXNRD2', 'TYK2', 'UBAP1', 'UBAP2L', 'UBE2D3', 'UBE2K', 'UCKL1', 'UQCRFS1', 'USP12', 'USP15', 'VAPA', 'VAPB', 'VARS', 'VAT1', 'VDR', 'VEGFA', 'VEGFB', 'VIPR1', 'VKORC1', 'VN1R4', 'WDTC1', 'WNK4', 'WRB',	568

	'WWTR1', 'XPC', 'XPO7', 'XPR1', 'XRCC6BP1', 'YES1', 'ZBTB5', 'ZC3H4', 'ZDHHC6', 'ZFP28', 'ZMPSTE24', 'ZNF134', 'ZNF140', 'ZNF266', 'ZNF274', 'ZNF32', 'ZNF384', 'ZNF473', 'ZNF486', 'ZNF554', 'ZNF577', 'ZNF582', 'ZNF611', 'ZNF621', 'ZNF625', 'ZNF672', 'ZRSR2']	
6	['AATK', 'ADCK2', 'ALPK1', 'CKMT2', 'DGKQ', 'DNAJC6', 'EPHA4', 'FASTKD3', 'GOLGA5', 'GRK1', 'HK3', 'LATS1', 'LATS2', 'LOC441777', 'MAP3K10', 'NEK10', 'NTPCR', 'NTRK2', 'PAPSS2', 'PKDCC', 'PLXNB1', 'PRKD3', 'RIOK1', 'RP2', 'SGK196', 'SHPK', 'SPHK1', 'STK31', 'STRADA', 'STYK1', 'TAOK1', 'TBCK', 'TSSK2', 'TSSK3', 'UCK1', 'UHMK1', 'YSK4']	37
7	['A2M', 'ABCG5', 'AGPAT1', 'AK1', 'ALDH3A1', 'ALDH3A2', 'ANXA3', 'APOM', 'APOO', 'ARHGEF6', 'ASF1B', 'ATG16L1', 'ATP1A1', 'ATP5C1', 'ATP5H', 'AURKC', 'BACH1', 'BCL2L11', 'BCL6B', 'BMP2', 'BMX', 'BNIP1', 'BRF2', 'BUB1', 'C17ORF75', 'C2', 'CALML6', 'CAMK2B', 'CBX7', 'CCRL2', 'CD1C', 'CDC20', 'CDIPT', 'CDK18', 'CEBPB', 'CELSR3', 'CERS4', 'CFB', 'CIB4', 'CISH', 'CLOCK', 'CREB3L1', 'CRYAA', 'CTSK', 'CXADR', 'CXCR7', 'CYLD', 'DAPK2', 'DCUN1D4', 'DGCR14', 'DHH', 'DKC1', 'DNA2', 'DTX1', 'DTX2', 'DTX3L', 'DUSP7', 'DVL3', 'E2F8', 'EIF5A', 'EPHA3', 'EPHB3', 'EPN2', 'ESYT1', 'EXOSC10', 'F10', 'FABP1', 'FADS3', 'FGA', 'FN3K', 'FOXF2', 'FOXJ2', 'FOXN3', 'FRAT2', 'FRS2', 'FXN', 'FZD2', 'FZD6', 'GABBR1', 'GAS7', 'GCLM', 'GLDC', 'GMPR2', 'GNAQ', 'GPR137', 'GPR151', 'GPR19', 'GPR83', 'GPRC6A', 'GPSM2', 'GPX1', 'GRK7', 'GSG2', 'GTF3C2', 'HARS', 'HBP1', 'HDAC8', 'HES5', 'HIC2', 'HLA-DPB1', 'HMGA1', 'HOPX', 'HOXA6', 'HSD17B2', 'HSD17B8', 'HSF1', 'HSF2', 'HSPA1B', 'IGFBP3', 'IGFBP6', 'IGFBP7', 'IL1R2', 'ING1', 'ITGB4', 'ITPKA', 'ITSN1', 'JUNB', 'KITLG', 'KLF4', 'KLF7', 'KLK6', 'LEF1', 'LEPREL1', 'LGALS3', 'LHX9', 'LIN28B', 'LIPI', 'LIPT1', 'LMNA', 'LPHN1', 'LSM2', 'MAGED1', 'MAP3K5', 'MAST3', 'MAST4', 'MATK', 'MCHR2', 'MCL1', 'MCOLN1', 'MDH1', 'MEF2BNB-MEF2B', 'MFAP3', 'MLL3', 'MLLT3', 'MMP1', 'MRGPRX1', 'MRGPRX3', 'MRGPRX4', 'MTR', 'MTSS1', 'MYNN', 'NAT10', 'NDUFAB1', 'NDUFB1', 'NDUFS4', 'NEK9', 'NFATC2', 'NFKBIL1', 'NFRKB', 'NGRN', 'NKIRAS1', 'NOV', 'NPFFR2', 'O3FAR1', 'OAT', 'OAZ1', 'OR52B2', 'ORM1', 'OTUD3', 'P2RY12', 'PA2G4', 'PAFAH1B2', 'PAM', 'PBXIP1', 'PER2', 'PHF19', 'PHLPP2', 'PI4K2B', 'PIAS3', 'PIGA', 'PIK3C3', 'PLA2G2A', 'POLE3', 'POLG', 'POLR1E', 'POLRMT', 'POU2AF1', 'PPIB', 'PRKG1', 'PRLHR', 'PROKR1', 'PRPS1', 'PRPS2', 'PRSS3', 'PSKH2', 'PSMA8', 'PSMB10', 'PSMB9', 'PTF1A', 'PTGER4', 'PVRL2', 'QRFPR', 'RARRES1', 'RBBP7', 'RBMS1', 'RCCD1', 'RCHY1', 'RHOD', 'RNLS', 'RRAGD', 'RTN2', 'RUNX1', 'S100B', 'SATB2', 'SCYL2', 'SERPINA6', 'SERPINA7', 'SERPINH1', 'SIN3A', 'SIX4', 'SLC1A6', 'SLC22A4', 'SLC27A3', 'SLC38A3', 'SNX16', 'SPARC', 'SPRED2', 'SPRY4', 'SREBF1', 'SRGAP2', 'SRPX', 'SSX2', 'SUOX', 'TAAR1', 'TADA3', 'TBP', 'TBX5', 'TCF7L1', 'TDRD9', 'TESK2', 'TFAP4', 'TGIF2LY', 'TIMM50', 'TINF2', 'TMED9', 'TNF', 'TNFSF10', 'TRA2B', 'TRPM7', 'TTBK2', 'TXK', 'TXNRD3', 'UBE2N', 'UBTF', 'UCHL1', 'UNC5D', 'UNG', 'VEGFC', 'VN1R5', 'WNK2', 'WNT11', 'XRCC4', 'ZBED1', 'ZC3HC1', 'ZNF133', 'ZNF169', 'ZNF180', 'ZNF267', 'ZNF354B', 'ZNF436', 'ZNF462', 'ZNF490', 'ZNF519', 'ZNF548', 'ZNF558', 'ZNF563', 'ZNF57', 'ZNF576', 'ZNF585A', 'ZNF595', 'ZNF74', 'ZNF830', 'ZSWIM2']	283
8	['ABAT', 'ABCB1', 'ACLY', 'ADA', 'ADAM15', 'ADC', 'AFP', 'AHR', 'AKAP11', 'AKAP13', 'AKAP8L', 'AKAP9', 'AKR1C1', 'AKR7A2', 'AKTIP', 'ALAD', 'ALDH18A1', 'ALDH1A3', 'ALDH5A1', 'ALDH6A1', 'ALMS1', 'ANXA2', 'ANXA5', 'ANXA7', 'ARG1', 'ARPC2', 'ATG16L2', 'ATG3', 'ATG7', 'ATP5L', 'ATP6AP1', 'ATP6V1D', 'ATR', 'AURKAIP1', 'BAG3', 'BAG6', 'BCL3', 'BECN1', 'BIRC6', 'BMPR1A', 'BPHL', 'BTG2', 'BUB3', 'C19ORF10', 'C1ORF9', 'C2CD2', 'C2ORF3', 'CA2', 'CALR', 'CASP3', 'CBX6', 'CCL20', 'CCNB2', 'CCNG1', 'CD40', 'CD58', 'CDC7', 'CDK4', 'CDKL5', 'CDKN1B', 'CEACAM6', 'CEBPA', 'CEP72', 'CES1', 'CHCHD7', 'CHERP', 'CLEC16A', 'CLK3', 'CMPK1', 'CNOT3', 'COG7', 'COPS2', 'CRABP2', 'CRADD', 'CS', 'CSMD1', 'CSNK1A1', 'CTNNB1', 'CTSB', 'CTSL1', 'CXCL12', 'CYB5A', 'CYCS', 'DAXX', 'DDB2', 'DDX10', 'DDX5', 'DENND2D', 'DEPTOR', 'DERA', 'DHCR7', 'DHFR', 'DICER1', 'DIXDC1', 'DMD', 'DOT1L', 'DPF1', 'EBP', 'EIF2A', 'EIF2AK2', 'EIF2C2', 'ENOSF1', 'EPAS1', 'EPHB4', 'ERAP2', 'ERCC1', 'ETS2', 'FAM69A', 'FASN', 'FBXO7', 'FDFT1', 'FDPS', 'FERMT1', 'FGF10', 'FGFR1OP', 'FLI1', 'FOXQ1', 'FZD5', 'G2E3', 'GATA3', 'GATA6', 'GCH1', 'GDPD5', 'GFOD1', 'GGPS1', 'GLUD1', 'GMNN', 'GNAL', 'GPC1', 'GPER', 'GRB2', 'GRK5', 'GRWD1', 'GSDMB', 'H2AFV', 'HDAC11', 'HLA-B', 'HLA-DPA1', 'HOOK2', 'HORMAD2', 'HOXC10', 'HS2ST1', 'HSD17B11', 'HSPA14', 'HSPD1', 'ID2', 'IL18RAP', 'IL20', 'IL4R', 'ILF3', 'IMPA1', 'IPO4', 'ITPR2', 'KCNK15', 'KDM3A', 'KDM5A', 'KIAA0753', 'KIAA0907', 'KIF2C', 'KISS1R', 'KLF11', 'LACC1', 'LARS2', 'LIN28A', 'LTB', 'LYZ', 'LZIC', 'MAD2L1BP', 'MAP3K14', 'MAP3K4', 'MAP3K8', 'MAPK1IP1L', 'MAT1A', 'MBNL1', 'ME2', 'MED15', 'MEOX2', 'MGAT1', 'MLL4', 'MMP14', 'MRPL18', 'MRPS16', 'MXD3', 'MYBL2', 'NAE1', 'NBR1', 'NCOA4', 'NCOR2', 'NDUFB3', 'NDUFB7', 'NDUFV1', 'NECAB3', 'NEUROD4', 'NF1', 'NFKBIA', 'NFYB', 'NIT1', 'NKX2-5', 'NME1', 'NR2F2', 'NR5A2', 'NT5E', 'NTAN1', 'NUMB', 'NUSAP1', 'ORMDL3', 'OSMR', 'P4HA2', 'PACSIN3', 'PAN2', 'PAPD7', 'PCGF5', 'PDHX', 'PDLIM4', 'PEPD', 'PFAS', 'PFKL', 'PHB', 'PHB2', 'PHKB', 'PI4KA', 'PIAS2', 'PLAUR', 'PLD4', 'PMF1', 'PML', 'PNKP', 'POLB', 'POLE2', 'PPIE', 'PPM1D', 'PPP1CA', 'PPP1CC', 'PPP1R14B', 'PPP2R3A', 'PPP4R1', 'PQBP1', 'PRAF2', 'PREB', 'PRKACB', 'PRKDC', 'PRPSAP2', 'PSMF1', 'PSRC1', 'PTGR1', 'PTPLAD1', 'PUF60', 'PVALB', 'PXK', 'RAB27A', 'RAB3GAP1', 'RAC1', 'RALBP1', 'RAMP1', 'RAP1GDS1', 'RASGRP1', 'RBM15B', 'RDBP', 'RIOK2', 'ROCK2', 'RPN2', 'RRP12', 'RRP8', 'RYK', 'S100A11', 'SALL4', 'SCCPDH', 'SCMH1', 'SEC61A2', 'SENP2', 'SERINC3', 'SESN1', 'SET', 'SGCB', 'SLC29A1', 'SLC30A10', 'SLC30A2', 'SLC35A1', 'SLC39A8', 'SLC6A14', 'SLK', 'SMURF1', 'SMURF2', 'SPR', 'SRC', 'SRM', 'SRPRB', 'SRSF3', 'STAMBP', 'STAT5B', 'STK16', 'STK25', 'STX16', 'SUCLG2', 'SULT1A3', 'SUV39H1', 'SWAP70', 'SYN2', 'TADA2A', 'TAF1B', 'TAGAP', 'TAOK3', 'TARDBP', 'TBX2', 'TCEB3C', 'TFG', 'TGFA', 'TLE2', 'TMSB4XP8', 'TNFAIP1', 'TNFAIP3', 'TNFRSF13C', 'TNFRSF17', 'TNFRSF21', 'TOLLIP', 'TOMM34', 'TRAF1', 'TRAK2', 'TRAM2', 'TRAPPC3', 'TRIP10', 'TRPM5', 'TSG101', 'TSPAN8', 'TST', 'TTK', 'TUBB3', 'UBE2D1', 'UBQLN2', 'UGCG', 'UNC13B', 'UNC5C', 'USF1', 'USP20', 'USP22', 'UVRAG', 'WARS', 'WFDC2', 'XPNPEP1', 'ZFP161', 'ZMIZ1', 'ZMYM2', 'ZNF143', 'ZNF217', 'ZNF365', 'ZNF689']	346
9	['ABCD3', 'ABCF3', 'ABCG8', 'ABL1', 'ACAA1', 'ACACA', 'ACAD8', 'ACADM', 'ACAT1', 'ACOT9', 'ACSL3', 'ACTR2', 'ADAR', 'ADCY9', 'ADH5', 'ADRBK1', 'AFF4', 'AGT', 'AHCY', 'AIP', 'AK2', 'AK3', 'AK4', 'AKT1', 'AKT2', 'AKT3', 'ALB', 'ALX1', 'AMD1', 'AMPD2', 'AMT', 'ANGPT1', 'ANKRD10', 'ANKRD55', 'ANXA1', 'APEH', 'APPBP2', 'ARAF', 'ARAP1', 'ARFRP1', 'ARGLU1', 'ARHGEF12', 'ARID3A', 'ARNT2', 'ARPC1A', 'ARPC4', 'ASL', 'ATG5', 'ATM', 'ATMIN', 'ATP1A3', 'ATP1B1', 'ATP5A1', 'ATP6V0B', 'ATP7A', 'ATXN3', 'AXL', 'B3GAT3', 'B3GNT1', 'BACE1', 'BACH2', 'BAK1', 'BAZ1B', 'BCAR1', 'BCAT1', 'BCL10', 'BCL2', 'BCL2L1', 'BCL7B', 'BDH1', 'BIK', 'BIRC2', 'BLOC1S1', 'BLVRB', 'BMP4', 'BRAF', 'BRPF1', 'BTBD3', 'BTK', 'BTRC', 'BUB1B', 'C3', 'C5ORF62', 'CA12', 'CAD', 'CALCOCO2', 'CAMK2D',	767

	'CAMLG', 'CARS', 'CASC3', 'CASD1', 'CASP14', 'CBFB', 'CBR1', 'CCDC85B', 'CCDC86', 'CCNA2', 'CCND1', 'CCND3', 'CCNF', 'CCNL1', 'CCP110', 'CD19', 'CD320', 'CD79B', 'CD97', 'CDA', 'CDC123', 'CDC25C', 'CDC45', 'CDCA7L', 'CDH1', 'CDH3', 'CDK1', 'CDK16', 'CDK5', 'CDK7', 'CDKN1A', 'CDT1', 'CEBPD', 'CEBPG', 'CERS2', 'CES2', 'CETN2', 'CETN3', 'CHEK1', 'CHEK2', 'CHKA', 'CHKB', 'CHMP2A', 'CHMP4A', 'CIRBP', 'CIT', 'CKB', 'CKS1B', 'CLK2', 'CNPY3', 'COG2', 'COL13A1', 'COL4A2', 'COL4A5', 'COX4I1', 'COX5B', 'COX6B2', 'CPE', 'CRAT', 'CREB3L2', 'CREG1', 'CRK', 'CRTAP', 'CRYZ', 'CTGF', 'CTSL2', 'CYB5R3', 'CYP20A1', 'CYP27B1', 'CYP51A1', 'DAPK1', 'DAPK3', 'DCK', 'DCTN6', 'DDB1', 'DDIT3', 'DDOST', 'DDR1', 'DDX39B', 'DDX42', 'DERL1', 'DHCR24', 'DHDDS', 'DHRS3', 'DIABLO', 'DIDO1', 'DNAJA3', 'DNAJB12', 'DNAJC12', 'DNPEP', 'DSE', 'DSG2', 'DTNA', 'DUSP22', 'DUSP6', 'DVL2', 'DYRK1A', 'E2F3', 'EAPP', 'EDN1', 'EGFR', 'EHHADH', 'EIF2AK3', 'EIF2B2', 'EIF4B', 'EIF4EBP1', 'ELF3', 'ELF4', 'ELL3', 'EMR1', 'ENDOG', 'EPB41L4B', 'EPDR1', 'EPHA6', 'EPHX1', 'EPS8', 'ERBB3', 'ERCC5', 'ERG', 'ERLIN1', 'ETS1', 'ETV4', 'EXO1', 'EXOC3', 'EYA1', 'EZH1', 'F11', 'F12', 'F2', 'F3', 'F7', 'FAAH', 'FABP4', 'FABP5', 'FAH', 'FAM104A', 'FAM57A', 'FANCL', 'FAT1', 'FBXO21', 'FBXO5', 'FCGR2A', 'FCHO1', 'FDXR', 'FES', 'FEZ2', 'FFAR1', 'FGF2', 'FGFR3', 'FKBP14', 'FOLR1', 'FOXA3', 'FOXJ3', 'FOXO3', 'FOXP4', 'FOXR1', 'FPGS', 'FTL', 'FXYD2', 'FYN', 'FZD8', 'G3BP1', 'GABPB2', 'GABRG1', 'GADD45A', 'GADD45B', 'GALNS', 'GAMT', 'GATAD2A', 'GCAT', 'GCK', 'GGCT', 'GLRX', 'GLS', 'GM2A', 'GNA12', 'GNAI2', 'GNB5', 'GNE', 'GOLIM4', 'GPR112', 'GPR115', 'GPR125', 'GPR128', 'GPR132', 'GPR141', 'GPR142', 'GPR146', 'GPR152', 'GPR158', 'GPR62', 'GPR64', 'GPR65', 'GRB7', 'GRHL3', 'GSK3B', 'GSR', 'GSTM1', 'GTDC1', 'GTF2E2', 'GTF2H2', 'GTF2H3', 'GTF2I', 'GTPBP8', 'GUK1', 'HAT1', 'HAVCR1', 'HDHD1', 'HECW2', 'HES1', 'HIF1AN', 'HIGD2A', 'HIST1H2BD', 'HIST1H3A', 'HLA-A', 'HLA-DQB1', 'HLA-DRA', 'HLA-DRB1', 'HLA-DRB5', 'HMGCR', 'HMMR', 'HNF1B', 'HOXA5', 'HPRT1', 'HRSP12', 'HSD17B4', 'HSP90AA1', 'HSP90B1', 'HSPB1', 'HSPG2', 'HYAL2', 'IARS2', 'ID3', 'IDE', 'IDH3B', 'IFNG', 'IGF1', 'IGF1R', 'IGF2R', 'IKBKAP', 'IKZF5', 'IL11RA', 'IL13', 'IL1R1', 'IL2', 'IL27', 'ILK', 'IMPA2', 'INSIG1', 'INSR', 'IRS2', 'ISG20', 'ITGB3', 'JAK1', 'JAK3', 'JAZF1', 'JMJD6', 'KARS', 'KAT2B', 'KCNG1', 'KCNJ11', 'KDM1A', 'KIAA0100', 'KIAA0528', 'KIAA1009', 'KIAA1033', 'KIAA1279', 'KIF20A', 'KIF5C', 'KIF9', 'KIT', 'KLF3', 'KLHDC9', 'KLHL6', 'KLHL9', 'KSR2', 'KTN1', 'L1CAM', 'LASP1', 'LCK', 'LIG1', 'LIMK2', 'LNPEP', 'LPAR2', 'LPAR3', 'LRP10', 'LRPPRC', 'LRRC41', 'LRRK2', 'LSM5', 'LSM6', 'LSP1', 'LSS', 'LTA4H', 'MAP2K1', 'MAP2K2', 'MAP4K3', 'MAP4K4', 'MAPK14', 'MAPKAPK3', 'MARS', 'MASTL', 'MB', 'MBD6', 'MCCC1', 'MCM2', 'MCM6', 'MCTS1', 'MECOM', 'MED21', 'MEN1', 'MERTK', 'MESP1', 'METAP2', 'METRN', 'METTL14', 'MFSD10', 'MITF', 'MKI67IP', 'MKX', 'MLLT11', 'MMP2', 'MORF4L1', 'MORF4L2', 'MRPL12', 'MSH2', 'MSH6', 'MSRB2', 'MST1R', 'MTA1', 'MTA2', 'MTA3', 'MTFR1', 'MTOR', 'MTTP', 'MTX2', 'MUT', 'MXI1', 'MYL6', 'NAB2', 'NAGK', 'NARFL', 'NASP', 'NBN', 'NDUFA2', 'NDUFA4', 'NDUFA6', 'NDUFAF4', 'NDUFB5', 'NDUFC1', 'NDUFS3', 'NDUFS6', 'NDUFV2', 'NEK6', 'NEU1', 'NFAT5', 'NFE2L2', 'NFKB2', 'NGFR', 'NISCH', 'NNMT', 'NOD1', 'NOD2', 'NOL3', 'NOLC1', 'NOS3', 'NOSIP', 'NOTCH1', 'NOTCH2', 'NOTCH2NL', 'NR0B2', 'NR1H4', 'NR2C2', 'NRBP2', 'NRG1', 'NSDHL', 'NSF', 'NUDCD3', 'NUDT5', 'NUMA1', 'OXCT1', 'P4HB', 'PAFAH1B3', 'PAK1', 'PAK4', 'PANK4', 'PAX6', 'PCDH15', 'PCM1', 'PCNA', 'PCSK7', 'PDAP1', 'PDE4D', 'PDGFC', 'PDGFRB', 'PDHB', 'PDIA2', 'PDX1', 'PEX11A', 'PGAM1', 'PGD', 'PGK1', 'PHACTR1', 'PHF1', 'PHF10', 'PHF11', 'PHF15', 'PHF5A', 'PHKA1', 'PHLPP1', 'PIAS1', 'PIGK', 'PIH1D1', 'PIK3CB', 'PIK3CD', 'PIK3CG', 'PIK3R1', 'PIK3R3', 'PIP4K2B', 'PKIG', 'PKM2', 'PKN2', 'PLA2G12A', 'PLAGL1', 'PLCB3', 'PLCB4', 'PLCG2', 'PLEKHM1', 'PLK2', 'PLOD1', 'PLP2', 'PLSCR3', 'PMPCB', 'PMS1', 'PNN', 'PNP', 'POLR1C', 'POLR2C', 'POLR3C', 'POLR3F', 'PPAP2A', 'PPARG', 'PPARGC1A', 'PPARGC1B', 'PPIC', 'PPIF', 'PPP1R8', 'PPP2R5B', 'PPP3CA', 'PPT1', 'PPT2', 'PRDM4', 'PREX2', 'PRKAA1', 'PRKACA', 'PRKACG', 'PRKAG2', 'PRKAR1A', 'PRKCA', 'PRKCB', 'PRKCD', 'PRPF6', 'PRR7', 'PSAP', 'PSMA7', 'PSMD9', 'PSME1', 'PTCH1', 'PTGFR', 'PTK2', 'PTMS', 'PTPN1', 'PTPN6', 'PTTG1', 'PUS10', 'RAB1B', 'RAB31', 'RAD50', 'RAE1', 'RAI14', 'RALB', 'RANBP9', 'RAP1A', 'RARG', 'RARRES3', 'RASD1', 'RASSF1', 'RBCK1', 'RBM15', 'RDH14', 'REL', 'RELN', 'RERG', 'RET', 'RFC2', 'RGS2', 'RHOA', 'RICTOR', 'RIOK3', 'RNASET2', 'RNGTT', 'RNMT', 'RPA3', 'RPL22', 'RPS6KA2', 'RPS6KA4', 'RPTOR', 'RRAGC', 'RRM2', 'RRP1B', 'RTCD1', 'RUFY1', 'S100A6', 'SAP18', 'SARS', 'SAT1', 'SCAND1', 'SCARB1', 'SCRN1', 'SCYL3', 'SDHD', 'SEC24B', 'SENP5', 'SERPINA5', 'SERPINF2', 'SETD8', 'SFN', 'SHC1', 'SIAH1', 'SIRT3', 'SIRT5', 'SKAP2', 'SKP1', 'SLC16A3', 'SLC22A5', 'SLC25A10', 'SLC25A12', 'SLC25A4', 'SLC2A2', 'SLC36A1', 'SMAD1', 'SMAD5', 'SMARCA2', 'SMARCD2', 'SMC1A', 'SMC4', 'SMNDC1', 'SMYD3', 'SNAI2', 'SNCA', 'SNW1', 'SOAT2', 'SP3', 'SPAG4', 'SPTAN1', 'SPTLC2', 'SREBF2', 'SRSF4', 'SSBP2', 'ST14', 'ST7', 'STAM', 'STAT3', 'STK33', 'STK36', 'SUPT3H', 'SUPT4H1', 'SUPT5H', 'SYF2', 'SYNE2', 'TAAR8', 'TAB3', 'TAF8', 'TANK', 'TATDN2', 'TBC1D9B', 'TBK1', 'TBL3', 'TBPL1', 'TCEA2', 'TCERG1', 'TCF12', 'TCF7', 'TDRD3', 'TEAD1', 'TEK', 'TF', 'TFF1', 'TFPI', 'TFRC', 'TGFBR2', 'TH', 'THBD', 'THRAP3', 'TIMM17B', 'TIMM9', 'TIMP3', 'TIRAP', 'TKT', 'TLR4', 'TLR8', 'TM9SF3', 'TMEM11', 'TMEM174', 'TMEM97', 'TNFRSF10B', 'TNFRSF12A', 'TNFSF4', 'TNIK', 'TOMM22', 'TOP2B', 'TRAF5', 'TRAFD1', 'TRAPPC6A', 'TRIP13', 'TSC22D3', 'TSPAN3', 'TSPAN4', 'TSPAN6', 'TSSC4', 'TUBA1A', 'TUBB6', 'TWF2', 'TWIST1', 'TXNL1', 'TYMS', 'UBASH3A', 'UBE2C', 'UBE2Z', 'UGDH', 'UGP2', 'UIMC1', 'UMODL1', 'UQCR11', 'USP1', 'USP16', 'UXT', 'VAMP7', 'VDAC1', 'VGLL4', 'VHL', 'VPS26A', 'VPS8', 'VRK3', 'WDR6', 'WDR7', 'WNT1', 'WNT10B', 'WNT5A', 'WT1', 'WTAP', 'XPNPEP3', 'XRCC6', 'YAP1', 'YEATS4', 'YWHAH', 'YY1', 'ZAK', 'ZBTB25', 'ZBTB41', 'ZGPAT', 'ZIC2', 'ZNF131', 'ZNF219', 'ZNF281', 'ZNF296', 'ZNF300', 'ZNF331', 'ZNF426', 'ZNF451', 'ZNF561', 'ZNF572', 'ZNF597', 'ZNF599', 'ZNF626', 'ZNF662', 'ZNF669', 'ZNF671', 'ZNF692', 'ZNHIT3']	
10	['ABCC3', 'ACSL4', 'ACTN1', 'ADCK3', 'ADO', 'ANG', 'ANPEP', 'APOA5', 'ARNT', 'ASCL4', 'ASF1A', 'ASNA1', 'B4GALNT1', 'BCL9', 'BIRC3', 'C10ORF26', 'C1S', 'CAPNS1', 'CDH11', 'CHN1', 'CREBL2', 'CTCF', 'CTNND1', 'CTNS', 'CTRB1', 'CTRB2', 'CYP3A5', 'CYTH1', 'DKK1', 'DYNLT3', 'ECD', 'EDEM1', 'EFNB3', 'EIF2C1', 'ELAC2', 'ELTD1', 'ERBB2IP', 'ERGIC2', 'ETV1', 'FADS2', 'FBP1', 'FH', 'FMR1', 'FST', 'FURIN', 'G6PD', 'GART', 'GATA2', 'GCLC', 'GLRX2', 'GNB1', 'GPR119', 'GPR126', 'GPR139', 'GPR148', 'GPR31', 'GPR37', 'GPRC5C', 'GPSM1', 'H1FOO', 'HDAC10', 'HDAC6', 'HLA-DRB4', 'HMGN5', 'IKBKE', 'IL1B', 'IL2RA', 'IL4', 'INPPL1', 'IRF2', 'ISOC1', 'JOSD1', 'JUN', 'KAT6A', 'KAT6B', 'KDM4A', 'KEAP1', 'KLF10', 'LAP3', 'LHX2', 'LIG3', 'LPAR5', 'LRRC59', 'LRSAM1', 'MAN2A2', 'MAPK8', 'MAT2A', 'MCM5', 'MED6', 'MLL2', 'NADSYN1', 'NAGA', 'NAIP', 'NDRG1', 'NDUFA7', 'NDUFB4', 'NFIB', 'NFKBIB', 'NTRK1', 'NVL', 'OLIG3', 'OTUD7A', 'PAPD5', 'PBRM1', 'PDIA4', 'PHTF2', 'PLAT', 'PLK4', 'PLSCR1', 'PPFIBP2', 'PPIA', 'PRKAG1', 'PRKCQ', 'PRSS2', 'PSENEN', 'PTHLH', 'PTPLA', 'PXN', 'RAB11A', 'RAB23', 'RAC3', 'RASA1', 'RDX', 'RING1', 'RNF138', 'RORC', 'ROS1', 'RPS10', 'SATB1', 'SC5DL',	175

	'SERPINC1', 'SGK1', 'SIRT6', 'SLC16A5', 'SLC25A32', 'SLC35F2', 'SLC9A1', 'SLIRP', 'SLITRK6', 'SMG7', 'SNX6', 'SOX10', 'SPTLC1', 'SQRDL', 'SS18L1', 'SSB', 'STRADB', 'SUFU', 'SUPV3L1', 'TCF3', 'TCFL5', 'TESK1', 'TFCP2', 'TGFB1', 'THAP11', 'TIMELESS', 'TIMP2', 'TLR2', 'TLR9', 'TRIM16', 'TRIM38', 'TRRAP', 'TXN', 'TXNRD1', 'TYRO3', 'UBAC2', 'WDR67', 'YWHAQ', 'ZNF136', 'ZNF398', 'ZNF501', 'ZNF624', 'ZNF786', 'ZNF792', 'ZNF85']	
11	['ABCA3', 'ABCC5', 'ABHD2', 'ABHD6', 'ACBD3', 'ACTB', 'ACVR1B', 'ADCK4', 'ADCY3', 'ADPGK', 'ADRA2A', 'ADRA2C', 'AES', 'AFG3L2', 'AIMP2', 'AKAP8', 'ALDH1A1', 'ALDH3B1', 'ALDH7A1', 'ALK', 'ALPK3', 'AMDHD2', 'ANO1', 'AP2M1', 'APC', 'APOB', 'APOC2', 'APP', 'APRT', 'AQP12A', 'AR', 'ARHGAP9', 'ARHGEF5', 'ARPC5', 'ARPP19', 'ARRB1', 'ASNS', 'ASPM', 'ATP2C1', 'ATP5F1', 'ATP6V1A', 'ATP6V1F', 'ATRX', 'ATXN1', 'AURKB', 'AXIN2', 'AZI2', 'B4GALT3', 'BATF', 'BAX', 'BCL2L13', 'BLK', 'BLMH', 'BRD4', 'BRSK1', 'BTG1', 'BZW2', 'C16ORF80', 'C2CD2L', 'C9ORF96', 'CALM1', 'CAND1', 'CAPG', 'CAPN1', 'CAPNS2', 'CARD9', 'CASP6', 'CAST', 'CAV1', 'CBX4', 'CCNH', 'CCT7', 'CD24', 'CD59', 'CDC37', 'CDC42SE1', 'CDCA8', 'CDK19', 'CDK5R1', 'CDO1', 'CEBPZ', 'CEP55', 'CERS3', 'CFL1', 'CFTR', 'CGRRF1', 'CHD1', 'CHD8', 'CLCN3', 'CLIC4', 'CLK4', 'CLPB', 'CLTA', 'CLTB', 'COMT', 'COPA', 'COPB2', 'COPS7A', 'CROT', 'CTBP2', 'CTH', 'CTSH', 'CXXC4', 'CYC1', 'DACH2', 'DCAKD', 'DCP1A', 'DECR1', 'DHPS', 'DHRS4', 'DLC1', 'DLG3', 'DLGAP5', 'DLX3', 'DMRTB1', 'DNAJB2', 'DNMT1', 'DNTTIP2', 'DPP4', 'DPY30', 'DYNLL2', 'E2F4', 'EFCAB2', 'EGLN1', 'EIF1B', 'EIF2B3', 'EIF2B5', 'EIF2S2', 'EIF3J', 'EIF4A2', 'ELK1', 'EPHA1', 'EPHA8', 'ERBB2', 'ERCC6L', 'ERGIC1', 'ESR1', 'ETFA', 'ETFB', 'ETV5', 'F2R', 'FABP6', 'FAM114A2', 'FAM134C', 'FANCA', 'FAS', 'FBXL12', 'FBXO11', 'FDX1', 'FGF9', 'FGFR1', 'FGR', 'FKBP5', 'FOSL2', 'FOXA2', 'FOXO4', 'FSD1', 'FTSJ1', 'FZD1', 'FZD3', 'G6PC', 'GABRA1', 'GALC', 'GALK1', 'GBGT1', 'GFOD2', 'GFRA1', 'GGH', 'GGT1', 'GLA', 'GMEB1', 'GNA13', 'GNAI3', 'GNAZ', 'GNB2', 'GNG8', 'GOLT1B', 'GOT2', 'GPI', 'GPR107', 'GPR156', 'GPR174', 'GPR87', 'GPX7', 'GRHPR', 'GRN', 'GSC', 'GSTM2', 'GSTZ1', 'GUSB', 'HDAC1', 'HEATR1', 'HERPUD1', 'HFE', 'HIST1H2AC', 'HIST2H2BE', 'HN1L', 'HNF4G', 'HOMER2', 'HSPA1A', 'HSPA5', 'HSPB8', 'HSPE1', 'HTRA1', 'HYAL1', 'ICK', 'IDH3G', 'IGBP1', 'IGF2', 'IGF2BP1', 'IGFBP5', 'IKZF1', 'IL12A', 'ILF2', 'IP6K1', 'IRAK2', 'IRF9', 'IRS1', 'ITGB1BP1', 'ITK', 'JAG1', 'KAT7', 'KAZALD1', 'KCNQ1', 'KCTD2', 'KDM4B', 'KDM4C', 'KDM6B', 'KIF14', 'KIF5B', 'KLF2', 'KSR1', 'LGALS2', 'LIMK1', 'LONRF2', 'LOXL1', 'LPHN2', 'LRP4', 'LRRC32', 'LYN', 'LYPD3', 'MAP3K13', 'MAP3K15', 'MAP3K7', 'MAP3K9', 'MAP4K5', 'MAP7', 'MAPK12', 'MAPKAP1', 'MDM2', 'MED1', 'MED14', 'MED26', 'MED4', 'MEF2A', 'MEGF9', 'MEST', 'MEX3D', 'MGAT4A', 'MGLL', 'MKNK2', 'MLST8', 'MOS', 'MRGPRF', 'MRPL19', 'MRPS2', 'MSRA', 'MT1F', 'MYB', 'MYCN', 'MYL6B', 'MYLK3', 'NCOA3', 'NEK4', 'NENF', 'NME7', 'NMI', 'NMNAT2', 'NMRAL1', 'NMUR2', 'NPEPL1', 'NPM1', 'NPY1R', 'NR0B1', 'NR2F6', 'NRAS', 'NRIP1', 'NUAK1', 'NUP88', 'OBSCN', 'OGFOD1', 'OVOL2', 'OXA1L', 'OXER1', 'OXTR', 'P2RX4', 'PABPC1', 'PAK2', 'PARG', 'PARL', 'PAX8', 'PBX4', 'PC', 'PCBD1', 'PCMT1', 'PCSK9', 'PDCD1LG2', 'PDCD2', 'PDK1', 'PDK2', 'PDK3', 'PDK4', 'PDS5B', 'PDXP', 'PET112', 'PEX13', 'PFKFB1', 'PFKP', 'PFN2', 'PGK2', 'PHF13', 'PHF17', 'PHRF1', 'PI4KAP2', 'PIAS4', 'PIGB', 'PIK3R2', 'PIP4K2C', 'PIPOX', 'PJA1', 'PKD1', 'PKN3', 'PLK1', 'PLOD3', 'PLXNC1', 'PMAIP1', 'PMM2', 'POLR3E', 'POU2F2', 'PPIH', 'PPOX', 'PPP2CB', 'PPP2R1A', 'PPP3CB', 'PPP4C', 'PRCC', 'PRDX1', 'PRKAR2B', 'PRKG2', 'PRMT1', 'PRMT2', 'PROC', 'PROSC', 'PRPF4B', 'PSAT1', 'PSMD5', 'PTK7', 'PUM2', 'PVR', 'PYCRL', 'PYGO1', 'RAB5A', 'RAB7A', 'RAD51', 'RAD54L', 'RAD9A', 'RALA', 'RALGDS', 'RBKS', 'RBM8A', 'RETSAT', 'RFNG', 'RGS16', 'RGS19', 'RHOBTB1', 'RNF11', 'RNF123', 'RNF167', 'RNH1', 'RNPS1', 'ROR2', 'RPA1', 'RPA2', 'RPS6KA5', 'RPS6KA6', 'RPS6KC1', 'RPS6KL1', 'RRAGA', 'RXFP4', 'S100A13', 'S100A4', 'S100Z', 'SCAF8', 'SDF2L1', 'SDHB', 'SEC16A', 'SEC24C', 'SENP6', 'SETDB1', 'SGK3', 'SH3GL1', 'SHB', 'SHC4', 'SKP2', 'SLC22A23', 'SLC25A14', 'SLC25A5', 'SLC35A3', 'SLC35B1', 'SLC38A1', 'SMARCA5', 'SMARCAD1', 'SMOC2', 'SMS', 'SNAI1', 'SNRNP70', 'SNX13', 'SNX17', 'SOCS1', 'SOD2', 'SORBS3', 'SOS2', 'SOX3', 'SPIB', 'SRP14', 'SRRM1', 'SSBP4', 'STAT6', 'STX1A', 'STX4', 'SUCLA2', 'SUCLG1', 'SULT2B1', 'SUZ12', 'SV2A', 'SYPL1', 'SYT1', 'TBCB', 'TFDP1', 'TFDP2', 'TFE3', 'TIAM1', 'TLE3', 'TM9SF2', 'TMEM110', 'TMEM154', 'TNFSF15', 'TNFSF8', 'TOMM70A', 'TOR1A', 'TOX2', 'TPI1', 'TRAP1', 'TRIM13', 'TRIM2', 'TSC22D1', 'TSSK6', 'TTC3', 'TUBB2C', 'TXNL4B', 'UBA52', 'UBE2A', 'UBE2L3', 'UBE2L6', 'UBE2V1', 'UBE4A', 'UFD1L', 'USP14', 'USP47', 'USP6NL', 'UTP14A', 'UTP18', 'UTS2R', 'VPS28', 'VTI1A', 'WDR61', 'WEE1', 'WFS1', 'WIPF2', 'XBP1', 'XRCC5', 'YKT6', 'YTHDF1', 'YTHDF2', 'ZBTB46', 'ZER1', 'ZFP36', 'ZFP36L2', 'ZNF138', 'ZNF174', 'ZNF350', 'ZNF418', 'ZNF432', 'ZNF433', 'ZNF434', 'ZNF488', 'ZNF543', 'ZNF581', 'ZNF589', 'ZNF607', 'ZNF608', 'ZNF610', 'ZNF668', 'ZNF673', 'ZNF678', 'ZNF785', 'ZNF791', 'ZSCAN1']	508
12	['AMHR2', 'C21ORF7', 'CCL4', 'CDC42BPG', 'CDK17', 'CSF1R', 'HCK', 'LOC388259', 'LOC441655', 'MARK2', 'NPR1', 'PHKG1', 'PLXNB2', 'SRPK2', 'TAF1']	15
13	['ACVRL1', 'ANKK1', 'CAMK2A', 'CDC42BPB', 'CDK11A', 'CDK15', 'CDK20', 'CDKL2', 'EPHA7', 'FASTK', 'FBXL4', 'FN3KRP', 'GRK4', 'GTF2H1', 'GUCY2D', 'KIAA1804', 'LMTK3', 'MEX3B', 'MPP4', 'MUSK', 'MYLK4', 'PFKM', 'PIP4K2A', 'PSKH1', 'SMG1', 'SRPK3', 'STK32B', 'TLK1', 'WNK1', 'XYLB']	30
14	['ACVR2B', 'ADRBK2', 'AGK', 'BMP2KL', 'DCLK3', 'DGKK', 'FUK', 'LOC392226', 'LOC392347', 'LRRK1', 'MARK2P10', 'SH3BP4']	12
15	['AK8', 'CDK5R2', 'DCLK2', 'GLYCTK', 'LOC391295', 'TTN']	6
16	['AAK1', 'ACVR2A', 'ADCK5', 'ALPK2', 'ARSG', 'BMP2K', 'C9ORF95', 'CAMKV', 'CDK12', 'CDKL1', 'CIB1', 'COL4A3BP', 'DAK', 'DGKB', 'DGUOK', 'DMPK', 'EPHA10', 'EPHA5', 'EPHB1', 'ERN2', 'FASTKD2', 'GK2', 'HIPK2', 'HIPK3', 'IP6K3', 'KHK', 'LOC442075', 'LTK', 'MAGI1', 'MAP2K7', 'MAPK10', 'MAPK11', 'MARK1', 'MARK3', 'MLKL', 'NADK', 'NIM1', 'NME6', 'NUAK2', 'PFKFB4', 'PI4K2A', 'PINK1', 'PIP5K1B', 'PIP5KL1', 'PLXNA4', 'PLXNB3', 'PLXND1', 'PMVK', 'SBK1', 'SLAMF6', 'SPEG', 'STK17B', 'STK32A', 'STK32C', 'STK35', 'STK40', 'TEC', 'TIE1', 'TJP2', 'TPK1', 'TSSK1B', 'TSSK4']	62
17	['GEMIN2', 'KIF13B', 'PPP2R5C']	3

18	['ACADS', 'ACY1', 'ADAM17', 'AKR1B1', 'AMIGO3', 'AP2A2', 'APOA1', 'APOH', 'ARFIP2', 'ARG2', 'ARPC3', 'ASGR2', 'ASRGL1', 'ATP2A2', 'ATP5B', 'ATP6V0C', 'BBC3', 'BHMT2', 'BRAP', 'BRPF3', 'CASP7', 'CBFA2T3', 'CCNE2', 'CCT8', 'CDC42', 'CDK8', 'CHIC2', 'CLCN5', 'CLPP', 'CNOT7', 'CORO1A', 'CREB3L4', 'CSAD', 'CSNK1G2', 'CSNK1G3', 'CTDSP1', 'CTNNAL1', 'CTPS', 'CYB5B', 'CYP1A1', 'DARS', 'DCLRE1B', 'DDIT4', 'DGKD', 'DLX2', 'DROSHA', 'DYNLL1', 'DYRK3', 'EPC1', 'ERH', 'FANCF', 'FGFR2', 'FIGF', 'FKBP3', 'FLT4', 'FRAT1', 'FZD4', 'GAA', 'GABBR2', 'GATA1', 'GCA', 'GDI1', 'GIT1', 'GMDS', 'GNA11', 'GNAI1', 'GNG5', 'GPBAR1', 'GPR101', 'GPR110', 'GPR111', 'GPR113', 'GPR153', 'GPR26', 'GPR55', 'GPR56', 'GPR84', 'GPX2', 'GREB1', 'HAL', 'HCFC2', 'HHEX', 'HIPK1', 'HLA-DQA1', 'HLA-DRB3', 'HMGB3', 'HMGN4', 'HMOX2', 'HOXA1', 'HOXB5', 'ICAM1', 'IDH3A', 'IGFBP2', 'IL10RB', 'IL15', 'ING3', 'IRF4', 'ITCH', 'ITFG1', 'KAT5', 'KIAA0355', 'KIF11', 'KNDC1', 'KNG1', 'KRT25', 'LGR6', 'LIPH', 'LTBR', 'MAFG', 'ME1', 'ME3', 'METTL4', 'MLLT6', 'MLXIP', 'MPI', 'MSH5', 'MSL3', 'MYC', 'NME4', 'NME9', 'NPBWR1', 'NPRL2', 'NPTN', 'NQO2', 'NRIP3', 'NUCKS1', 'OAZ2', 'OPN4', 'OR5W2', 'PDHA1', 'PDPK1', 'PFDN2', 'PGBD1', 'PHF21B', 'PLA2G4A', 'PLD1', 'PNPO', 'POLR2E', 'POLR2F', 'POLR3K', 'PON3', 'PPP2R4', 'PPP3R2', 'PRDM7', 'PRKD2', 'PRNP', 'PROX1', 'PSEN1', 'PSEN2', 'PSMA5', 'PTGS2', 'PTP4A1', 'PTRF', 'QPCT', 'RAF1', 'RBMX', 'RBX1', 'RFWD2', 'RFX4', 'RGS18', 'RHOB', 'RIPK1', 'RIT1', 'RNF14', 'RNF166', 'RPN1', 'RTEL1', 'SEC24D', 'SERPINA1', 'SKIV2L', 'SLC25A1', 'SLC25A24', 'SLC39A6', 'SLC7A9', 'SMAD2', 'SMAD4', 'SMOX', 'SNRPA', 'SPIC', 'SPRY2', 'STIL', 'STRN4', 'SUPT16H', 'TACC3', 'TARS', 'TBC1D2B', 'TBX20', 'TERF1', 'TIMP4', 'TMEM109', 'TNFRSF11A', 'TNFRSF14', 'TNFRSF18', 'TNIP1', 'TP53RK', 'TRIM29', 'TRIO', 'TRPM2', 'TRPS1', 'TTR', 'UBE3A', 'UBE3B', 'UGT1A1', 'UGT1A3', 'ULK1', 'UQCRC1', 'USP32', 'VIM', 'VPS72', 'WNT7B', 'YAF2', 'ZBTB45', 'ZBTB49', 'ZEB1', 'ZFP112', 'ZFP3', 'ZFP36L1', 'ZMYND11', 'ZNF182', 'ZNF232', 'ZNF317', 'ZNF324', 'ZNF385B', 'ZNF517', 'ZNF524', 'ZNF562', 'ZNF567', 'ZNF596', 'ZNF623', 'ZNF684', 'ZRANB2']	231
19	['PSMA1', 'PSMA3', 'PSMB1', 'PSMB2', 'PSMB5', 'PSMB7', 'PSMD1', 'PSMD2', 'PSMD3', 'PSMD4', 'VCP']	11
20	['ACSL6', 'CHD4', 'DISC1', 'KCNA2', 'MED11', 'NPAS3', 'NRXN1', 'PHF20L1', 'PTPN5', 'QKI', 'RBFOX3', 'RCOR1', 'SHANK2', 'SYNE1', 'WIF1']	15
21	['ACAT2', 'AGL', 'AKR1A1', 'ALDOA', 'ARF4', 'ARL1', 'ARNTL', 'ATF6', 'BCCIP', 'BMPR1B', 'C19ORF6', 'CAMKK2', 'CCNA1', 'CD3D', 'CD70', 'CDK11B', 'CNOT8', 'COPZ1', 'CTNNBIP1', 'CXCL1', 'DDX49', 'DHRS2', 'ECSIT', 'EXOSC4', 'EXOSC8', 'FADS1', 'FAM102A', 'FGG', 'FZD7', 'GATAD1', 'GBP2', 'GMPS', 'GPR176', 'GPR39', 'GPR78', 'GPX4', 'GYG1', 'HEXB', 'HIST1H1B', 'HIST1H1E', 'HLA-DMB', 'HMGB1', 'HPD', 'IL2RB', 'INPP5D', 'ISX', 'ITGA2', 'KREMEN1', 'LIAS', 'LMO4', 'MACF1', 'MAP2K6', 'MATN3', 'MCM8', 'MDH2', 'MTHFD2', 'NDUFA1', 'NDUFC2', 'NKX2-3', 'NT5C2', 'NXF1', 'OGG1', 'OPA1', 'PAPOLA', 'PFKFB3', 'PHF23', 'PHYH', 'PISD', 'PJA2', 'POFUT1', 'PPAP2B', 'PROCR', 'PRSS42', 'QPRT', 'RASSF2', 'RAVER1', 'RBP1', 'RBPJ', 'RNF125', 'RPS16', 'RPS3', 'RPS3A', 'RPS5', 'RUVBL2', 'RXRG', 'SDHA', 'SF3B1', 'SIM2', 'SLC16A1', 'SOX2', 'SPP1', 'STMN1', 'TAB2', 'TBC1D15', 'TSHZ3', 'TSTA3', 'TUBD1', 'UBC', 'ULK3', 'UROD', 'VAMP3', 'WARS2', 'ZBTB20', 'ZBTB48', 'ZFAND6', 'ZIM3', 'ZNF22', 'ZNF263', 'ZNF366', 'ZNF658', 'ZNF717', 'ZNF8']	112
22	['AATF', 'ABL2', 'ACO2', 'AKR1C3', 'ALAS2', 'ALDOC', 'ANKRD49', 'ANO10', 'AOC3', 'ARHGAP35', 'ARL3', 'ATP11B', 'ATP5D', 'AURKA', 'B4GALT1', 'B4GALT4', 'BBS9', 'BID', 'BIRC5', 'BLM', 'BLVRA', 'C14ORF181', 'C1QTNF6', 'CAMK2G', 'CAMSAP2', 'CAT', 'CBR3', 'CBS', 'CCNC', 'CD2BP2', 'CD83', 'CDC25A', 'CENPE', 'CHRAC1', 'COG4', 'CRKL', 'CSNK1E', 'CSRP1', 'DCPS', 'DEK', 'DHRS7', 'DHX29', 'DNAJB9', 'DNAJC1', 'DUT', 'E2F5', 'EIF3H', 'ENPP1', 'ENTPD6', 'EP400', 'EPB41L2', 'ERAP1', 'ESPL1', 'ESR2', 'ETNK1', 'EZH2', 'FASLG', 'FDX1L', 'FECH', 'FOSL1', 'GLI3', 'HADH', 'HDAC3', 'HDGFRP3', 'HGS', 'HIST1H2BK', 'HMGB4', 'HMGCS1', 'HPGD', 'HRAS', 'HSF5', 'HSPA9', 'HYOU1', 'IL23R', 'IL6ST', 'IQGAP1', 'ISL1', 'ITGAV', 'ITPKB', 'ITPR1', 'KCNK1', 'KCNK2', 'KDELR3', 'KDR', 'KIAA0196', 'KLHDC2', 'KRAS', 'KRT19', 'LARS', 'LDHB', 'LPXN', 'MAP2K5', 'MAPKAPK5', 'MBTPS1', 'MCM3', 'MGST2', 'MLEC', 'MLL', 'MSMO1', 'MTF2', 'MYD88', 'MYO10', 'NAB1', 'NANOG', 'NDUFA5', 'NEK7', 'NOX1', 'NPC1', 'NQO1', 'NR3C1', 'NRBP1', 'NUCB2', 'NUDT6', 'NUDT9', 'NUP133', 'NUP93', 'ORC1', 'P4HA1', 'P4HTM', 'PAF1', 'PARN', 'PARP4', 'PAX5', 'PDS5A', 'PHF2', 'PIK3R4', 'PIM1', 'PMS2P3', 'POLR2A', 'POLR2D', 'POLR2H', 'POLR2I', 'POP4', 'PPAT', 'PRCP', 'PRR15L', 'PSPH', 'PUS7L', 'QRSL1', 'RAN', 'RBM14', 'RBM34', 'RFK', 'RFX5', 'RIPK3', 'RNF186', 'RPL7', 'RPS13', 'RPS14', 'RPS15A', 'RPS19', 'RPS27A', 'RPS6', 'RPS7', 'RPS9', 'RPTN', 'RRM1', 'RRS1', 'RUVBL1', 'RXRA', 'SCNN1A', 'SFPQ', 'SLC25A28', 'SLC25A6', 'SMAD7', 'SMARCC1', 'SNAPC1', 'SNF8', 'SNRK', 'SNRPD1', 'SOAT1', 'SOD1', 'SPAG7', 'SRRT', 'STAM2', 'STAT1', 'STK3', 'STK39', 'TCIRG1', 'TGDS', 'THADA', 'TIPARP', 'TK1', 'TLE1', 'TLR5', 'TLR7', 'TMED10', 'TMEM2', 'TMEM5', 'TOP1', 'TPD52L2', 'TUBB2A', 'UBB', 'UBE3C', 'USP7', 'VRK1', 'VRK2', 'WHSC1', 'WRN', 'WWOX', 'XIAP', 'YARS', 'YME1L1', 'ZFAT', 'ZFX', 'ZFYVE19', 'ZMIZ2', 'ZNF202', 'ZNF318', 'ZNF449', 'ZNF629', 'ZNF768', 'ZPBP2']	213

Supplementary Table S4. Leaf nodes belonging to the clusters formed by the hierarchical clustering for overexpressed genes. 'Cluster node id' corresponds to the cluster node ids in the Supplementary Fig. S2 (B). The nodes of the clusters are listed in the 'Overexpressed genes' column. The number of over-expressed genes belonging to the clusters are shown in the 'Count' column represents.

Cluster node id	Overexpressed genes	Count
1	['KSR', 'MAP4K4']	2
2	['CAMKV', 'MAP4K2', 'PDIK1L', 'PIM3', 'PRKCG', 'PTK2B', 'RPS6KA2', 'SGK2']	8
3	['ACVR2B', 'ALPK1', 'CSF1R', 'GSK3A']	4
4	['FBXL10', 'HDAC6', 'POLD1', 'PRMT7', 'SETMAR', 'SIRT1', 'SMYD4']	7
5	['ABHD4', 'ACADM', 'ACADS', 'ACLY', 'ACTB', 'ACTL6A', 'ACTN2', 'ACTR3', 'ADAT1', 'ADC', 'ADCK3', 'ADORA2B', 'ADRBK1', 'ADSS', 'AGPAT1', 'AGPAT2', 'AGT', 'AHDC1', 'AIFM1', 'AKR1A1', 'AKTIP', 'ALDH1B1', 'ALDOA', 'ALG5', 'ALG9', 'AMHR2', 'AMIGO3', 'ANG', 'ANKRD49', 'ANXA5', 'AOF2', 'APEX1', 'APOBEC3H', 'APOC2', 'APOC3', 'APOH', 'APRT', 'ARFIP2', 'ARG2', 'ARID3A', 'ASAP2', 'ASCC3', 'ASCL2', 'ASCL4', 'ASF1A', 'ASF1B', 'ASGR2', 'ASMTL', 'ASNS', 'ASPH', 'ATAD1', 'ATF3', 'ATG16L1', 'ATG3', 'ATG7', 'ATOX1', 'ATP1B1', 'ATP5C1', 'ATP5L', 'ATP6V0A1', 'ATP6V0B', 'ATP6V1F', 'AXL', 'B3GNT1', 'B4GALT4', 'BACH1', 'BAD', 'BAMBI', 'BCAT2', 'BCCIP', 'BCL10', 'BCL2L11', 'BCL2L2', 'BCR-ABL', 'BCR-ABL_A269V', 'BCR-ABL_E285K', 'BCR-ABL_E316D', 'BCR-ABL_E499K', 'BCR-ABL_F317L', 'BCR-ABL_F486S', 'BCR-ABL_G321D', 'BCR-ABL_I352K', 'BCR-ABL_I502M', 'BCR-ABL_L248R', 'BCR-ABL_Q252H', 'BCR-ABL_T224A', 'BCR-ABL_T315I', 'BCR-ABL_Y253H', 'BHMT', 'BNIP1', 'BNIP3', 'BOLA1', 'BPHL', 'BUB3', 'BUD31', 'C16ORF5', 'C1QA', 'C2', 'CALCOCO2', 'CALM1', 'CAMK2G', 'CAMKK1', 'CANT1', 'CAPN1', 'CASD1', 'CASP14', 'CASP2', 'CBX6', 'CBX8', 'CCDC90A', 'CCDC92', 'CCND3', 'CCNL1', 'CD14', 'CD2BP2', 'CD40', 'CD44', 'CDC42SE1', 'CDC45', 'CDKN1A', 'CDKN1B', 'CDKN2C', 'CEBPE', 'CEBPG', 'CEPT1', 'CFL1', 'CHCHD7', 'CHMP4A', 'CIAPIN1', 'CIRBP', 'CISD1', 'CLK1', 'CLK3', 'CLPB', 'CLTA', 'CNDP2', 'CNOT3', 'CNOT4', 'COG7', 'COMT', 'COX5B', 'COX7B', 'CROT', 'CRX', 'CRY1', 'CRYAA', 'CSK', 'CSNK2A2', 'CTSD', 'CTSL1', 'CUL3', 'CXCL1', 'CXCL2', 'CYB5A', 'CYC1', 'CYP24A1', 'DCK', 'DCLRE1B', 'DCXR', 'DDAH2', 'DENND2D', 'DEPDC1', 'DHRS3', 'DHX29', 'DIABLO', 'DLC1', 'DLX1', 'DLX3', 'DLX4', 'DMD', 'DMRT1', 'DNAJA3', 'DPYD', 'DUSP14', 'DUSP22', 'DUSP28', 'DUSP6', 'DYNLL1', 'DYNLL2', 'DYRK1B', 'E2F8', 'E4F1', 'EAPP', 'ECH1', 'ECHS1', 'EDNRB', 'EFNB2', 'EHHADH', 'EIF1B', 'EIF2AK4', 'EIF3H', 'EIF4E', 'EIF4EBP1', 'EIF4EBP2', 'EIF4EBP3', 'ELAC2', 'ELOVL6', 'EPHA2', 'EPHB6', 'ERAP1', 'ERF', 'ESR1', 'ESRRG', 'ETFA', 'ETV6', 'EXOC2', 'EXOC6', 'EXT1', 'EXT2', 'F2R', 'FABP1', 'FABP4', 'FAIM', 'FAM127A', 'FAM3C', 'FBP1', 'FBXL12', 'FBXO5', 'FBXW7', 'FDFT1', 'FDX1', 'FECH', 'FEN1', 'FER', 'FGA', 'FGFR1OP', 'FOSL2', 'FOXM1', 'FOXP2', 'FTH1', 'FTSJ1', 'FYN', 'G2E3', 'G6PC', 'GABPB1', 'GALT', 'GATA2', 'GATA3', 'GCAT', 'GCLM', 'GDPD5', 'GGCX', 'GGH', 'GGPS1', 'GLDC', 'GLI1', 'GLRX', 'GMDS', 'GMEB1', 'GNAS', 'GNB1L', 'GNG4', 'GPC1', 'GPR146', 'GPR26', 'GPR65', 'GPRC5B', 'GRK6', 'GS1-115G20.2', 'GSTM1', 'GSTM2', 'GTF2A1', 'GTF2B', 'GTF2H3', 'GTF2IRD2', 'H2AFY', 'HAVCR1', 'HDAC1', 'HDAC10', 'HDAC3', 'HDAC8', 'HERPUD1', 'HES2', 'HEXIM1', 'HEY1', 'HEY2', 'HIF1A', 'HIPK4', 'HLA-A', 'HLA-DRA', 'HLA-DRB3', 'HLA-DRB4', 'HLF', 'HMGCR', 'HMGN3', 'HMGN5', 'HMMR', 'HMOX1', 'HNF4A', 'HOXA1', 'HOXA6', 'HOXA9', 'HOXC11', 'HOXC9', 'HOXD9', 'HPD', 'HPN', 'HSBP1', 'HSD17B7', 'HSD17B8', 'HSP90AB1', 'HSPA14', 'HSPA8', 'HSPB8', 'HSPB9', 'HUS1', 'HYAL2', 'ID2', 'IDE', 'IDH2', 'IFIH1', 'IFNA10', 'IFNAR2', 'IFNB1', 'IFNG', 'IFNGR1', 'IGFBP4', 'IGFBP5', 'IGHA2', 'IGHMBP2', 'IKZF2', 'IL13', 'IL13RA2', 'IL18', 'IL1R1', 'IL2', 'IL6R', 'IMPA1', 'IMPDH1', 'INHBE', 'INTS12', 'IP6K1', 'IP6K2', 'IRAK2', 'IRF5', 'ISX', 'ITGB2', 'ITPK1', 'JMJD1A', 'JUN', 'JUNB', 'KAT7', 'KBTBD4', 'KCNK2', 'KCNN4', 'KCTD17', 'KIAA0494', 'KIF20A', 'KLF6', 'KLHDC2', 'KLHDC9', 'KLHL3', 'KLK6', 'KLRC1', 'KNG1', 'KRT26', 'LANCL1', 'LCMT1', 'LCN2', 'LGMN', 'LIG1', 'LIPA', 'LIPT1', 'LPAR2', 'LPGAT1', 'LPL', 'LRRC32', 'LSP1', 'LSS', 'LYPD3', 'LYZ', 'LZIC', 'M6PR', 'MAFB', 'MAFG', 'MAP2K5', 'MAP3K6', 'MAPK13', 'MAPK14', 'MAPK8', 'MAPKAPK3', 'MAPKAPK5', 'MAST1', 'MAT1A', 'MAT2A', 'MAT2B', 'MAX', 'MB', 'MBNL1', 'MCTS1', 'MDH1', 'MDM4', 'MED6', 'MEOX2', 'MEST', 'METTL4', 'MKRN3', 'MLLT6', 'MLST8', 'MNAT1', 'MORF4L2', 'MOS', 'MRGPRX4', 'MRPL18', 'MRPS16', 'MRPS2', 'MSRA', 'MSX2', 'MTAP', 'MYF6', 'MYOD1', 'NANOG', 'NAP1L3', 'NCF2', 'NDUFA1', 'NDUFA7', 'NDUFA8', 'NDUFAB1', 'NDUFB3', 'NDUFB5', 'NDUFB6', 'NDUFB8', 'NDUFS4', 'NDUFS6', 'NDUFS7', 'NEK2', 'NEK9', 'NENF', 'NFKBIA', 'NFKBIB', 'NFYA', 'NFYB', 'NME1', 'NOV', 'NPDC1', 'NPSR1', 'NR2C2', 'NR2E1', 'NR3C1', 'NR4A1', 'NR4A2', 'NSA2', 'NSFL1C', 'NT5E', 'NUDT9', 'O3FAR1', 'OAS1', 'OPN3', 'OPN5', 'OR52B2', 'OSGEP', 'OTX2', 'OVCA2', 'P2RX4', 'P2RY12', 'P2RY2', 'PAFAH1B1', 'PAFAH1B2', 'PAPD7', 'PAQR8', 'PARL', 'PARN', 'PBX4', 'PBXIP1', 'PCGF5', 'PDAP1', 'PDGFRA', 'PDHB', 'PDHX', 'PDK1', 'PDLIM4', 'PDPK1', 'PEX13', 'PEX19', 'PGK1', 'PHB2', 'PHF1', 'PHTF2', 'PIH1D1', 'PINK1', 'PISD', 'PKIG', 'PLAT', 'PLCG2', 'PLEKHG5', 'PLP2', 'PMF1-BGLAP', 'PNPO', 'POLB', 'POLE3', 'POLR1C', 'POLR2D', 'POLR2H', 'POLR3K', 'POU5F1', 'PPARA', 'PPARD', 'PPP2CB', 'PRAME', 'PRCC', 'PRDM1', 'PRDM7', 'PRDX2', 'PRDX5', 'PRKAB2', 'PRKAR1A', 'PRMT2', 'PROCR', 'PROKR1', 'PRPF4B', 'PRSS23', 'PSAT1', 'PSMA5', 'PSME1', 'PTPN12', 'PTPN2', 'PTPN4', 'PTPN6', 'PYCR1', 'QDPR', 'QPRT', 'QRSL1', 'RAB11FIP3', 'RAB23', 'RAB27A', 'RABGGTA', 'RAD1', 'RAGE', 'RALBP1', 'RAPSN', 'RARA', 'RARG', 'RASA1', 'RBKS', 'RC3H2', 'RCHY1', 'RDBP', 'RELA', 'RET', 'RGS3', 'RGS4', 'RHOA', 'RIPK1', 'RIPK2', 'RIT1', 'RNASE4', 'RNF11', 'RORC', 'RPA2', 'RPF1', 'RPL19', 'RPN1', 'RPS10', 'RPS3A', 'RPS6', 'RSU1', 'RTCD1', 'RTN4RL1', 'RXFP1', 'S100A11', 'S100B', 'S1PR3', 'SACM1L',	740

	'SAT1', 'SATB2', 'SCMH1', 'SCP2', 'SCRN1', 'SDF2L1', 'SDHC', 'SENP2', 'SENP5', 'SERPINA12', 'SERPINA5', 'SET', 'SETD6', 'SETD8', 'SF3A3', 'SGK1', 'SGPL1', 'SHC4', 'SHOX2', 'SIAH1', 'SIRT6', 'SKP1', 'SLC25A10', 'SLC2A6', 'SLC30A2', 'SLC38A2', 'SLC3A2', 'SLC4A2', 'SLC6A14', 'SMAD2', 'SMAD3', 'SMNDC1', 'SNAI3', 'SNRK', 'SNRNP70', 'SNX17', 'SOX10', 'SOX14', 'SOX2', 'SP8', 'SPAG7', 'SPARC', 'SPP1', 'SPRY1', 'SPTLC2', 'SRD5A1', 'SRM', 'SRP14', 'STAT3', 'STAT4', 'STK25', 'STK38', 'STUB1', 'STYK1', 'SUCLA2', 'SULT1A2', 'SUV39H2', 'SUZ12', 'SYNGR3', 'SYT1', 'TAF12', 'TAF13', 'TAP1', 'TBCB', 'TCF4', 'TCIRG1', 'TERF1', 'TEX10', 'TFAP2A', 'TFAP4', 'TFF2', 'TFG', 'TH', 'TIMELESS', 'TIRAP', 'TKT', 'TLE1', 'TLR2', 'TLX2', 'TMED10', 'TNF', 'TNFRSF19', 'TNFRSF1A', 'TNFRSF21', 'TNFSF8', 'TOLLIP', 'TOMM34', 'TPM1', 'TRAF2', 'TRAF4', 'TRAP1', 'TRIM13', 'TRIM27', 'TRIM29', 'TRIP10', 'TSC2', 'TSC22D1', 'TSC22D3', 'TSG101', 'TSN', 'TTK', 'TTR', 'TUBB3', 'TUBB6', 'TUBD1', 'TWF2', 'TXLNA', 'TXNL4B', 'UBE2D1', 'UBE2J1', 'UBE2V1', 'UBL5', 'UBOX5', 'UGT1A1', 'UGT1A6', 'UQCR11', 'USP14', 'USP6NL', 'VAMP3', 'VEGFA', 'VEGFB', 'VIM', 'VN1R5', 'VPS26A', 'VRK1', 'WARS2', 'WDR61', 'WFDC2', 'WTAP', 'XBP1', 'XPA', 'XPO7', 'ZBED1', 'ZBP1', 'ZBTB1', 'ZBTB26', 'ZBTB43', 'ZER1', 'ZFAND6', 'ZFP112', 'ZFP36L1', 'ZFP91', 'ZFPM2', 'ZFYVE19', 'ZGPAT', 'ZHX1', 'ZNF134', 'ZNF136', 'ZNF148', 'ZNF169', 'ZNF212', 'ZNF238', 'ZNF253', 'ZNF281', 'ZNF285A', 'ZNF3', 'ZNF317', 'ZNF32', 'ZNF345', 'ZNF366', 'ZNF396', 'ZNF444', 'ZNF496', 'ZNF502', 'ZNF559', 'ZNF597', 'ZNF610', 'ZNF645', 'ZNF692', 'ZNF785', 'ZNF791', 'ZNF8', 'ZNF92', 'ZNHIT3', 'ZPBP2', 'ZRANB2', 'ZSCAN21']	
6	['CDX2', 'EBF1', 'ELK1', 'ELK3', 'ERG', 'FADD', 'FIS1', 'FLI1', 'GADD45A', 'GADD45B', 'GCK', 'HOXB13', 'HRSP12', 'HTR2C', 'IL2RB', 'KCNA3', 'KCNC1', 'KLF3', 'LTBR', 'MAGEB6', 'MAP2K6', 'NDUFA13', 'OVOL2', 'PHB', 'SLC37A4', 'TBC1D3', 'TNFRSF10B', 'UGCG', 'VDAC1', 'VGLL4', 'ZNF296']	31
7	['ABTB1', 'ACVR1C', 'ACVR2A', 'ARNTL2', 'ATOH1', 'BCR-ABL_D276V', 'BCR-ABL_E236K', 'BCR-ABL_E279K', 'BCR-ABL_E281K', 'BCR-ABL_E286K', 'BCR-ABL_E292Q', 'BCR-ABL_G250E', 'BCR-ABL_G372R', 'BCR-ABL_H396P', 'BCR-ABL_L451M', 'BCR-ABL_M244I', 'BCR-ABL_M278K', 'BCR-ABL_N374S', 'BCR-ABL_Q300H', 'BCR-ABL_V339A', 'BRD9', 'C20orf194', 'CAMKK2', 'CCNF', 'CHD2', 'CREB3L2', 'CSDA', 'DACH1', 'DACH2', 'DCLK2', 'DNAJC1', 'DNAJC2', 'DUS3L', 'DVL1', 'DYRK4', 'EGR2', 'ELF3', 'ELK4', 'EPHB1', 'ESRRA', 'ESYT1', 'ETV7', 'FARSB', 'FASTK', 'FBXL13', 'FLT4', 'FOXR2', 'FRK', 'GCM1', 'GMCL1', 'GOLGA2', 'GPATCH8', 'GRK4', 'HMBOX1', 'HSPBAP1', 'INSRR', 'JMJD1B', 'KCMF1', 'KCNS3', 'KLHL32', 'LASS2', 'LASS3', 'LASS4', 'LRP1', 'MAP2K3', 'MAP3K13', 'MATK', 'MBD3', 'MBNL3', 'MEOX1', 'MLL5', 'MNT', 'MPO', 'MRPS17', 'MRPS31', 'MXRA8', 'MYLK3', 'MYLK4', 'MYO3B', 'MYT1', 'NCOA2', 'NEK11', 'NPR2', 'NR1H4', 'NR1I3', 'NR5A1', 'NTRK1', 'PADI4', 'PCGF6', 'PIP5K3', 'PKNOX1', 'PPP1R10', 'RABGEF1', 'RB1', 'RBM14', 'REL', 'RELL2', 'REPIN1', 'RGS7', 'RGS9', 'RIPK5', 'SMARCA5', 'SMARCD1', 'SMYD1', 'SNRNP25', 'TBX22', 'TCEB3B', 'THAP8', 'TRAIP', 'TRIM21', 'TRIM62', 'TSSK2', 'USF2', 'USP39', 'WHSC1L1', 'WNK1', 'ZBED2', 'ZBTB37', 'ZBTB38', 'ZBTB46', 'ZBTB7A', 'ZBTB7B', 'ZC3H7B', 'ZDHHC11', 'ZIC3', 'ZKSCAN4', 'ZNF100', 'ZNF187', 'ZNF19', 'ZNF193', 'ZNF205', 'ZNF239', 'ZNF248', 'ZNF257', 'ZNF329', 'ZNF354A', 'ZNF37A', 'ZNF384', 'ZNF385A', 'ZNF433', 'ZNF439', 'ZNF461', 'ZNF486', 'ZNF500', 'ZNF518A', 'ZNF557', 'ZNF607', 'ZNF649', 'ZNF658', 'ZNF671', 'ZNF70', 'ZNF701', 'ZNF808', 'ZRSR1', 'ZSCAN12', 'ZSCAN5A', 'ZXDC']	157
8	['AFF1', 'ATF1', 'CAMK4', 'DCAMKL2', 'DKFZP761P0423', 'DLX6', 'FOXD4L6', 'HINFP', 'KCNS2', 'PRKY', 'RFX3', 'RXRB', 'SP110', 'STK32C', 'THAP11', 'TYK2', 'ZNF322A']	17
9	['ALPK2', 'ANAPC2', 'ARMC5', 'BARD1', 'CAMK2A', 'CDK3', 'DHX57', 'DMRT3', 'E2F3', 'FAM171B', 'FBXO36', 'FBXO38', 'FLJ25006', 'GATAD2A', 'GON4L', 'GTF2H2', 'KCTD3', 'KIAA1683', 'KLF11', 'KLF9', 'LARP2', 'LCORL', 'MAP4K5', 'MEF2C', 'MESP1', 'POGZ', 'PRDM5', 'PRKD1', 'RAPGEF3', 'RFWD3', 'RGS6', 'RNF10', 'SOX15', 'SOX5', 'SRPK2', 'TAF6L', 'TBX3', 'TCEB3', 'TLX3', 'TMPO', 'TRAF5', 'TRIM42', 'TRIP12', 'TSSK3', 'UHRF1', 'ZBTB17', 'ZBTB40', 'ZBTB44', 'ZNF323', 'ZNF483', 'ZNF512', 'ZNF530', 'ZNF627', 'ZNF770']	54
10	['C17ORF49', 'EGR1', 'HP1BP3', 'KLHL29', 'KRTAP5-9', 'NRF1', 'PLXNA4', 'PSMD12', 'RAPGEF5', 'TADA2L', 'THRB', 'ZFP2', 'ZMAT2', 'ZNF165', 'ZNF222', 'ZNF223', 'ZNF256', 'ZNF385C', 'ZNF404', 'ZNF428', 'ZNF556', 'ZNF587', 'ZNF596', 'ZNF695', 'ZNF697', 'ZSCAN22']	26
11	['AKAP8', 'ALS2CR7', 'ARNT', 'BAZ2B', 'BCR-ABL_E282D', 'BCR-ABL_H396R', 'BHLHE40', 'BRAF_D594V', 'BRAF_G596R', 'BTBD12', 'C1ORF25', 'CCDC79', 'CD36', 'CDC2L1', 'CREB5', 'DDR2', 'DEPDC6', 'DPF3', 'DSTYK', 'DZIP3', 'EBF3', 'EGFR_G719S', 'EGFR_T790M', 'EGFR_insNPG', 'EGFR_vIII', 'EHMT2', 'ERBB4_E836K', 'ERCC6', 'FARSA', 'FASTKD1', 'FBXO18', 'FBXO34', 'FLT3_ITD', 'GTF2F1', 'H2AFX', 'H2AFY2', 'HECW2', 'HIPK2', 'HOGA1', 'HOXD10', 'IBTK', 'ICK', 'IKZF5', 'JAK3', 'JMJD2B', 'JMJD2D', 'JMJD5', 'KBTBD8', 'KLF12', 'KLF17', 'KLHL28', 'LARP6', 'LMTK2', 'LMX1B', 'MAP3K15', 'MAPK4', 'MEF2D', 'MET', 'MGRN1', 'MLLT3', 'MTOR', 'MTOR_KIN', 'MXD1', 'MXI1', 'MYST2', 'NAP1L1', 'NEDD8', 'NEK8', 'NFIA', 'NOC4L', 'NR1I2', 'PAX6', 'PLEKHA4', 'PLK4', 'PNCK', 'PRKR', 'RBM22', 'RBM26', 'RCBTB2', 'REST', 'RNF111', 'RNF17', 'RNF19B', 'RNF219', 'RNF40', 'RPA4', 'RSPRY1', 'SETBP1', 'SLC22A4', 'SLC30A9', 'SLK', 'SORBS2', 'SP100', 'TAX1BP1', 'TBX2', 'TESK2', 'TIE1', 'TIGD6', 'TNK1', 'TOX4', 'TRAF6', 'TRIM41', 'TRIM60', 'TRIM7', 'TRIM73', 'TRMT1', 'TUT1', 'UBR1', 'ULK2', 'VRK2', 'WWP2', 'YY1', 'ZBTB22', 'ZBTB9', 'ZEB2', 'ZNF10', 'ZNF155', 'ZNF20', 'ZNF23', 'ZNF232', 'ZNF26', 'ZNF276', 'ZNF277', 'ZNF30', 'ZNF430', 'ZNF446', 'ZNF460', 'ZNF510', 'ZNF513', 'ZNF529', 'ZNF567', 'ZNF571', 'ZNF599', 'ZNF619', 'ZNF69', 'ZNF711', 'ZNF768', 'ZNF772', 'ZNF79', 'ZNF821', 'ZNF827', 'ZNF83', 'ZSCAN2', 'ZUFSP', 'ZZZ3']	145
12	['ABCF3', 'ABCG8', 'ABL1', 'ABL1_G2A', 'ACTN4', 'ADCK4', 'AFF4', 'AFP', 'AHR', 'AKR1C2', 'AKT2', 'AKT3', 'ANXA2', 'AP2M1', 'APOA1', 'APOM', 'ARL4C', 'ARNTL', 'ARPC1A', 'ARPC4', 'ATF6B', 'ATG4A', 'ATP6V1D', 'AURKA', 'AZGP1', 'BAG6', 'BCR-ABL_E450K', 'BCR-ABL_F311L', 'BCR-ABL_F382A', 'BCR-ABL_M472I', 'BCR-ABL_Q346H', 'BCR-ABL_V289F', 'BIRC2', 'BLMH', 'BRAF_G466E', 'BRAF_G466V', 'BRAF_G469A', 'BRAF_G469E', 'BZW2', 'C1S', 'CAMK1', 'CAMLG', 'CASK', 'CASP3', 'CAT', 'CBLL1', 'CBX7', 'CCDC71', 'CCRL2', 'CCRN4L', 'CD81', 'CD83', 'CD97', 'CDC2L5', 'CDK10', 'CDK5', 'CDK8', 'CDKL4', 'CEP72', 'CERS4', 'CHEK1', 'CHRAC1', 'CLIC4', 'CLPTM1', 'COASY', 'COPS5', 'COPZ1', 'COX4I1', 'CPD', 'CRYZ', 'CSNK1G3', 'CTTN', 'CXCR7', 'CYP1A1',	403

	'DAPK2', 'DARS', 'DBI', 'DEK', 'DEPDC7', 'DET1', 'DHCR24', 'DHX8', 'DIDO1', 'DLGAP5', 'DLX2', 'DMRTA1', 'DNAJB6', 'DNM1L', 'DPF2', 'DSE', 'DTNA', 'DTX3L', 'DTX4', 'DUT', 'DYNLT3', 'EBF4', 'EIF2AK1', 'EIF4A2', 'ENST00000356364', 'EPHA3', 'EPHX2', 'EPS8', 'ERBB4', 'ERBB4_E872K', 'EYA1', 'F2RL1', 'F3', 'FAM114A2', 'FBXO10', 'FBXO11', 'FBXO3', 'FBXO42', 'FBXO46', 'FERD3L', 'FEZ1', 'FGB', 'FGFR3', 'FIGF', 'FKBP3', 'FOXC2', 'FOXN3', 'FOXR1', 'FTL', 'FZD7', 'GAA', 'GABARAPL1', 'GFOD1', 'GJA1', 'GLI3', 'GNA11', 'GNA13', 'GNA15', 'GNAI1', 'GNAZ', 'GNB2', 'GOT1', 'GPR128', 'GPR83', 'GRHL3', 'GRPR', 'GSG2', 'GSTT1', 'GTF2A2', 'HADHA', 'HADHB', 'HAL', 'HBP1', 'HDAC11', 'HESX1', 'HIST1H1A', 'HIST1H1T', 'HSF5', 'ICAM1', 'ICMT', 'IGF2', 'IL10', 'INPP1', 'IRF3', 'IRF9', 'IRS1', 'KCNA6', 'KCNK1', 'KCNQ1', 'KCTD8', 'KLF10', 'KLHL1', 'KLHL12', 'KLHL36', 'LAMTOR3', 'LARS2', 'LBR', 'LCOR', 'LEF1', 'LEPRE1', 'LGALS8', 'LIAS', 'LONRF3', 'LSM6', 'LYK5', 'LYPLA1', 'MAEL', 'MAGEA10', 'MAOA', 'MAP3K12', 'MAP3K2', 'MAP3K5', 'MAP4K3', 'MAPK15', 'MAPK3', 'MAS1L', 'MAST2', 'MCM7', 'METTL14', 'MET_Y1248C', 'MET_Y1252C', 'MEX3D', 'MGC16169', 'MGLL', 'MINA', 'MKNK1', 'MKRN2', 'MLH1', 'MPZL1', 'MRGPRX3', 'MTFR1', 'NAA50', 'NAGK', 'NAT1', 'NCOA1', 'NCOA3', 'NEDD4L', 'NFAT5', 'NMI', 'NQO2', 'NR0B1', 'NRIP3', 'NRP1', 'NUDT14', 'NUDT6', 'NUP62', 'OGG1', 'OVOL1', 'PA2G4', 'PAK7', 'PAQR7', 'PARP15', 'PAX7', 'PBK', 'PCK2', 'PCTK3', 'PDGFRB', 'PDHA1', 'PDK4', 'PEMT', 'PFN2', 'PFTK2', 'PHF19', 'PIAS1', 'PIGK', 'PIK3CB', 'PIK3CD', 'PIK3CG', 'PIK3R1', 'PIN1', 'PITPNA', 'PLA2G12A', 'PLOD3', 'PNP', 'POLA2', 'POLE', 'POLE4', 'PPIE', 'PPOX', 'PPP2R3C', 'PPP3CC', 'PPT1', 'PRDM14', 'PRKAB1', 'PRKACB', 'PRKCA', 'PRKCB', 'PRKG2', 'PRKRIR', 'PRNP', 'PRPF19', 'PRPF6', 'PRR15L', 'PRSS2', 'PSKH1', 'PSMA3', 'PSMB10', 'PTK2', 'PTP4A3', 'PVRL2', 'RAB11A', 'RAB11FIP2', 'RAB1B', 'RAB5A', 'RALA', 'RASD1', 'RASSF1', 'RBBP7', 'RBM15', 'RCOR3', 'RING1', 'RIOK2', 'RND3', 'RNF138', 'RNF31', 'ROS', 'RPL22', 'RPL39L', 'RPS15A', 'RPS3', 'RPS6KA3', 'RPS6KB1', 'RRP8', 'RUFY1', 'RUVBL2', 'S100A1', 'S100A13', 'SCAF8', 'SCYL3', 'SHMT2', 'SIRT4', 'SIRT5', 'SLC22A18', 'SLC25A13', 'SLC25A3', 'SLC30A8', 'SLFN11', 'SMAD7', 'SMARCC2', 'SMU1', 'SOAT1', 'SOCS6', 'SOS2', 'SOX6', 'SP2', 'SPECC1L', 'SQLE', 'SRPK1', 'SRPK3', 'SSX3', 'ST18', 'STK17A', 'STK24', 'STRADB', 'TADA3', 'TAF15', 'TBC1D9B', 'TBK1', 'TCF7L2', 'TDRD3', 'TERF2IP', 'TESK1', 'TFDP1', 'THAP5', 'TIGD4', 'TIGD7', 'TIMM50', 'TMED9', 'TMEM110', 'TMEM174', 'TNFRSF6B', 'TNFSF10', 'TNFSF13', 'TNK2', 'TOX', 'TOX2', 'TPI1', 'TRAF1', 'TRIM17', 'TRIM23', 'TRIM26', 'TRIM37', 'TRIM39', 'TSC22D4', 'TSPAN6', 'TSPAN8', 'TSSK6', 'TUBB4B', 'UBE2E3', 'USP1', 'USP15', 'UTP14A', 'VAPB', 'WNT7B', 'YES1', 'YTHDF2', 'YWHAQ', 'ZBTB45', 'ZC3HC1', 'ZIM3', 'ZKSCAN1', 'ZKSCAN2', 'ZNF175', 'ZNF192', 'ZNF195', 'ZNF217', 'ZNF266', 'ZNF274', 'ZNF280A', 'ZNF343', 'ZNF350', 'ZNF415', 'ZNF449', 'ZNF462', 'ZNF550', 'ZNF551', 'ZNF561', 'ZNF564', 'ZNF570', 'ZNF573', 'ZNF577', 'ZNF585A', 'ZNF622', 'ZNF669', 'ZNF670', 'ZNF77', 'ZNF85', 'ZNF98', 'ZSCAN29']	
13	['ACVR1B', 'ARIH2', 'ASB3', 'ATF7', 'BCR-ABL_E494A', 'BCR-ABL_K271N', 'BCR-ABL_Y440C', 'BMPR2', 'BRAF_G466A', 'BTBD1', 'C9ORF11', 'CDC2L6', 'CHD1', 'CHST12', 'CREM', 'CSDC2', 'CUL1', 'CUL4B', 'CUL5', 'DEPDC5', 'DMPK', 'ENST00000265296', 'ERBB2', 'ERN1', 'FBXO40', 'FEZF2', 'FLJ23356', 'FOXN2', 'GRK7', 'HAND1', 'HERC4', 'HLX', 'HOXA3', 'ID1', 'IRF6', 'KIAA0317', 'KIAA0415', 'KLHL18', 'LIN28', 'LMX1A', 'MAP2K7', 'MAP3K9', 'MAPK7', 'MARK1', 'MBD4', 'MDM2', 'MYNN', 'NEK10', 'NEUROD1', 'NEUROG1', 'NR1D2', 'NR2E3', 'NUAK2', 'PAK2', 'PBRM1', 'PDS5B', 'PKN1', 'PKN2', 'POU2F2', 'PPIL2', 'PRDM10', 'PRKD2', 'PRKX', 'PSKH2', 'RAD18', 'RAG1', 'RBCK1', 'RBM5', 'RCC1', 'RLF', 'RNF213', 'RNF8', 'RORA', 'SETD5', 'SETDB2', 'SHPRH', 'SMARCC1', 'SNAI1', 'TAOK3', 'TBX15', 'TGIF2', 'THRA', 'TNNI3K', 'TP63', 'TRIM22', 'TRIM36', 'TRIM46', 'TRIM56', 'TRIT1', 'TSSK1B', 'UBE4A', 'VPS41', 'VPS72', 'WNK4', 'WWP1', 'XIAP', 'ZBED4', 'ZBTB47', 'ZCCHC11', 'ZCCHC6', 'ZEB1', 'ZFYVE26', 'ZIK1', 'ZKSCAN3', 'ZNF140', 'ZNF141', 'ZNF18', 'ZNF207', 'ZNF224', 'ZNF320', 'ZNF321', 'ZNF333', 'ZNF335', 'ZNF34', 'ZNF385D', 'ZNF394', 'ZNF410', 'ZNF416', 'ZNF417', 'ZNF440', 'ZNF468', 'ZNF497', 'ZNF509', 'ZNF521', 'ZNF536', 'ZNF544', 'ZNF549', 'ZNF592', 'ZNF679', 'ZNF71', 'ZNF718', 'ZNF75D', 'ZSCAN1']	133
14	['ITK', 'JMJD4', 'RIOK3']	3
15	['A2M', 'ABAT', 'ABCC3', 'ABHD2', 'ACVR1', 'ACY1', 'ADCY3', 'ADI1', 'ADK', 'ADRB2', 'ADSL', 'AES', 'AGL', 'AIRE', 'AK1', 'AKR1B1', 'AKT1', 'ALDH1A1', 'ALDH2', 'ALDH3A2', 'ALDOC', 'ANAPC5', 'ANPEP', 'ANXA1', 'ANXA7', 'APEH', 'APOA2', 'ARF6', 'ASAH1', 'ASNA1', 'ASS1', 'ATG5', 'ATIC', 'ATP1A3', 'ATP5A1', 'ATP5D', 'AURKAIP1', 'B4GALT3', 'BCKDK', 'BCL6', 'BEND5', 'BID', 'BLK', 'BLVRA', 'BLZF1', 'BMPR1A', 'BNIP3L', 'BRPF1', 'C19ORF10', 'CALM2', 'CALR', 'CAMK2D', 'CARD9', 'CBR1', 'CCBL1', 'CCL2', 'CCNC', 'CCNE1', 'CD59', 'CDC7', 'CDK16', 'CDK4', 'CDK6', 'CDX1', 'CERS2', 'CHAF1B', 'CHGA', 'CHMP6', 'CISH', 'CKS1B', 'CLCN5', 'CNOT7', 'COBL', 'COG2', 'COQ3', 'CPLX2', 'CPSF4', 'CPT1A', 'CRABP1', 'CREB3L1', 'CSF3', 'CSNK1A1', 'CSNK1A1L', 'CSNK1D', 'CSNK1E', 'CSNK2B', 'CTH', 'CTLA4', 'CXCR4', 'CYB561', 'CYCS', 'CYP51A1', 'DAP', 'DAXX', 'DCP1A', 'DCPS', 'DDIT3', 'DDR1', 'DERL1', 'DFFB', 'DHFR', 'DHH', 'DHRS7', 'DKC1', 'DNAJB2', 'DNAJB9', 'DNMT3A', 'DOK4', 'DRD1', 'DUSP3', 'DVL2', 'DYRK3', 'E2F6', 'EBNA1BP2', 'ECD', 'ECSIT', 'EFCAB2', 'EGLN3', 'EGR3', 'EIF2AK2', 'EIF2B2', 'EIF2C1', 'EIF4E3', 'EIF5A', 'EMD', 'ENOPH1', 'ENOSF1', 'EPHX1', 'EPO', 'ERCC3', 'ERGIC1', 'ERGIC2', 'ERH', 'ETS2', 'ETV1', 'ETV5', 'EVL', 'EXOSC4', 'F10', 'FABP6', 'FANCG', 'FAS', 'FCHO1', 'FDXR', 'FGFR1', 'FGG', 'FHL2', 'FIBP', 'FOS', 'FOXA3', 'FOXJ1', 'FOXO3', 'FURIN', 'FUT1', 'FZD4', 'GABBR1', 'GALE', 'GAMT', 'GAPDH', 'GAS7', 'GBA', 'GCLC', 'GCM2', 'GDI1', 'GGA2', 'GGT1', 'GLOD4', 'GNAI3', 'GNB1', 'GNG5', 'GNG8', 'GOT2', 'GPR107', 'GPR132', 'GPR139', 'GPR141', 'GPR152', 'GPR34', 'GPR35', 'GRAP', 'GRN', 'GSTP1', 'GTF2F2', 'GUK1', 'GUSB', 'HAND2', 'HAT1', 'HBE1', 'HCAR1', 'HIF1AN', 'HIST1H2BD', 'HLA-DPB1', 'HMGB1', 'HMGB3', 'HMGN4', 'HN1L', 'HNMT', 'HOOK2', 'HRAS', 'HSD17B2', 'HYAL1', 'ID3', 'IDH3B', 'IGF2BP3', 'IGFBP7', 'IKBIP', 'IKBKE', 'IKBKG', 'IL12A', 'IL13RA1', 'IL4', 'IL8', 'ILF3', 'IMPDH2', 'ING2', 'IQSEC1', 'JAK2', 'KARS', 'KAT5', 'KCNJ11', 'KDELR2', 'KIAA1279', 'KISS1R', 'KLHDC3', 'KLHL2', 'KSR2', 'LAP3', 'LARS', 'LDHA', 'LGR5', 'LHX8', 'LHX9', 'LIMK2', 'LMNA', 'LPAR1', 'LPAR5', 'LRPAP1', 'MAGED1', 'MAP3K8', 'MAPK9', 'MBNL2', 'MCCC1', 'MCF2L', 'MCL1', 'MCOLN1', 'MED7', 'MEIS2', 'MERTK', 'METTL1', 'MIF', 'MITF', 'MKI67IP', 'MLYCD', 'MMP1', 'MOK', 'MRGPRF', 'MSH2', 'MSRB2', 'MTF2', 'MUSK', 'MYB', 'MYCBP', 'MYL6', 'NCOA4', 'NDRG1', 'NDUFA4', 'NDUFA9', 'NDUFB1', 'NDUFC2', 'NFATC3', 'NGEF', 'NKIRAS2', 'NMT1', 'NONO', 'NR0B2', 'NR1H3', 'NR2F6', 'NR5A2', 'NRBP2', 'NTAN1', 'NUSAP1', 'NXF1', 'OAT', 'ORMDL3', 'OXER1', 'OXTR', 'PAICS', 'PANK4', 'PC', 'PCBD1', 'PDIA4', 'PDIA5', 'PDK2', 'PDXK', 'PECR', 'PFAS', 'PFKFB3', 'PFKL', 'PGD', 'PHKG2',	485

	'PI16', 'PIK3R4', 'PIPOX', 'PJA1', 'PJA2', 'PKM2', 'PLCB1', 'PLK1', 'PLK2', 'POLE2', 'POLR2C', 'POLR2E', 'POLR3C', 'PPAP2A', 'PPAT', 'PPP1R13B', 'PPP1R8', 'PPP2R1A', 'PPP3R2', 'PQBP1', 'PRKAG1', 'PRKAR2B', 'PRKCE', 'PRKCH', 'PRKCQ', 'PRKCZ', 'PRPF4', 'PSMB8', 'PSMD10', 'PSMD2', 'PSMD5', 'PSPH', 'PTGFR', 'PTGS2', 'PUF60', 'PUS7L', 'PVR', 'PVRL1', 'PYGL', 'RAB31', 'RAB4A', 'RAC1', 'RAE1', 'RARB', 'RASGRP2', 'RASGRP4', 'RBBP6', 'RBMS1', 'RBP4', 'RCCD1', 'RDH11', 'RERG', 'RFK', 'RFWD2', 'RFX6', 'RFXANK', 'RHEB', 'RHOC', 'RHOD', 'RNF145', 'RNF146', 'RNF166', 'RNF167', 'RNF25', 'RNF5', 'RNH1', 'RNMT', 'RPL21', 'RPS13', 'RPS6KA5', 'RPS6KB2', 'RPS6KL1', 'RRAGA', 'RRAGC', 'RRM2', 'RUVBL1', 'RXFP4', 'S100P', 'SCCPDH', 'SERPINB2', 'SETD7', 'SFPQ', 'SIRT3', 'SLC1A5', 'SLC22A23', 'SLC25A15', 'SLC25A4', 'SLC25A5', 'SLC35A4', 'SMAD1', 'SMARCE1', 'SNCA', 'SNW1', 'SOCS2', 'SP6', 'SPRY2', 'SREBF1', 'SRPRB', 'SRSF4', 'SSBP4', 'ST14', 'STK33', 'STK38L', 'STXBP1', 'SUCNR1', 'SUOX', 'SUPT5H', 'SUV420H1', 'SYK', 'SYPL1', 'TADA2A', 'TAL2', 'TBX20', 'TCEA1', 'TCTA', 'TCTN1', 'TEAD2', 'TEAD4', 'TEX11', 'TFEB', 'TGIF2LY', 'TIMM17B', 'TIMP2', 'TIMP3', 'TLR8', 'TM7SF2', 'TMEM97', 'TNFRSF10A', 'TNFSF13B', 'TP53RK', 'TPMT', 'TRIB3', 'TSPAN4', 'TSSC4', 'TSTA3', 'TUBB2A', 'TWIST2', 'TXNDC9', 'TXNIP', 'UBAP1', 'UBAP2L', 'UBE2K', 'UBE3B', 'UBE3C', 'UCK2', 'UGT1A9', 'ULK3', 'UNC45B', 'UROD', 'USF1', 'UXT', 'VARS', 'VIPR1', 'VN1R2', 'VN1R4', 'WNT10B', 'WT1', 'WWTR1', 'XPC', 'YAP1', 'YARS', 'YEATS4', 'YKT6', 'YTHDF1', 'ZBTB49', 'ZNF133', 'ZNF300', 'ZNF418', 'ZNF436', 'ZNF473', 'ZNF490', 'ZNF503', 'ZNF519', 'ZNF543', 'ZNF562', 'ZNF563', 'ZNF569', 'ZNF57', 'ZNF572', 'ZNF626', 'ZNF689', 'ZNF74', 'ZNF747', 'ZNF764', 'ZNF790']	
16	['ABL1_T315I', 'ALK', 'ALK_F1174L', 'ALK_R1275Q', 'ALK_T1151M', 'BMX', 'BRAF_A727V', 'BRAF_E586K', 'BRAF_G464V', 'BRAF_K601E', 'BRAF_V600E', 'CPSF1', 'EGFR_L858R', 'EGFR_L858R_T790M', 'EGFR_del3', 'FLT3_D835Y', 'IKZF1', 'LZTR1', 'MAP4K1', 'NR1H2', 'RET_M918T', 'RPS6KC1', 'ZNF639', 'ZNF655']	24
17	['ABCB5', 'ABCF2', 'ACAA1', 'ACADSB', 'ACAT2', 'ACD', 'ADA', 'ADAP2', 'ADCY9', 'ADH5', 'ADORA2A', 'AFG3L2', 'AGTR1', 'AHCY', 'AK2', 'AKR1C1', 'AKR1C3', 'ALDH3A1', 'ALDH3B1', 'ALDH3B2', 'ALDH6A1', 'ALG2', 'AMBP', 'AMPD2', 'ANKZF1', 'AOC3', 'AP1S2', 'ARAF', 'ARF1', 'ARG1', 'ARHGEF19', 'ARHGEF2', 'ARHGEF3', 'ARHGEF7', 'ARHGEF9', 'ARIH1', 'ARPC1B', 'ARPC3', 'ARRB1', 'ASH2L', 'ASPHD1', 'ATF2', 'ATF4', 'ATG10', 'ATMIN', 'ATP5B', 'ATP5F1', 'ATP5H', 'ATP5S', 'ATP6V0C', 'AURKB', 'AXIN2', 'B2M', 'B3GAT3', 'BAG1', 'BATF3', 'BAX', 'BCL2L1', 'BCL6B', 'BHMT2', 'BIK', 'BIRC5', 'BLCAP', 'BMI1', 'BMP4', 'BRAF', 'BRCA1', 'BRD3', 'BRD4', 'BRF2', 'BRMS1L', 'BRP44', 'BTG1', 'BTG3', 'BTK', 'BTRC', 'BUB1B', 'BUD13', 'C16ORF80', 'C5ORF62', 'CAB39', 'CALM3', 'CALU', 'CAPG', 'CAPNS2', 'CARS', 'CASP6', 'CASP7', 'CASP9', 'CAV1', 'CBFB', 'CBLB', 'CBLC', 'CBR3', 'CBS', 'CCDC150', 'CCND1', 'CCND2', 'CCNG1', 'CCNH', 'CCP110', 'CCT7', 'CD19', 'CD24', 'CD320', 'CD55', 'CDC20', 'CDC25A', 'CDC25B', 'CDC25C', 'CDCA4', 'CDCA8', 'CDIPT', 'CDK1', 'CDK2', 'CDK7', 'CDK9', 'CDKAL1', 'CDO1', 'CEP55', 'CERS3', 'CETN3', 'CFB', 'CFLAR', 'CGRRF1', 'CHEK2', 'CHN1', 'CHRM3', 'CKB', 'CLK2', 'CLOCK', 'CMPK1', 'CNOT8', 'CNPY3', 'COPS2', 'COPS4', 'CORO1A', 'COX5A', 'COX6B2', 'CPE', 'CRCP', 'CREB1', 'CREB3', 'CREB3L4', 'CRK', 'CRKL', 'CS', 'CSNK1G2', 'CSNK2A1', 'CTBP1', 'CTBP2', 'CTCF', 'CTCFL', 'CTGF', 'CTNNBIP1', 'CTSK', 'CTSL2', 'CUL2', 'CXADR', 'CXCR2', 'CYP20A1', 'CYP2E1', 'CYP3A5', 'DAPK1', 'DCTN6', 'DDB2', 'DDIT4', 'DDOST', 'DDX49', 'DECR1', 'DFFA', 'DGCR14', 'DGKA', 'DGKZ', 'DHDDS', 'DHRS2', 'DICER1', 'DLD', 'DMRTC2', 'DMTF1', 'DNAJC15', 'DNAJC5B', 'DNTTIP2', 'DR1', 'DTX2', 'DUSP4', 'DYRK2', 'EBP', 'EEF1D', 'EFNB3', 'EGFL8', 'EGFR', 'EHF', 'EIF2S2', 'EIF3G', 'EIF4B', 'EIF4H', 'ELF1', 'ELF2', 'ELF4', 'ELL3', 'ERBB3', 'ERCC1', 'ERCC5', 'ERLIN1', 'ERO1L', 'ESD', 'ESR2', 'ETFB', 'ETNK1', 'EWSR1', 'EXOC3', 'EXOSC8', 'EZH1', 'EZH2', 'F11', 'FADS3', 'FAM120A', 'FAM20B', 'FASLG', 'FASTKD5', 'FBXL20', 'FBXO7', 'FBXW11', 'FCGR2A', 'FDPS', 'FFAR1', 'FGF9', 'FGFR2', 'FGFRL1', 'FGR', 'FKBP14', 'FKBP4', 'FLT1', 'FLT3', 'FOSL1', 'FOXA1', 'FOXJ2', 'FOXO4', 'FOXP3', 'FOXP4', 'FRS3', 'FUS', 'FXR2', 'FXYD2', 'G3BP1', 'G3BP2', 'G6PD', 'GAB1', 'GABPB2', 'GALNS', 'GAS2L1', 'GATAD2B', 'GBGT1', 'GBP2', 'GCA', 'GCDH', 'GFPT2', 'GK', 'GLUL', 'GM2A', 'GMNN', 'GMPR2', 'GNAI2', 'GNB5', 'GNGT2', 'GNPDA1', 'GPBAR1', 'GPER', 'GPR101', 'GPR114', 'GPR119', 'GPR148', 'GPR156', 'GPR160', 'GPR161', 'GPR3', 'GPR37', 'GPR39', 'GPR4', 'GPR52', 'GPR55', 'GPR84', 'GPR87', 'GPX7', 'GRB2', 'GRB7', 'GSTZ1', 'GTF2E2', 'GTPBP8', 'HAGH', 'HAX1', 'HCK', 'HDAC4', 'HINT1', 'HIST1H2AC', 'HIST1H2BK', 'HIST2H2BE', 'HK1', 'HLA-DMB', 'HLA-DRB1', 'HLA-DRB5', 'HMG20B', 'HMGA1', 'HMGB2', 'HMGB4', 'HMGCL', 'HMGCS2', 'HMOX2', 'HOMEZ', 'HOPX', 'HORMAD2', 'HOXA10', 'HOXA2', 'HOXA5', 'HOXB5', 'HOXB7', 'HOXC10', 'HOXC4', 'HPGD', 'HPRT1', 'HRH1', 'HS2ST1', 'HSD17B10', 'HSD17B11', 'HSD17B4', 'HSF1', 'HSF1_H2F2', 'HSF2', 'HSP90AA1', 'HSPA2', 'HSPA9', 'HSPD1', 'HTATIP2', 'HTATSF1', 'ICAM3', 'IDH3A', 'IDH3G', 'IFI16', 'IFI30', 'IFIT5', 'IGF1R', 'IGFBP6', 'IGHM', 'IGSF8', 'IKBKB', 'IL15', 'IL18RAP', 'IL1B', 'IL1R2', 'IL1RAP', 'IL20', 'IL21R', 'IL7R', 'ILF2', 'ILK', 'ING3', 'INS', 'INSIG1', 'IRAK4', 'IREB2', 'IRF2', 'IRF4', 'IRF8', 'ISG20', 'ITGAE', 'JMJD6', 'KCTD1', 'KDELR3', 'KIF14', 'KIF2C', 'KIF9', 'KITLG', 'KLF4', 'KLHL10', 'KLHL34', 'KLHL9', 'KYNU', 'LACC1', 'LAGE3', 'LARP4', 'LCK', 'LDHB', 'LGALS1', 'LGALS2', 'LGALS3', 'LHPP', 'LHX4', 'LIG3', 'LINC00479', 'LIPH', 'LNX1', 'LPAR4', 'LPAR6', 'LPXN', 'LRRC4', 'LRSAM1', 'LSM2', 'LSM5', 'LSR', 'LYN', 'MAD2L1BP', 'MAGEA9', 'MAOB', 'MAP3K14', 'MAP7', 'MAPK12', 'MAPK1IP1L', 'MAPKAPK2', 'MAS1', 'MCCC2', 'MCM2', 'MCM3', 'MCM5', 'MCM8', 'MDH2', 'MECP2', 'MED21', 'MED28', 'MED4', 'MEF2A', 'MFSD10', 'MGST2', 'MKNK2', 'MKX', 'MLLT10', 'MLLT11', 'MMP14', 'MMP2', 'MMP7', 'MORF4L1', 'MPHOSPH9', 'MPPED1', 'MRPL12', 'MRPL43', 'MSH5', 'MTERFD1', 'MTX2', 'MVP', 'MXD3', 'MYLK2', 'MYOG', 'NAB2', 'NAE1', 'NAMPT', 'NARFL', 'NASP', 'NAT10', 'NDUFA3', 'NDUFS3', 'NDUFV1', 'NDUFV2', 'NEK6', 'NET1', 'NEU1', 'NF2', 'NFE2', 'NFE2L1', 'NFE2L2', 'NFIB', 'NFIL3', 'NFKB1', 'NISCH', 'NIT1', 'NKX2-5', 'NLK', 'NMNAT2', 'NMRAL1', 'NMUR2', 'NOSIP', 'NOTCH2NL', 'NOX1', 'NOXA1', 'NPM1', 'NPRL2', 'NR2C1', 'NRBF2', 'NRBP1', 'NRG1', 'NRIP1', 'NSDHL', 'NTRK3', 'NUP88', 'OLIG3', 'OPA1', 'OPN4', 'OSR2', 'OTX1', 'OXCT1', 'OXGR1', 'P2RY6', 'P2RY8', 'P4HA2', 'P4HB', 'PACSIN3', 'PAF1', 'PAFAH1B3', 'PAH', 'PAK1', 'PAK3', 'PAK4', 'PANK2', 'PAPSS1', 'PASD1', 'PAX3', 'PAX8', 'PBX2', 'PCCB', 'PCDHA10', 'PCDHGB1', 'PCGF2', 'PCGF3', 'PCNA', 'PDE4D', 'PDLIM1', 'PDS5A', 'PEPD', 'PEX11A', 'PGBD1', 'PHF11', 'PHF13', 'PHF17', 'PHF23', 'PHF5A', 'PHYH', 'PI4KAP2', 'PIGA', 'PIGB', 'PIK3R3', 'PIM1', 'PITX1', 'PITX2', 'PLA2G12B', 'PLAU', 'PLAUR', 'PLD1', 'PLEKHG3', 'PLEKHG6', 'PLEKHJ1', 'PLOD1', 'PML', 'PNKP', 'PNLDC1', 'PNN', 'POFUT1', 'POLG2', 'POLR2F', 'POLR2I', 'POLR3D', 'POP4', 'POT1', 'POU2AF1', 'PPAP2B', 'PPARG', 'PPFIBP2', 'PPIA', 'PPM1B', 'PPP1CA', 'PPP1CC', 'PPP2R3A', 'PPP2R4', 'PPP3CA', 'PPP3CB', 'PPP4C', 'PRAF2',	875

	'PRDM4', 'PREB', 'PRKAA1', 'PRKACA', 'PRKAG2', 'PRKAG3', 'PRKAR2A', 'PROC', 'PSEN1', 'PSEN2', 'PSMA1', 'PSMA8', 'PSMB1', 'PSMB2', 'PSMB7', 'PSMB9', 'PSMC5', 'PSMD3', 'PSMD4', 'PSMD9', 'PSME2', 'PSMF1', 'PSMG1', 'PTGER4', 'PTHLH', 'PTPLA', 'PTPRF', 'PTRF', 'PTS', 'PTTG1', 'PURG', 'PWP1', 'PXK', 'QPCT', 'RAD23B', 'RAF1', 'RALB', 'RAMP1', 'RAN', 'RAP1A', 'RAP1GDS1', 'RAPGEF4', 'RARRES3', 'RASGRP3', 'RASSF2', 'RBBP4', 'RBM15B', 'RBM6', 'RBMX', 'RBPJ', 'RBX1', 'RDX', 'RELB', 'RFC5', 'RFX4', 'RFX5', 'RG9MTD3', 'RGS11', 'RGS18', 'RHOBTB1', 'RHOBTB2', 'RHOXF1', 'RIT2', 'RNASET2', 'RNF14', 'RNF20', 'RNF7', 'RNGTT', 'RNPS1', 'RPL11', 'RPL35A', 'RPL7', 'RPN2', 'RPS14', 'RPS16', 'RPS19', 'RPS27A', 'RPS6KA1', 'RPS6KA4', 'RPS7', 'RRM1', 'RRS1', 'RSPH1', 'RXRG', 'RYBP', 'S100A6', 'SALL4', 'SAMD4A', 'SAMD4B', 'SARS', 'SATB1', 'SC5DL', 'SDHA', 'SDHB', 'SDHD', 'SENP6', 'SERINC3', 'SERPINA6', 'SERPINA7', 'SERPINB6', 'SERPIND1', 'SETDB1', 'SF1', 'SGK3', 'SH3BP5', 'SH3RF2', 'SHC1', 'SIK1', 'SIM2', 'SIRPG', 'SIRT2', 'SIX2', 'SKP2', 'SLC1A1', 'SLC22A5', 'SLC25A14', 'SLC25A22', 'SLC25A32', 'SLC25A6', 'SLC27A3', 'SLC29A1', 'SLC2A1', 'SLC35A1', 'SLC35F2', 'SLC39A8', 'SLC52A2', 'SLC5A6', 'SLC7A1', 'SLC7A11', 'SLC7A9', 'SMAD4', 'SMAD5', 'SMARCAD1', 'SMARCB1', 'SMOX', 'SMURF2', 'SMYD3', 'SNAI2', 'SNAPC1', 'SNRPA', 'SNX11', 'SNX6', 'SNX7', 'SOCS4', 'SOCS5', 'SOD1', 'SORBS3', 'SORD', 'SPA17', 'SPATA13', 'SPDEF', 'SPIC', 'SPRED2', 'SPRY4', 'SQRDL', 'SQSTM1', 'SRC', 'SSBP2', 'SSX2', 'STAMBP', 'STAP2', 'STAT1', 'STAT2', 'STAT5A', 'STAT5B', 'STAT6', 'STK11', 'STK16', 'STK19', 'STK3', 'STMN1', 'STX4', 'SUB1', 'SULT1A1', 'SUPT4H1', 'SUPV3L1', 'SWAP70', 'SYF2', 'TACC3', 'TAF1B', 'TAF5L', 'TAGAP', 'TANK', 'TAP2', 'TCF12', 'TES', 'TFCP2', 'TFDP2', 'TFPI', 'TGDS', 'TGFA', 'TGFB1', 'TGFBR1', 'TGFBR2', 'TGIF1', 'TGM2', 'THRAP3', 'TICAM2', 'TIMM9', 'TIMP4', 'TK1', 'TLE2', 'TLK2', 'TLR5', 'TMED7', 'TMEM154', 'TMEM5', 'TNFAIP1', 'TNFAIP3', 'TNFRSF13B', 'TNIP1', 'TOMM22', 'TOR1A', 'TP53', 'TPD52L2', 'TPD52L3', 'TRAF3IP2', 'TRAFD1', 'TRAK2', 'TRAM2', 'TRIB1', 'TRIM16', 'TRIM2', 'TRIM32', 'TRIM50', 'TRIM9', 'TRIP13', 'TSEN2', 'TUBA1A', 'TULP2', 'TXNL1', 'TYRO3', 'U2AF1', 'UBA52', 'UBE2L6', 'UCHL1', 'UFD1L', 'UFM1', 'UGDH', 'UGP2', 'UQCRFS1', 'UTS2R', 'VAV1', 'VDR', 'VHL', 'VKORC1', 'VPS28', 'WARS', 'WDR18', 'WDR5', 'WDTC1', 'WNT1', 'WNT5A', 'WNT9A', 'XPR1', 'XRCC4', 'XRCC6', 'YAF2', 'ZAK', 'ZBTB20', 'ZBTB24', 'ZBTB25', 'ZBTB48', 'ZFP161', 'ZFP28', 'ZFP3', 'ZNF114', 'ZNF131', 'ZNF174', 'ZNF200', 'ZNF202', 'ZNF213', 'ZNF219', 'ZNF22', 'ZNF227', 'ZNF24', 'ZNF263', 'ZNF267', 'ZNF268', 'ZNF273', 'ZNF331', 'ZNF398', 'ZNF423', 'ZNF426', 'ZNF454', 'ZNF488', 'ZNF517', 'ZNF524', 'ZNF546', 'ZNF548', 'ZNF554', 'ZNF555', 'ZNF558', 'ZNF576', 'ZNF581', 'ZNF582', 'ZNF583', 'ZNF595', 'ZNF621', 'ZNF625', 'ZNF653', 'ZNF672', 'ZNF673', 'ZNF677', 'ZNF680', 'ZNF707', 'ZNF774', 'ZNF786', 'ZRSR2', 'ZSWIM2']	

Supplementary Table S5. The distribution of inhibitory targets repositioned from the original disease class to other disease classes. Each element represents the number of inhibitory targets repositioned. The rows indicate the original ICD disease chapters, and the number in parentheses represents the number of known inhibitory targets belonging to the disease chapters. The columns indicate the newly predicted ICD disease chapters. The chapters are as follows: Chapter I: certain infectious or parasitic dis-eases; Chapter II: neoplasms; Chapter III: diseases of the blood or blood-forming organs; Chapter IV: diseases of the immune system; Chapter V: endocrine, nutritional, or metabolic diseases; Chapter VI: mental, behavioral, or neurodevelopmental disorders; Chapter VII: sleep–wake disorders; Chapter VIII: diseases of the nervous system; Chapter IX: diseases of the visual system; Chapter X: diseases of the ear or mastoid process; Chapter XI: diseases of the circulatory system; Chapter XII: diseases of the respiratory system; Chapter XIII: diseases of the digestive system; Chapter XIV: diseases of the skin; Chapter XV: diseases of the musculoskeletal system or connective tissue; Chapter XVI: diseases of the genitourinary system; Chapter XVII: conditions related to sexual health; Chapter XVIII: pregnancy, childbirth, or the puerperium; Chapter XIX: certain conditions originating in the perinatal period; Chapter XX: developmental anomalies; Chapter XXI: symptoms, signs, or clinical findings, not else-where classified; and Chapter XXII: injury, poisoning, or certain other consequences of external causes. A network visualizing this matrix is shown in Fig. 5 (A).

ICD chapter (# of targets)	I	II	III	IV	V	VI	VII	VIII	IX	X	XI	XII	XIII	XIV	XV	XVI	XVII	XVIII	XIX	XX	XXI	XXII
chapter I (4)	0	0	0	0	0	0	0	0	0	0	0	0	0	0	0	0	0	0	0	0	0	0
chapter II (145)	0	34	0	8	0	0	0	15	0	0	0	0	0	7	4	0	0	0	0	0	0	0
chapter III (6)	0	0	0	0	0	0	0	0	0	0	0	0	0	0	0	0	0	0	0	0	0	0
chapter IV (20)	0	7	0	0	0	0	0	1	0	0	0	0	0	1	0	0	0	0	0	0	0	0
chapter V (31)	0	0	0	0	0	0	0	0	0	0	0	0	0	0	0	0	0	0	0	0	0	0
chapter VI (0)	0	0	0	0	0	0	0	0	0	0	0	0	0	0	0	0	0	0	0	0	0	0
chapter VII (0)	0	0	0	0	0	0	0	0	0	0	0	0	0	0	0	0	0	0	0	0	0	0
chapter VIII (13)	0	0	0	0	0	0	0	0	0	0	0	0	0	0	0	0	0	0	0	0	0	0
chapter IX (0)	0	0	0	0	0	0	0	0	0	0	0	0	0	0	0	0	0	0	0	0	0	0
chapter X (0)	0	0	0	0	0	0	0	0	0	0	0	0	0	0	0	0	0	0	0	0	0	0
chapter XI (0)	0	0	0	0	0	0	0	0	0	0	0	0	0	0	0	0	0	0	0	0	0	0
chapter XII (26)	0	0	0	0	0	0	0	0	0	0	0	0	0	0	0	0	0	0	0	0	0	0
chapter XIII (20)	0	0	0	0	0	0	0	0	0	0	0	0	0	0	0	0	0	0	0	0	0	0
chapter XIV (16)	0	7	0	1	0	0	0	1	0	0	0	0	0	0	1	0	0	0	0	0	0	0
chapter XV (47)	0	7	0	0	0	0	0	1	0	0	0	0	0	1	0	0	0	0	0	0	0	0
chapter XVI (0)	0	0	0	0	0	0	0	0	0	0	0	0	0	0	0	0	0	0	0	0	0	0
chapter XVII (0)	0	0	0	0	0	0	0	0	0	0	0	0	0	0	0	0	0	0	0	0	0	0
chapter XVIII (0)	0	0	0	0	0	0	0	0	0	0	0	0	0	0	0	0	0	0	0	0	0	0
chapter XIX (0)	0	0	0	0	0	0	0	0	0	0	0	0	0	0	0	0	0	0	0	0	0	0
chapter XX (0)	0	0	0	0	0	0	0	0	0	0	0	0	0	0	0	0	0	0	0	0	0	0
chapter XXI (0)	0	0	0	0	0	0	0	0	0	0	0	0	0	0	0	0	0	0	0	0	0	0
chapter XXII (0)	0	0	0	0	0	0	0	0	0	0	0	0	0	0	0	0	0	0	0	0	0	0

Supplementary Table S6. The distribution of activatory targets repositioned from the original disease class to other disease classes. Each element represents the number of activatory targets repositioned. The rows indicate the original ICD disease chapters, and the number in parentheses represents the number of known activatory targets belonging to the disease chapters. The columns indicate the newly predicted ICD disease chapters. The chapters are as follows: Chapter I: certain infectious or parasitic dis-eases; Chapter II: neoplasms; Chapter III: diseases of the blood or blood-forming organs; Chapter IV: diseases of the immune system; Chapter V: endocrine, nutritional, or metabolic diseases; Chapter VI: mental, behavioral, or neurodevelopmental disorders; Chapter VII: sleep–wake disorders; Chapter VIII: diseases of the nervous system; Chapter IX: diseases of the visual system; Chapter X: diseases of the ear or mastoid process; Chapter XI: diseases of the circulatory system; Chapter XII: diseases of the respiratory system; Chapter XIII: diseases of the digestive system; Chapter XIV: diseases of the skin; Chapter XV: diseases of the musculoskeletal system or connective tissue; Chapter XVI: diseases of the genitourinary system; Chapter XVII: conditions related to sexual health; Chapter XVIII: pregnancy, childbirth, or the puerperium; Chapter XIX: certain conditions originating in the perinatal period; Chapter XX: developmental anomalies; Chapter XXI: symptoms, signs, or clinical findings, not else-where classified; and Chapter XXII: injury, poisoning, or certain other consequences of external causes. A network visualizing this matrix is shown in Fig. 5 (B).

ICD chapter (# of targets)	I	II	III	IV	V	VI	VII	VIII	IX	X	XI	XII	XIII	XIV	XV	XVI	XVII	XVIII	XIX	XX	XXI	XXII
chapter I (0)	0	0	0	0	0	0	0	0	0	0	0	0	0	0	0	0	0	0	0	0	0	0
chapter II (9)	0	2	0	2	4	0	0	4	0	0	0	0	0	0	4	0	0	0	0	0	0	0
chapter III (0)	0	0	0	0	0	0	0	0	0	0	0	0	0	0	0	0	0	0	0	0	0	0
chapter IV (1)	0	2	0	0	1	0	0	1	0	0	0	0	0	0	1	0	0	0	0	0	0	0
chapter V (16)	0	8	0	1	4	0	0	5	0	0	0	0	0	0	4	0	0	0	0	0	0	0
chapter VI (0)	0	0	0	0	0	0	0	0	0	0	0	0	0	0	0	0	0	0	0	0	0	0
chapter VII (0)	0	0	0	0	0	0	0	0	0	0	0	0	0	0	0	0	0	0	0	0	0	0
chapter VIII (2)	0	2	0	1	2	0	0	0	0	0	0	0	0	0	1	0	0	0	0	0	0	0
chapter IX (0)	0	0	0	0	0	0	0	0	0	0	0	0	0	0	0	0	0	0	0	0	0	0
chapter X (0)	0	0	0	0	0	0	0	0	0	0	0	0	0	0	0	0	0	0	0	0	0	0
chapter XI (0)	0	0	0	0	0	0	0	0	0	0	0	0	0	0	0	0	0	0	0	0	0	0
chapter XII (0)	0	0	0	0	0	0	0	0	0	0	0	0	0	0	0	0	0	0	0	0	0	0
chapter XIII (1)	0	0	0	0	0	0	0	0	0	0	0	0	0	0	0	0	0	0	0	0	0	0
chapter XIV (0)	0	0	0	0	0	0	0	0	0	0	0	0	0	0	0	0	0	0	0	0	0	0
chapter XV (1)	0	2	0	0	0	0	0	1	0	0	0	0	0	0	0	0	0	0	0	0	0	0
chapter XVI (0)	0	0	0	0	0	0	0	0	0	0	0	0	0	0	0	0	0	0	0	0	0	0
chapter XVII (0)	0	0	0	0	0	0	0	0	0	0	0	0	0	0	0	0	0	0	0	0	0	0
chapter XVIII (0)	0	0	0	0	0	0	0	0	0	0	0	0	0	0	0	0	0	0	0	0	0	0
chapter XIX (0)	0	0	0	0	0	0	0	0	0	0	0	0	0	0	0	0	0	0	0	0	0	0
chapter XX (1)	0	0	0	0	0	0	0	0	0	0	0	0	0	0	0	0	0	0	0	0	0	0
chapter XXI (0)	0	0	0	0	0	0	0	0	0	0	0	0	0	0	0	0	0	0	0	0	0	0
chapter XXII (0)	0	0	0	0	0	0	0	0	0	0	0	0	0	0	0	0	0	0	0	0	0	0

Supplementary Table S7. Newly predicted 52 inhibitory targets repositioned from the original disease to other diseases. These inhibitory target–disease associations are predicted by only the trans-disease method with the completed data. Inhibitory targets are listed in decreasing order of predicted scores. Predicted scores represent the therapeutic targetability for the diseases.

Rank	Diseases	Inhibitory targets	Predicted scores
1	congenital muscular dystrophies (CMD/MDC)	MAPK11	1
2	congenital muscular dystrophies (CMD/MDC)	TNFSF13B	0.847
3	congenital muscular dystrophies (CMD/MDC)	TSLP	0.784
4	congenital muscular dystrophies (CMD/MDC)	CSF1R	0.739
5	congenital muscular dystrophies (CMD/MDC)	PARP1	0.639
6	congenital muscular dystrophies (CMD/MDC)	AURKC	0.454
7	cervical cancer	MAPK11	0.414
8	congenital muscular dystrophies (CMD/MDC)	MUC1	0.363
9	congenital muscular dystrophies (CMD/MDC)	TOP2B	0.325
10	systemic lupus erythematosus	MAPK11	0.32
11	ovarian cancer	TNFSF13B	0.271
12	cervical cancer	TNFSF13B	0.27
13	chronic myeloid leukemia (CML)	MAPK11	0.25
14	ovarian cancer	TSLP	0.242
15	congenital muscular dystrophies (CMD/MDC)	TUBB2A	0.235
16	cervical cancer	TSLP	0.228
17	ovarian cancer	CSF1R	0.215
18	systemic lupus erythematosus	TSLP	0.2
19	cervical cancer	CSF1R	0.192
20	systemic lupus erythematosus	CSF1R	0.175
21	chronic myeloid leukemia (CML)	TNFSF13B	0.164
22	atopic dermatitis	MAPK11	0.149
23	renal cell carcinoma	MAPK11	0.142
24	chronic myeloid leukemia (CML)	TSLP	0.134
25	systemic lupus erythematosus	PARP1	0.12
27	atopic dermatitis	TNFSF13B	0.091
28	gastric cancer	MAPK11	0.082
29	breast cancer	MAPK11	0.079
30	rheumatoid arthritis	MAPK11	0.076
31	renal cell carcinoma	TNFSF13B	0.071
32	chronic myeloid leukemia (CML)	PARP1	0.061
33	atopic dermatitis	CSF1R	0.056
34	renal cell carcinoma	TSLP	0.05
35	breast cancer	TNFSF13B	0.049
36	multiple myeloma	TNFSF13B	0.045
37	ovarian cancer	AURKC	0.044
38	breast cancer	TSLP	0.044
39	breast cancer	CSF1R	0.041
40	multiple myeloma	TSLP	0.035
41	rheumatoid arthritis	TSLP	0.031
42	colorectal cancer	MAPK11	0.029
43	gastric cancer	TNFSF13B	0.027
44	multiple myeloma	CSF1R	0.026
45	atopic dermatitis	PARP1	0.023
46	pancreatic cancer	MAPK11	0.022
47	rheumatoid arthritis	CSF1R	0.019
48	systemic lupus erythematosus	AURKC	0.018
49	gastric cancer	TSLP	0.013
50	multiple myeloma	PARP1	0.009
51	gastric cancer	CSF1R	0.001

26	chronic myeloid leukemia (CML)	CSF1R	0.113	52	breast cancer	AURKC	0

Supplementary Table S8. Newly predicted 46 activatory targets repositioned from the original disease to other diseases. These activatory target–disease associations are predicted by only the trans-disease method with the completed data. Activatory targets are listed in decreasing order of predicted scores. Predicted scores represent the therapeutic targetability for the diseases.

Rank	Diseases	Activatory targets	Predicted scores
1	acute myeloid leukemia (AML)	TH	1
2	rheumatoid arthritis	TH	0.981
3	type I diabetes mellitus	TH	0.981
4	chronic granulomatous disease	TH	0.929
5	acute myeloid leukemia (AML)	IFNG	0.871
6	rheumatoid arthritis	IFNG	0.855
7	type I diabetes mellitus	IFNG	0.855
8	acute myeloid leukemia (AML)	SOCS2	0.799
9	rheumatoid arthritis	SOCS2	0.789
10	type I diabetes mellitus	SOCS2	0.789
11	acute myeloid leukemia (AML)	PRKAB1	0.625
12	rheumatoid arthritis	PRKAB1	0.607
13	type I diabetes mellitus	PRKAB1	0.607
14	chronic granulomatous disease	SOCS2	0.591
15	breast cancer	TH	0.545
16	acute myeloid leukemia (AML)	SOCS4	0.503
17	rheumatoid arthritis	SOCS4	0.496
18	type I diabetes mellitus	SOCS4	0.496
19	acute myeloid leukemia (AML)	FFAR1	0.46
20	breast cancer	IFNG	0.45
21	rheumatoid arthritis	FFAR1	0.446
22	type I diabetes mellitus	FFAR1	0.446
23	acute myeloid leukemia (AML)	CTLA4	0.367

Rank	Diseases	Activatory targets	Predicted scores
24	Parkinson's disease (PD)	IFNG	0.35
25	type I diabetes mellitus	SYK	0.328
26	rheumatoid arthritis	SYK	0.328
27	Parkinson's disease (PD)	PRKAB1	0.32
28	Parkinson's disease (PD)	FFAR1	0.29
29	Parkinson's disease (PD)	SOCS2	0.287
30	breast cancer	PRKAB1	0.265
31	chronic granulomatous disease	PRKAB1	0.264
32	Parkinson's disease (PD)	SOCS4	0.23
33	Parkinson's disease (PD)	SYK	0.225
34	Parkinson's disease (PD)	CTLA4	0.225
35	Parkinson's disease (PD)	PRKAG3	0.179
36	breast cancer	FFAR1	0.143
37	acute myeloid leukemia (AML)	PRKAG3	0.132
38	Parkinson's disease (PD)	RAPGEF4	0.131
39	type I diabetes mellitus	PRKAG3	0.122
40	rheumatoid arthritis	PRKAG3	0.122
41	type II diabetes mellitus	DRD1	0.116
42	chronic granulomatous disease	SOCS4	0.076
43	breast cancer	CTLA4	0.062
44	acute myeloid leukemia (AML)	RAPGEF4	0.002
45	type I diabetes mellitus	RAPGEF4	0
46	rheumatoid arthritis	RAPGEF4	0

Supplementary Table S9. Top 100 novel inhibitory target–disease pairs predicted using the trans-disease method with completed data. These predicted targets include not only existing therapeutic targets, but also completely new therapeutic targets that have not any known target–disease associations. Inhibitory targets are listed in decreasing order of predicted scores. Predicted scores represent the therapeutic targetability for the diseases.

Rank	Diseases	Inhibitory targets	Predicted scores
1	adult T-cell leukemia	TAF1B	1.000
2	endometrial Cancer	TAF1B	0.896
3	adult T-cell leukemia	USP9X	0.871
4	type I diabetes mellitus	TAF1B	0.868
5	hepatitis C	TAF1B	0.867
6	endometrial Cancer	USP9X	0.832
7	testicular cancer	TAF1B	0.831
8	type I diabetes mellitus	USP9X	0.826
9	adult T-cell leukemia	TLK1	0.820
10	hepatitis C	USP9X	0.819
11	tuberculosis	TAF1B	0.816
12	adult T-cell leukemia	AATF	0.816
13	adult T-cell leukemia	ADA	0.814
14	hypercholesterolemia	TAF1B	0.813
15	type I diabetes mellitus	CD3D	0.813
16	chronic lymphocytic leukemia (CLL)	TAF1B	0.810
17	type I diabetes mellitus	TLK1	0.809
18	type I diabetes mellitus	ABCC1	0.807
19	hepatitis C	EPCAM	0.807
20	endometrial Cancer	TLK1	0.807

Rank	Diseases	Inhibitory targets	Predicted scores
21	type I diabetes mellitus	A2M	0.807
22	tuberculosis	HRH1	0.806
23	testicular cancer	USP9X	0.806
24	endometrial Cancer	FGFR2	0.806
25	endometrial Cancer	AATF	0.806
26	acute myeloid leukemia (AML)	TAF1B	0.803
27	small cell lung cancer	SHH	0.801
28	hepatitis C	TLK1	0.800
29	hepatitis C	AATF	0.800
30	congenital muscular dystrophies	MAP3K12	0.800
31	aplastic anemia (AA)	PPP3CC	0.800
32	cervical cancer	TGFBR2	0.800
33	type I diabetes mellitus	TNFSF4	0.800
34	small cell lung cancer	MMP14	0.800
35	cervical cancer	ERBB3	0.800
36	small cell lung cancer	MMP1	0.800
37	adult T-cell leukemia	ABAT	0.800
38	congenital muscular dystrophies	SLC2A4RG	0.800
39	congenital muscular dystrophies	XYLB	0.799
40	hepatitis C	ABAT	0.799

Rank	Diseases	Inhibitory targets	Predicted scores
41	cervical cancer	TYMS	0.799
42	congenital muscular dystrophies	ADCK5	0.799
43	Hypercholesterolemia	USP9X	0.799
44	congenital muscular dystrophies	GLYCTK	0.799
45	congenital muscular dystrophies	EPHA10	0.799
46	asthma	IL4R	0.799
47	endometrial Cancer	ABAT	0.799
48	tuberculosis	USP9X	0.799
49	congenital muscular dystrophies	PLA2G12A	0.799
50	inflammatory bowel disease	PPP3CC	0.799
51	congenital muscular dystrophies	P2RY2	0.799
52	congenital muscular dystrophies	NPR1	0.799
53	malignant melanoma	MITF	0.799
54	type I diabetes mellitus	ABCB1	0.799
55	congenital muscular dystrophies	PDGFRL	0.799
56	congenital muscular dystrophies	MAPK11	0.799
57	congenital muscular dystrophies	ASPM	0.799
58	type I diabetes mellitus	ABCA5	0.799
59	congenital muscular dystrophies	STX16	0.799
60	congenital muscular dystrophies	PLK3	0.799
61	congenital muscular dystrophies	MAGI1	0.798
62	congenital muscular dystrophies	CERKL	0.798
63	congenital muscular dystrophies	PIP4K2A	0.798
64	endometrial Cancer	ABCB1	0.798
65	congenital muscular dystrophies	EPHB3	0.798

Rank	Diseases	Inhibitory targets	Predicted scores
66	congenital muscular dystrophies	SMARCA2	0.798
67	congenital muscular dystrophies	MST4	0.798
68	cervical cancer	BRCA1	0.798
69	congenital muscular dystrophies	ZBTB20	0.798
70	congenital muscular dystrophies	RELN	0.798
71	testicular cancer	TUBB6	0.798
72	congenital muscular dystrophies	ICK	0.798
73	congenital muscular dystrophies	ACSL6	0.798
74	congenital muscular dystrophies	AATK	0.798
75	Crohn's disease	ITGB2	0.798
76	congenital muscular dystrophies	CDKL2	0.798
77	congenital muscular dystrophies	TAOK1	0.798
78	congenital muscular dystrophies	MAP3K10	0.798
79	congenital muscular dystrophies	ZNF169	0.798
80	congenital muscular dystrophies	GSC	0.798
81	congenital muscular dystrophies	CD2	0.798
82	type I diabetes mellitus	TRIB3	0.798
83	congenital muscular dystrophies	RNF133	0.798
84	congenital muscular dystrophies	NQO2	0.798
85	congenital muscular dystrophies	PDIK1L	0.798
86	congenital muscular dystrophies	LOC388259	0.798
87	congenital muscular dystrophies	LOC441777	0.798
88	congenital muscular dystrophies	NUMB	0.798
89	congenital muscular dystrophies	MORN2	0.798
90	inflammatory bowel disease	IL23R	0.798

Rank	Diseases	Inhibitory targets	Predicted scores
91	congenital muscular dystrophies	NRXN3	0.798
92	malignant melanoma	BRAF	0.798
93	asthma	TBXA2R	0.798
94	congenital muscular dystrophies	RBM8A	0.798
95	congenital muscular dystrophies	PSAT1	0.798

Rank	Diseases	Inhibitory targets	Predicted scores
96	congenital muscular dystrophies	CDK14	0.798
97	congenital muscular dystrophies	MAP3K6	0.797
98	congenital muscular dystrophies	PTPN5	0.797
99	congenital muscular dystrophies	BMPR2	0.797
100	congenital muscular dystrophies	PRKY	0.797

Supplementary Table S10. Top 100 novel activatory target–disease pairs predicted using the trans-disease method with completed data. These predicted targets include not only existing therapeutic targets, but also completely new therapeutic targets that have not any known target–disease associations. Activatory targets are listed in decreasing order of predicted scores. Predicted scores represent the therapeutic targetability for the diseases.

Rank	Diseases	Activatory targets	Predicted scores
1	Parkinson's disease (PD)	OLIG3	1.000
2	chronic myeloid leukemia (CML)	IFNAR2	0.999
3	multiple myeloma	IFNAR2	0.998
4	Parkinson's disease (PD)	NDUFC2	0.998
5	ovarian cancer	FBXW7	0.995
6	Rett syndrome	MECP2	0.992
7	type II diabetes mellitus	RPS6KA2	0.992
8	type II diabetes mellitus	SIRT1	0.990
9	Parkinson's disease (PD)	FOXO4	0.990
10	type II diabetes mellitus	PDIK1L	0.989
11	type II diabetes mellitus	MEF2C	0.989
12	type II diabetes mellitus	SETMAR	0.988
13	Parkinson's disease (PD)	MYO3B	0.987
14	colorectal cancer	TP53	0.987
15	type II diabetes mellitus	PPARG	0.986
16	Crohn's disease	IL10	0.984
17	inflammatory bowel disease	IL10	0.984
18	Parkinson's disease (PD)	BCR-ABL	0.983
19	Parkinson's disease (PD)	ZNF384	0.983
20	Parkinson's disease (PD)	FLJ25006	0.983

Rank	Diseases	Activatory targets	Predicted scores
21	Parkinson's disease (PD)	HMGB4	0.983
22	Parkinson's disease (PD)	DDAH2	0.983
23	type II diabetes mellitus	BCR-ABL	0.982
24	type II diabetes mellitus	CLK1	0.981
25	Parkinson's disease (PD)	TRIM27	0.981
26	Parkinson's disease (PD)	MAGEA10	0.981
27	Parkinson's disease (PD)	MATK	0.981
28	chronic granulomatous disease	SYF2	0.981
29	type II diabetes mellitus	HEY1	0.981
30	type II diabetes mellitus	POLD1	0.981
31	Parkinson's disease (PD)	PRKAR2A	0.980
32	Parkinson's disease (PD)	RAF1	0.980
33	type II diabetes mellitus	CDIPT	0.979
34	Parkinson's disease (PD)	ETV5	0.979
35	Parkinson's disease (PD)	ZNF582	0.979
36	Parkinson's disease (PD)	DLX3	0.979
37	type II diabetes mellitus	FBXL10	0.979
38	type II diabetes mellitus	EGFR	0.979
39	chronic granulomatous disease	ASPHD1	0.979
40	Parkinson's disease (PD)	PRDM14	0.979

Rank	Diseases	Activatory targets	Predicted scores
41	type II diabetes mellitus	BRAF	0.979
42	type II diabetes mellitus	GPR119	0.979
43	type II diabetes mellitus	TP53RK	0.978
44	Parkinson's disease (PD)	CREB3	0.978
45	type II diabetes mellitus	NAP1L1	0.978
46	Parkinson's disease (PD)	ZNF268	0.978
47	Parkinson's disease (PD)	NDUFB8	0.978
48	type II diabetes mellitus	GMEB1	0.978
49	Parkinson's disease (PD)	ZNF592	0.978
50	type II diabetes mellitus	EHMT2	0.978
51	chronic granulomatous disease	C2	0.977
52	Parkinson's disease (PD)	ATOH1	0.977
53	Parkinson's disease (PD)	FOXN2	0.977
54	type II diabetes mellitus	ESR2	0.977
55	chronic granulomatous disease	ZNF22	0.977
56	Parkinson's disease (PD)	NEK9	0.977
57	Parkinson's disease (PD)	IRF5	0.977
58	Parkinson's disease (PD)	DEPDC1	0.977
59	type II diabetes mellitus	FARSA	0.977
60	Parkinson's disease (PD)	NR1I3	0.977
61	Parkinson's disease (PD)	MAP3K6	0.977
62	type II diabetes mellitus	SGK2	0.977
63	chronic granulomatous disease	WDTC1	0.976
64	Parkinson's disease (PD)	ALK	0.976
65	Parkinson's disease (PD)	BID	0.976

Rank	Diseases	Activatory targets	Predicted scores
66	Parkinson's disease (PD)	ZIK1	0.976
67	renal cell carcinoma	IL2RB	0.976
68	Parkinson's disease (PD)	LRRC4	0.976
69	type II diabetes mellitus	CUL5	0.976
70	Parkinson's disease (PD)	CDC42SE1	0.976
71	Parkinson's disease (PD)	PCK2	0.976
72	Parkinson's disease (PD)	CREM	0.976
73	chronic granulomatous disease	RXFP1	0.976
74	type II diabetes mellitus	HESX1	0.976
75	chronic granulomatous disease	RG9MTD3	0.976
76	acute myeloid leukemia (AML)	SYF2	0.976
77	Parkinson's disease (PD)	AP1S2	0.976
78	chronic granulomatous disease	TIMM17B	0.976
79	Parkinson's disease (PD)	RFX3	0.976
80	type II diabetes mellitus	ZNF223	0.976
81	Parkinson's disease (PD)	ZNF74	0.976
82	type II diabetes mellitus	PPARD	0.976
83	Parkinson's disease (PD)	MAP3K8	0.975
84	renal cell carcinoma	IFNGR1	0.975
85	type II diabetes mellitus	PHF23	0.975
86	rheumatoid arthritis	SYF2	0.975
87	type I diabetes mellitus	SYF2	0.975
88	chronic granulomatous disease	TXNIP	0.975
89	Parkinson's disease (PD)	NEU1	0.975
90	type II diabetes mellitus	GOT1	0.975

Rank	Diseases	Activatory targets	Predicted scores
91	Parkinson's disease (PD)	STK32C	0.975
92	type II diabetes mellitus	FOXP4	0.975
93	Parkinson's disease (PD)	MXI1	0.975
94	acute myeloid leukemia (AML)	ASPHD1	0.975
95	chronic granulomatous disease	TUBB4B	0.975

Rank	Diseases	Activatory targets	Predicted scores
96	type II diabetes mellitus	HSD17B4	0.974
97	Parkinson's disease (PD)	TFDP1	0.974
98	rheumatoid arthritis	ASPHD1	0.974
99	type I diabetes mellitus	ASPHD1	0.974
100	Parkinson's disease (PD)	SMYD1	0.974

Supplementary Table S11. Performance evaluation of data completion by tensor decomposition algorithms for third-order transcriptome data (knocked down genes, genes, and cell lines) with different rates of artificial missing values. Missing values are generated by the "random missing" strategy. Relative standard errors (RSEs) between the original and reconstructed data from tensor decomposition are calculated for (a) all values and (b) missing values only. The tensor decomposition method used in this study and the baseline method are denoted as TT (Yuan *et al.*, 2017) and CP (Acar *et al.*, 2010), respectively. The optimized tensor ranks are shown for each method. Artificially generated missing rates: 10%, 50%, and 90% are tested. Cell lines are listed in order of increasing original missing rates.

	Artificial missing rate											
	10%				50%				90%			
	CP (baseline)	CP-ranks	TT (this study)	TT-ranks	CP (baseline)	CP-ranks	TT (this study)	TT-ranks	CP (baseline)	CP-ranks	TT (this study)	TT-ranks
**(a) RSEs for all values**												
total cell lines	0.0476	30	**0.0455**	{1,30,30,1}	0.0496	30	**0.0472**	{1,20,20,1}	0.0488	10	**0.0479**	{1,30,30,1}
PC3	0.0613	30	**0.0599**	{1,30,30,1}	0.0629	30	**0.0609**	{1,30,30,1}	0.0626	10	**0.0616**	{1,10,10,1}
MCF7	0.0620	30	**0.0597**	{1,30,30,1}	0.0637	30	**0.0616**	{1,30,30,1}	0.0633	10	**0.0624**	{1,10,10,1}
HA1E	0.0632	30	**0.0579**	{1,30,30,1}	0.0652	30	**0.0628**	{1,20,20,1}	0.0652	10	**0.0639**	{1,30,30,1}
A375	0.0676	30	**0.0634**	{1,30,30,1}	0.0696	30	**0.0673**	{1,30,30,1}	0.0695	10	**0.0680**	{1,10,10,1}
A549	0.0662	30	**0.0615**	{1,30,30,1}	0.0683	30	**0.0659**	{1,20,20,1}	0.0684	10	**0.0669**	{1,30,30,1}
HEPG2	0.0616	30	**0.0590**	{1,30,30,1}	0.0634	30	**0.0611**	{1,20,20,1}	0.0632	10	**0.0623**	{1,30,30,1}
VCAP	0.0607	30	**0.0589**	{1,30,30,1}	0.0624	30	**0.0598**	{1,20,20,1}	0.0620	10	**0.0612**	{1,30,30,1}
HT29	0.0612	20	**0.0592**	{1,30,30,1}	0.0632	30	**0.0606**	{1,20,20,1}	0.0625	10	**0.0617**	{1,30,30,1}
HCC515	0.0528	30	**0.0515**	{1,30,30,1}	0.0550	30	**0.0527**	{1,30,30,1}	0.0539	10	**0.0532**	{1,30,30,1}
HEKTE	0.0241	30	**0.0238**	{1,10,10,1}	0.0269	30	**0.0238**	{1,20,20,1}	0.0247	10	**0.0238**	{1,20,20,1}
NPC	0.0367	30	**0.0356**	{1,30,30,1}	0.0386	30	**0.0367**	{1,10,10,1}	0.0373	10	**0.0369**	{1,20,20,1}
SW480	0.0183	30	**0.0178**	{1,10,10,1}	0.0223	30	**0.0178**	{1,20,20,1}	0.0190	10	**0.0178**	{1,10,10,1}
ASC	0.0316	30	**0.0313**	{1,30,30,1}	0.0341	30	**0.0314**	{1,10,10,1}	0.0321	10	**0.0315**	{1,20,20,1}
SHSY5Y	0.0123	20	**0.0117**	{1,10,10,1}	0.0173	30	**0.0117**	{1,20,20,1}	0.0134	10	**0.0116**	{1,10,10,1}
SKL	0.0209	30	**0.0206**	{1,10,10,1}	0.0243	30	**0.0206**	{1,20,20,1}	0.0216	10	**0.0206**	{1,20,20,1}
HEK293T	0.0061	30	**0.0047**	{1,10,10,1}	0.0137	30	**0.0046**	{1,10,10,1}	0.0080	10	**0.0044**	{1,10,10,1}
NCIH716	0.0043	20	**0.0016**	{1,10,10,1}	0.0138	30	**0.0016**	{1,20,20,1}	0.0068	10	**0.0010**	{1,10,10,1}
**(b) RSEs for missing values**												
total cell lines	0.0699	30	**0.0668**	{1,30,30,1}	0.0719	30	**0.0695**	{1,20,20,1}	0.0717	10	**0.0707**	{1,30,30,1}
PC3	0.0656	30	**0.0641**	{1,30,30,1}	0.0674	30	**0.0651**	{1,20,20,1}	0.0670	10	**0.0659**	{1,10,10,1}
MCF7	0.0675	30	**0.0650**	{1,30,30,1}	0.0693	30	**0.0670**	{1,30,30,1}	0.0690	10	**0.0680**	{1,10,10,1}
HA1E	0.0686	20	**0.0628**	{1,30,30,1}	0.0709	30	**0.0681**	{1,30,30,1}	0.0710	10	**0.0695**	{1,30,30,1}
A375	0.0732	20	**0.0687**	{1,30,30,1}	0.0756	30	**0.0730**	{1,30,30,1}	0.0755	10	**0.0739**	{1,10,10,1}
A549	0.0721	30	**0.0669**	{1,30,30,1}	0.0747	30	**0.0719**	{1,20,20,1}	0.0749	10	**0.0733**	{1,30,30,1}
HEPG2	0.0698	30	**0.0667**	{1,30,30,1}	0.0721	30	**0.0693**	{1,20,20,1}	0.0720	10	**0.0710**	{1,30,30,1}
VCAP	0.0672	30	**0.0650**	{1,30,30,1}	0.0689	30	**0.0660**	{1,20,20,1}	0.0686	10	**0.0677**	{1,30,30,1}
HT29	0.0698	30	**0.0674**	{1,30,30,1}	0.0721	30	**0.0691**	{1,20,20,1}	0.0715	10	**0.0707**	{1,30,30,1}
HCC515	0.0703	10	**0.0687**	{1,30,30,1}	0.0726	30	**0.0705**	{1,30,30,1}	0.0720	10	**0.0713**	{1,30,30,1}
HEKTE	**0.0786**	10	0.0790	{1,20,20,1}	0.0801	30	**0.0795**	{1,10,10,1}	0.0800	10	**0.0797**	{1,20,20,1}
NPC	0.0759	10	**0.0733**	{1,30,30,1}	0.0775	30	**0.0766**	{1,30,30,1}	0.0772	10	**0.0771**	{1,20,20,1}
SW480	0.0764	10	**0.0761**	{1,30,30,1}	0.0771	30	**0.0763**	{1,30,30,1}	0.0773	10	**0.0769**	{1,30,30,1}
ASC	0.0727	10	0.0727	{1,30,30,1}	0.0744	30	**0.0734**	{1,30,30,1}	0.0741	10	**0.0738**	{1,20,20,1}
SHSY5Y	**0.0741**	10	0.0748	{1,20,20,1}	0.0750	30	**0.0742**	{1,20,20,1}	0.0751	10	**0.0747**	{1,20,20,1}
SKL	0.0727	30	**0.0722**	{1,30,30,1}	0.0746	30	**0.0732**	{1,10,10,1}	0.0738	10	**0.0737**	{1,10,10,1}
HEK293T	**0.0827**	20	0.0831	{1,20,20,1}	0.0856	30	**0.0852**	{1,10,10,1}	0.0868	10	**0.0865**	{1,30,30,1}
NCIH716	0.0505	30	**0.0498**	{1,20,20,1}	0.0531	10	**0.0521**	{1,20,20,1}	0.0552	10	**0.0539**	{1,20,20,1}

Supplementary Table S12. Performance evaluation of data completion by tensor decomposition algorithms for third-order transcriptome data (overexpressed genes, genes, and cell lines) with different rates of artificial missing values. Missing values are generated by the "random missing" strategy. Relative standard errors (RSEs) between the original and reconstructed data from tensor decomposition are calculated for (a) all values and (b) missing values only. The tensor decomposition method used in this study and the baseline method are denoted as TT (Yuan *et al.*, 2017) and CP (Acar *et al.*, 2010), respectively. The optimized tensor ranks are shown for each method. Artificially generated missing rates: 10%, 50%, and 90% are tested. Cell lines are listed in order of increasing original missing rates.

	Artificial missing rate											
	10%				50%				90%			
	CP (baseline)	CP-ranks	TT (this study)	TT-ranks	CP (baseline)	CP-ranks	TT (this study)	TT-ranks	CP (baseline)	CP-ranks	TT (this study)	TT-ranks
**(a) RSEs for all values**												
total cell lines	0.0820	30	**0.0768**	{1,20,20,1}	0.0821	20	**0.0796**	{1,30,30,1}	0.0830	30	**0.0824**	{1,30,30,1}
PC3	0.0843	30	**0.0807**	{1,30,30,1}	0.0843	20	**0.0820**	{1,30,30,1}	0.0855	30	**0.0843**	{1,30,30,1}
A375	0.0877	30	**0.0811**	{1,30,30,1}	0.0880	20	**0.0831**	{1,30,30,1}	0.0891	30	**0.0881**	{1,30,30,1}
HEPG2	0.0887	30	**0.0835**	{1,20,20,1}	0.0888	20	**0.0867**	{1,30,30,1}	0.0896	30	**0.0888**	{1,30,30,1}
HT29	0.0834	30	**0.0783**	{1,20,20,1}	0.0835	20	**0.0814**	{1,30,30,1}	0.0843	30	**0.0840**	{1,30,30,1}
HA1E	0.0796	30	**0.0718**	{1,20,20,1}	0.0796	20	**0.0770**	{1,30,30,1}	0.0806	30	**0.0801**	{1,30,30,1}
MCF7	0.0795	30	**0.0748**	{1,30,30,1}	0.0795	20	**0.0775**	{1,30,30,1}	0.0804	30	**0.0797**	{1,30,30,1}
HCC515	0.0854	30	**0.0805**	{1,30,30,1}	0.0855	20	**0.0835**	{1,30,30,1}	0.0864	30	**0.0859**	{1,30,30,1}
A549	0.0858	30	**0.0790**	{1,20,20,1}	0.0859	20	**0.0826**	{1,30,30,1}	**0.0866**	30	0.0867	{1,30,30,1}
VCAP	0.0751	30	**0.0706**	{1,20,20,1}	0.0751	20	**0.0736**	{1,30,30,1}	0.0755	30	**0.0754**	{1,10,10,1}
HEK293T	0.0683	30	**0.0649**	{1,20,20,1}	0.0689	20	**0.0668**	{1,30,30,1}	0.0690	30	**0.0682**	{1,20,20,1}
**(b) RSEs for missing values**												
total cell lines	0.1013	30	**0.0951**	{1,20,20,1}	0.1017	20	**0.0987**	{1,30,30,1}	0.1028	30	**0.1022**	{1,30,30,1}
PC3	0.0980	30	**0.0940**	{1,30,30,1}	0.0984	20	**0.0957**	{1,30,30,1}	0.0997	30	**0.0983**	{1,30,30,1}
A375	0.1016	30	**0.0943**	{1,30,30,1}	0.1025	20	**0.0969**	{1,30,30,1}	0.1040	30	**0.1027**	{1,30,30,1}
HEPG2	0.1056	30	**0.0995**	{1,30,30,1}	0.1063	20	**0.1036**	{1,30,30,1}	0.1073	30	**0.1062**	{1,30,30,1}
HT29	0.1001	30	**0.0939**	{1,20,20,1}	0.1001	20	**0.0978**	{1,30,30,1}	0.1010	30	**0.1007**	{1,30,30,1}
HA1E	0.0948	30	**0.0859**	{1,20,20,1}	0.0954	20	**0.0922**	{1,30,30,1}	0.0966	30	**0.0960**	{1,30,30,1}
MCF7	0.0945	10	**0.0892**	{1,30,30,1}	0.0954	20	**0.0929**	{1,30,30,1}	0.0965	10	**0.0956**	{1,30,30,1}
HCC515	0.1015	30	**0.0956**	{1,30,30,1}	0.1023	20	**0.0999**	{1,30,30,1}	0.1035	30	**0.1028**	{1,30,30,1}
A549	0.1118	30	**0.1032**	{1,20,20,1}	0.1123	30	**0.1077**	{1,30,30,1}	**0.1135**	30	0.1137	{1,30,30,1}
VCAP	0.0981	30	**0.0921**	{1,20,20,1}	0.0983	20	**0.0963**	{1,30,30,1}	**0.0989**	30	0.0990	{1,10,10,1}
HEK293T	0.1085	10	**0.1041**	{1,20,20,1}	0.1101	30	**0.1078**	{1,30,30,1}	0.1107	30	**0.1106**	{1,20,20,1}
